# Supplementary material for: Sesquiterpenoids and Xanthones from the Kiwifruit-Associated Fungus Bipolaris sp. and Their Anti-Pathogenic Microorganism Activity
Source: J Fungi (Basel). 2021 Dec 23;8(1):9. doi: 10.3390/jof8010009 (PMC8781276; doi:10.3390/jof8010009)
Supplement: Supplementary file 1 [file jof-08-00009-s001.zip › Supporting Information.pdf]

Supporting information for

Sesquiterpenoids and Xanthones from Kiwi-Associated Fungus *Bipolaris* sp. and Their Anti-Pathogenic Microorganism Activity

Jun-Jie Yu, Ying-Xue Jin, Shan-Shan Huang, and Juan He*

School of Pharmaceutical Sciences, National Demonstration Center for Experimental Ethnopharmacology Education, South-Central University for Nationalities, Wuhan 430074, People's Republic of China; junjieyu98@outlook.com (J. J. Y); Yancy6020@163.com (Y. X. J.); HuangSS1998@126.com (S. S. H.)

*Correspondence: 2015048@mail.scuec.edu.cn (J. H.)

Content

Sections S1. Supplementary of NMR, HRESIMS and CD spectra
- **S1.1 NMR and HRESIMS spectra of bipolarisorokin A (1)**
- **S1.2 NMR and HRESIMS spectra of bipolarisorokin B (2)**
- **S1.3 NMR, HRESIMS and CD spectra of bipolarisorokin C (3)**
- **S1.4 NMR, HRESIMS and CD spectra of bipolarisorokin D (4)**
- **S1.5 NMR, HRESIMS and CD spectra of bipolarisorokin E (5)**
- **S1.6 NMR and HRESIMS spectra of bipolarisorokin F (6)**
- **S1.7 NMR, HRESIMS and CD spectra of bipolarisorokin G (7)**
- **S1.8 NMR, HRESIMS and CD spectra of bipolarisorokin H (8)**
- **S1.9 NMR and HRESIMS spectra of bipolarisorokin I (9)**
- **S1.10 NMR, HRESIMS and CD spectra of bipolarithone A (10)**
- **S1.11 NMR, HRESIMS and CD spectra of bipolarithone B (11)**
- **S1.12 NMR, HRESIMS and CD spectra of bipolarithone C (12)**
- **S1.13 NMR, HRESIMS and CD spectra of bipolarithone D (13)**

Sections S2. Computational details
- **S2.1 Computational details for bipolarisorokin C (3) (ECD)**
- **S2.2 Computational details for bipolarisorokin G (7) (ECD)**
- **S2.3 Computational details for bipolarithone A (10) (ECD)**
- **S2.4 Computational details for bipolarithone B (11) (ECD)**
- **S2.5 Computational details for bipolarithone C (12) (ECD)**
- **S2.6 Computational details for 12a and 12d (NMR)**
- **S2.7 Computational details for bipolarithone D (13) (ECD)**

Sections S1. Supplementary of NMR, HRESIMS and CD spectra

S1.1 NMR and HRESIMS spectra of bipolarisorokin A (1)

^{1}H NMR spectrum

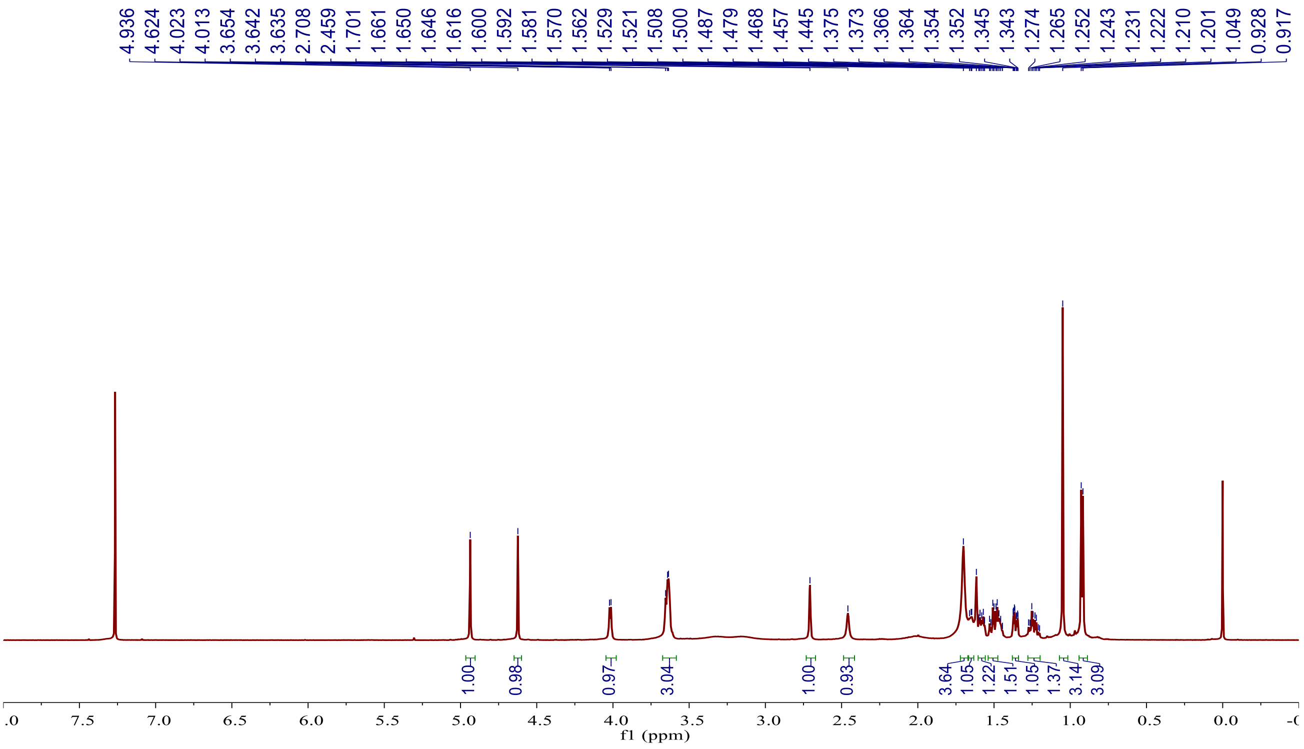

^{13}C NMR and DEPT spectra

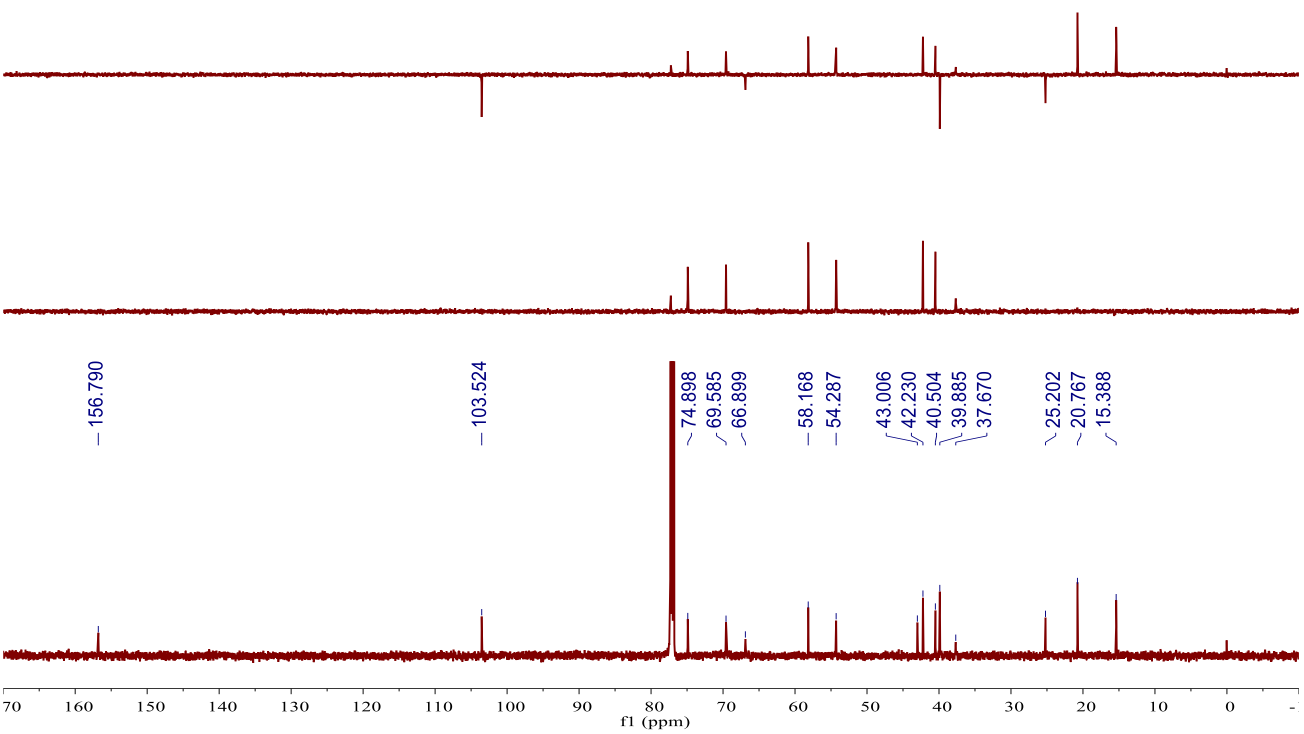

HSQC spectrum

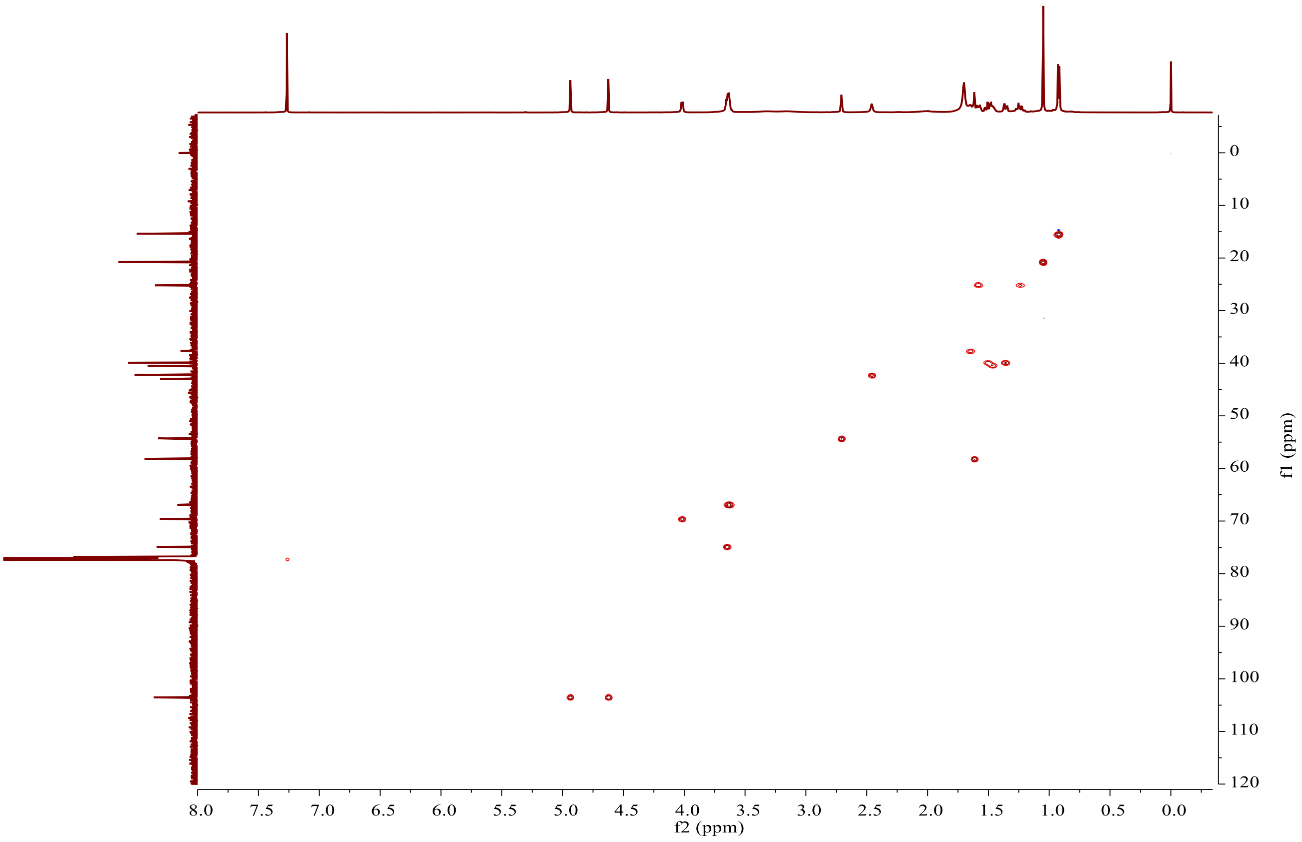

HMBC spectrum

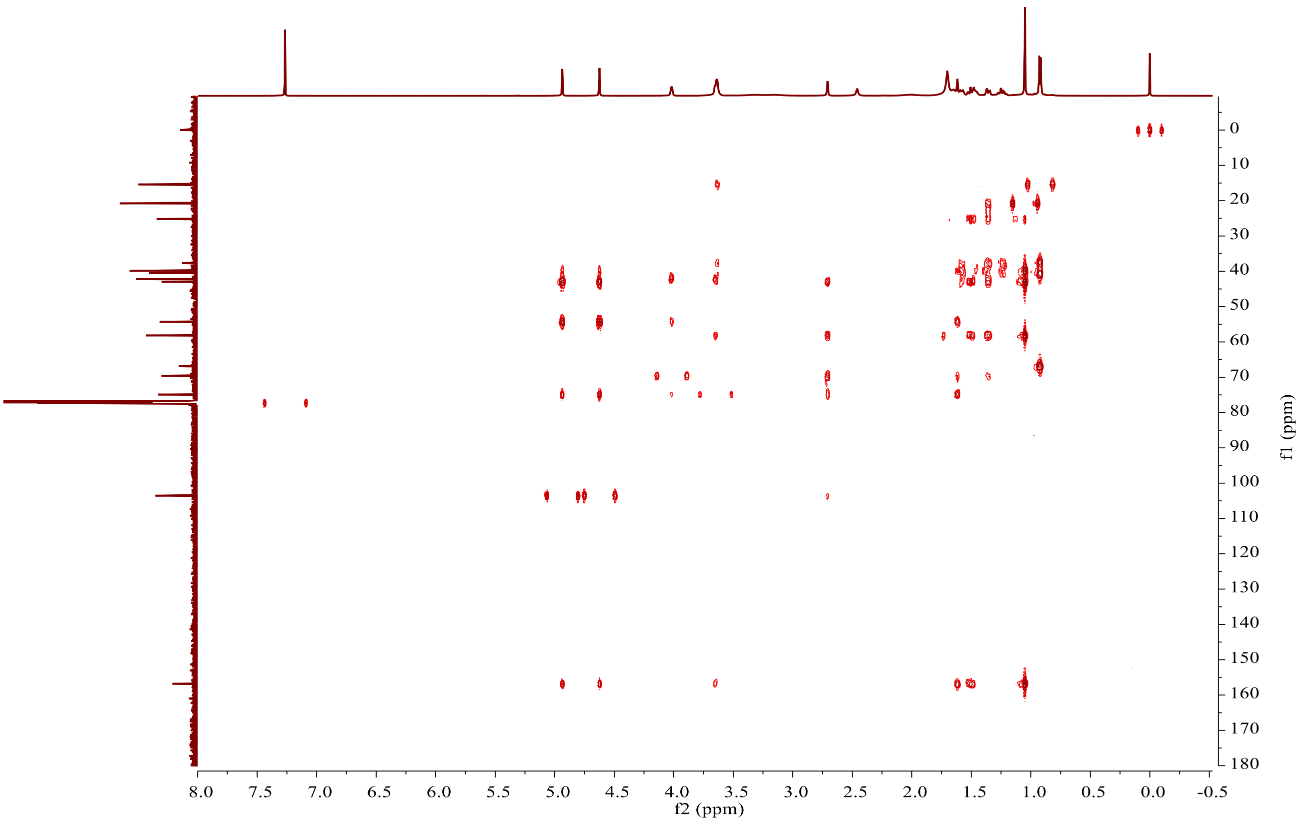

^{1}H-^{1}H COSY spectrum

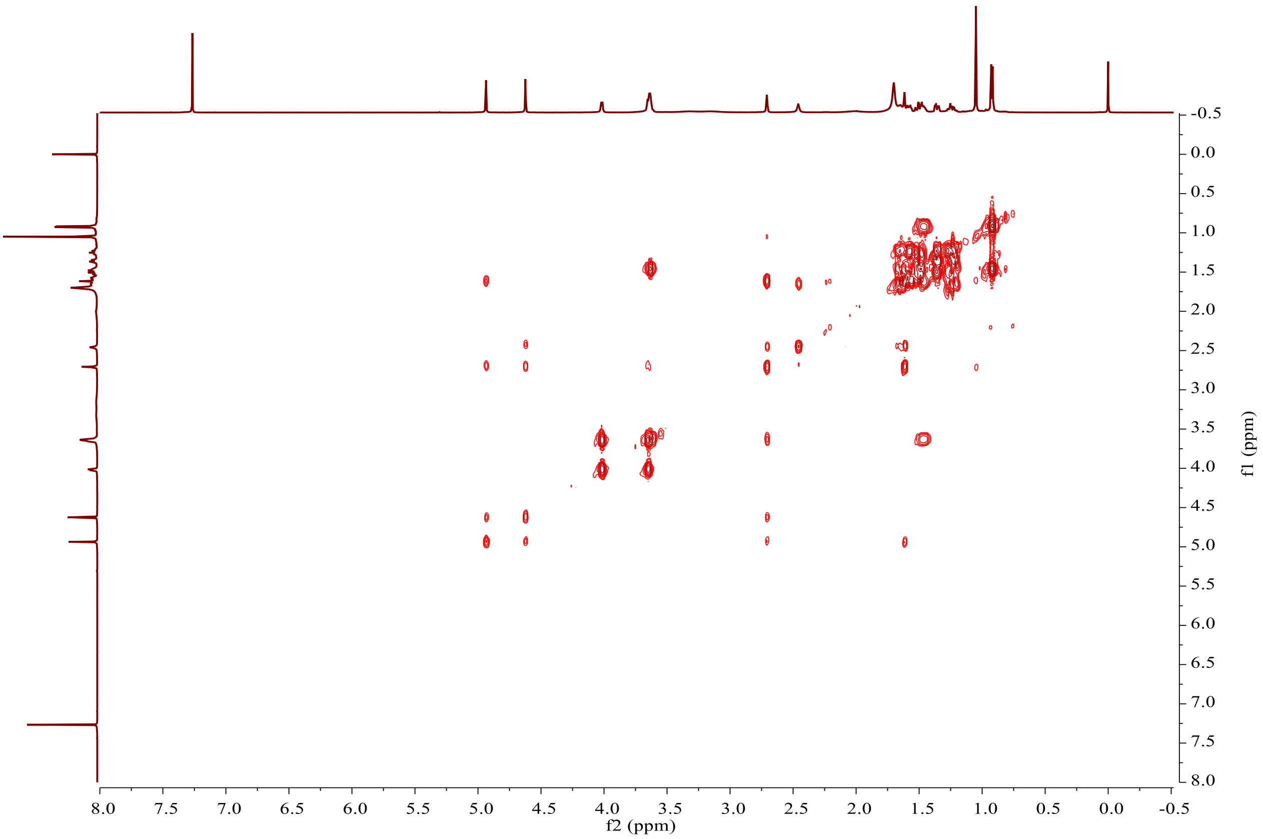

ROESY spectrum

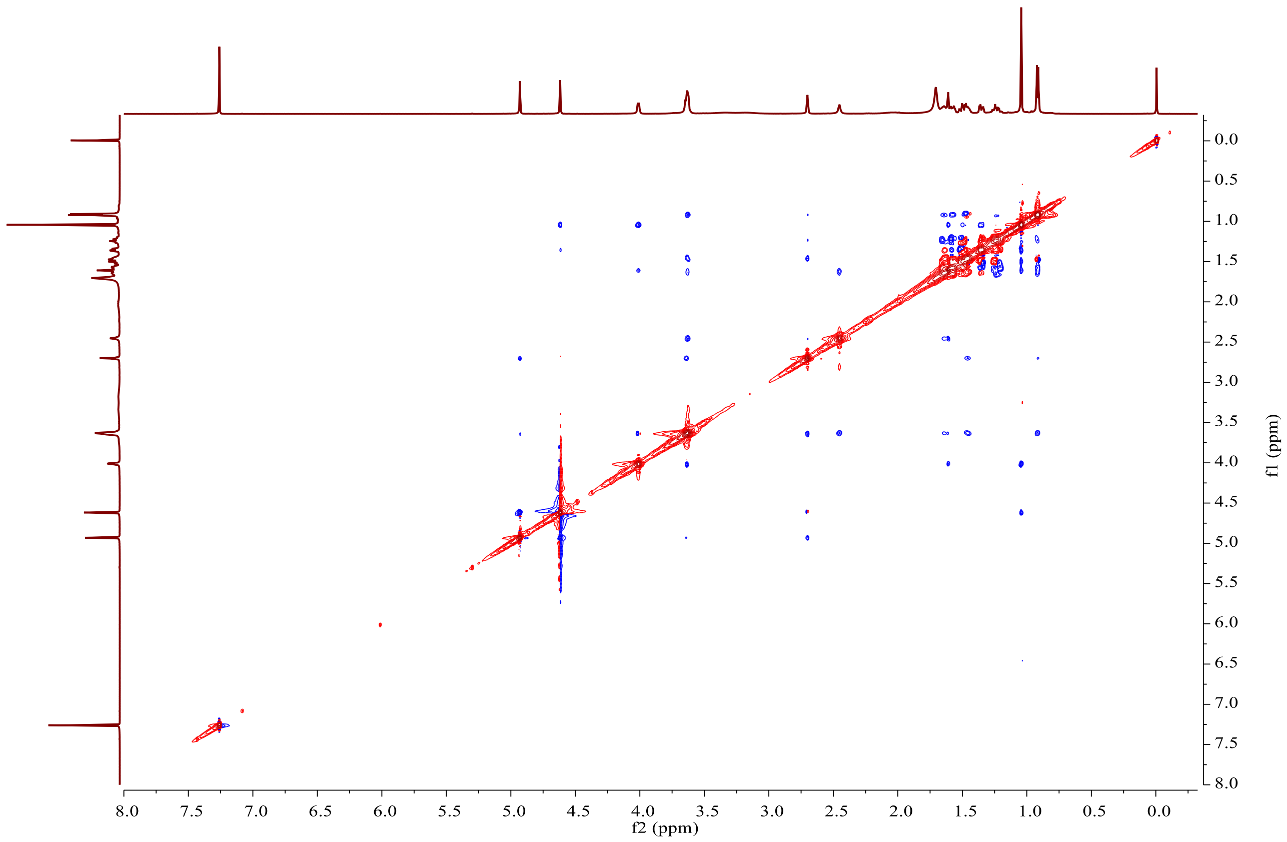

HRESIMS

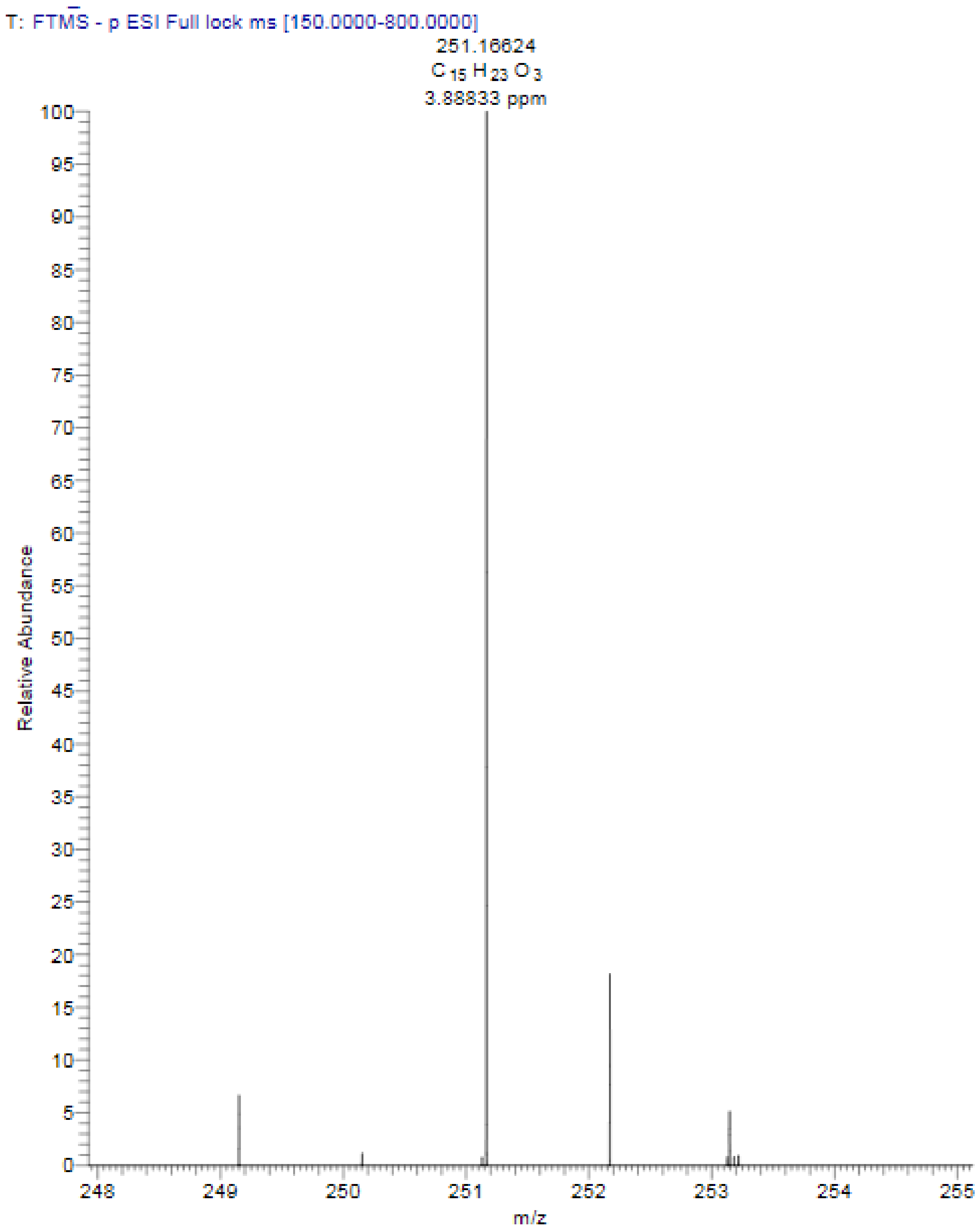
T: FTMS - p ESI Full lock ms [150.0000-800.0000]
251.16624
C 15 H 23 O 3
3.88833 ppm
Relative Abundance
100
95
90
85
80
75
70
65
60
55
50
45
40
35
30
25
20
15
10
5
0
248
249
250
251
252
253
254
255
m/z

S1.2 NMR and HRESIMS spectra of bipolarisorokin B (2)

^{1}H NMR spectrum

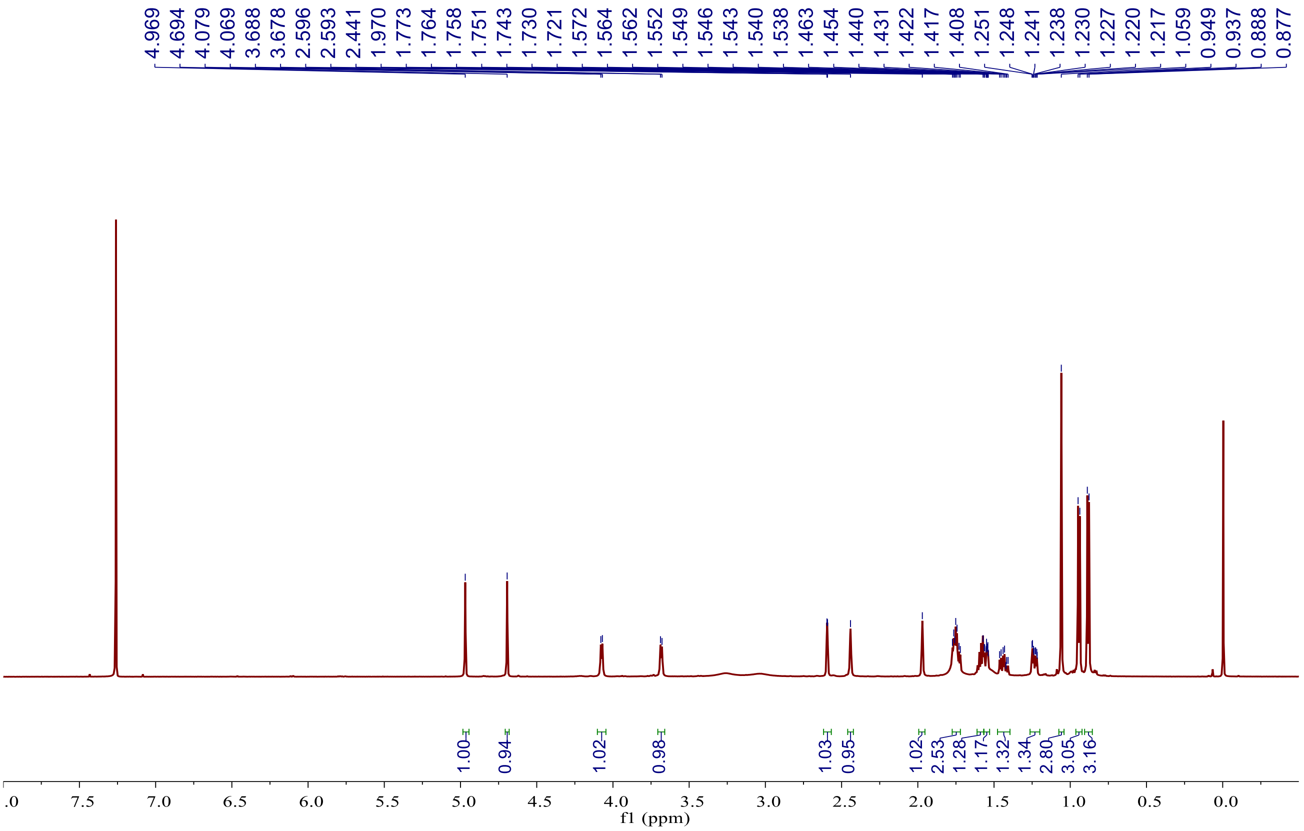

^{13}C NMR and DEPT spectra

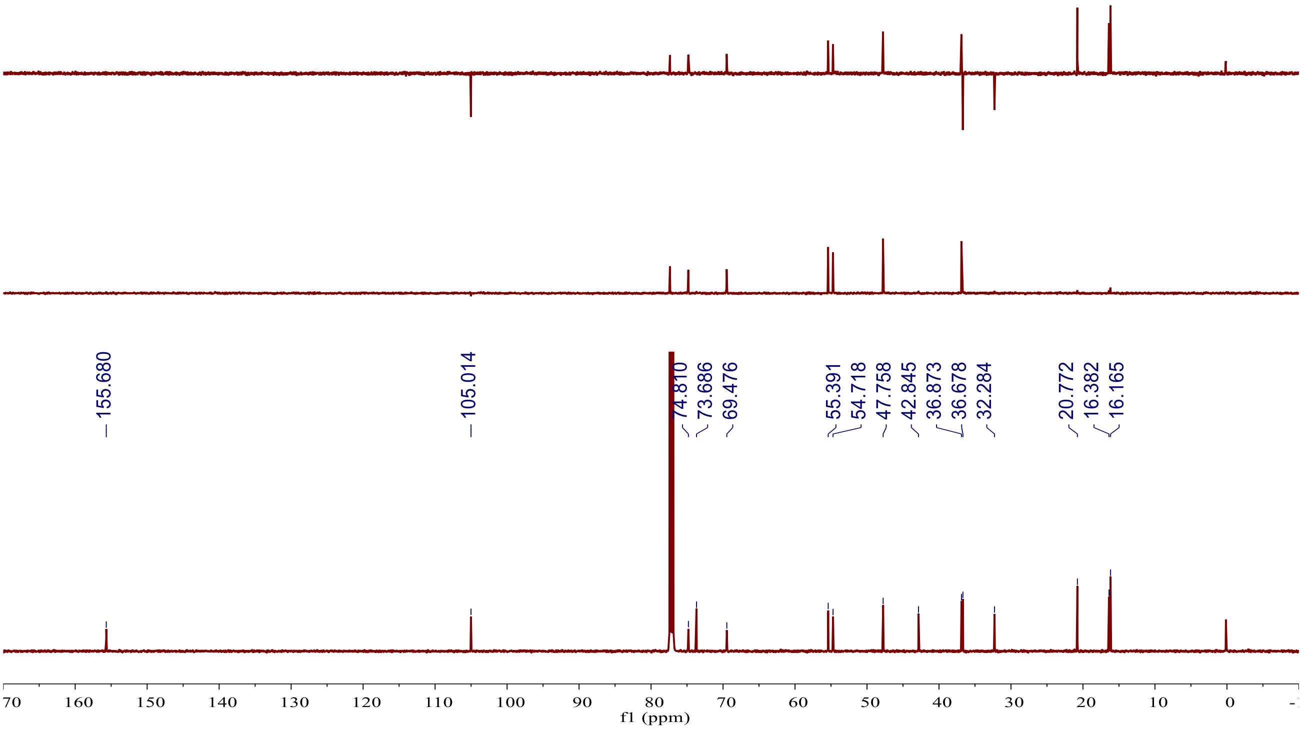

HSQC spectrum

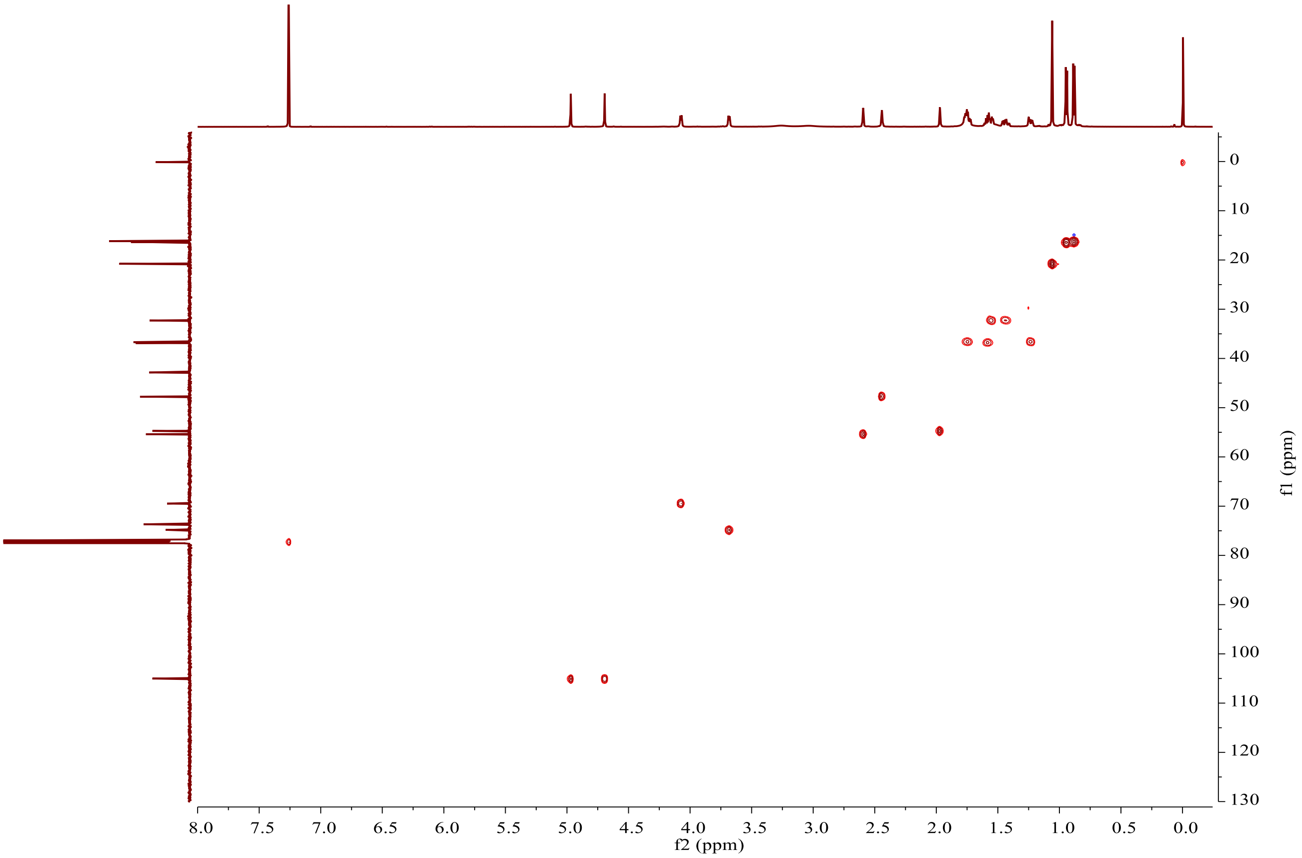

HMBC spectrum

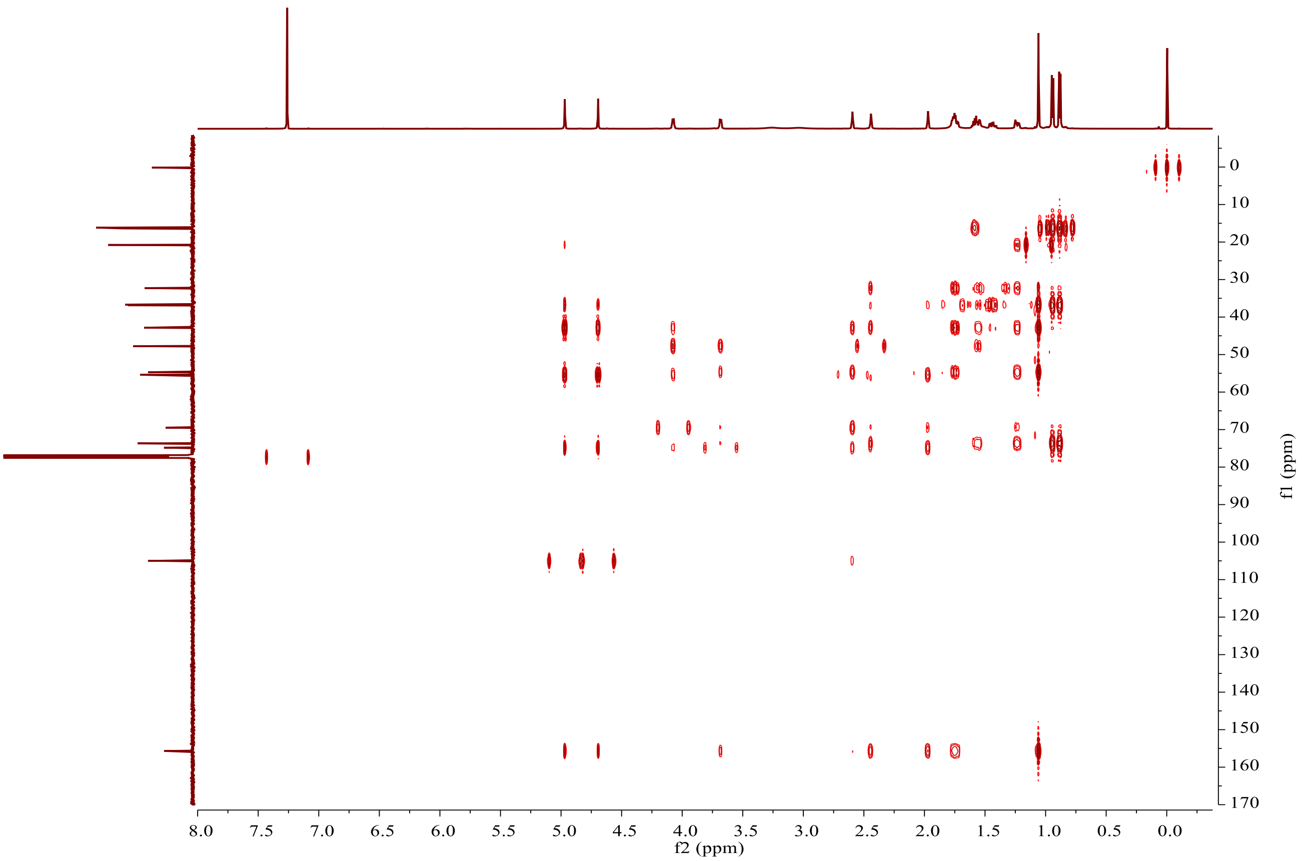

¹H-¹H COSY spectrum

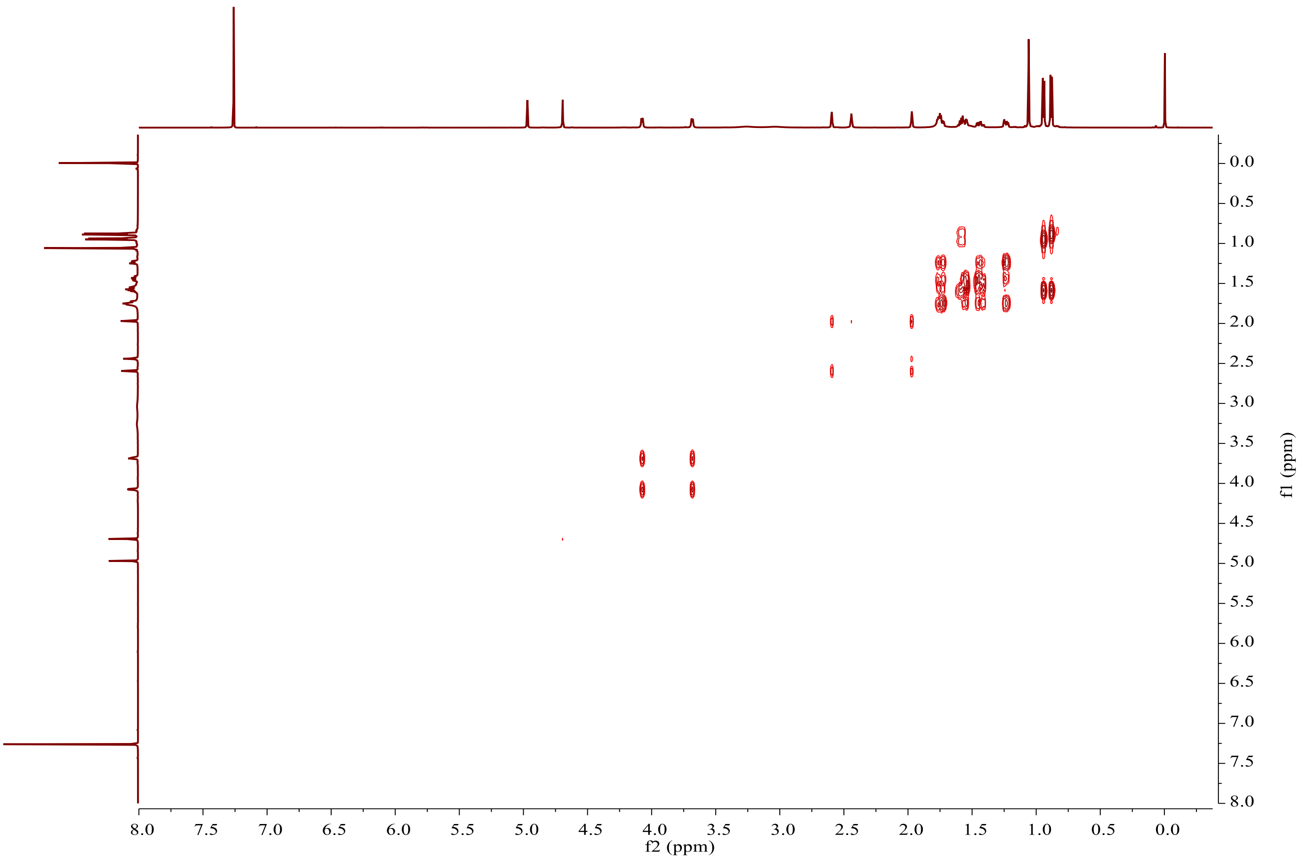

ROESY spectrum

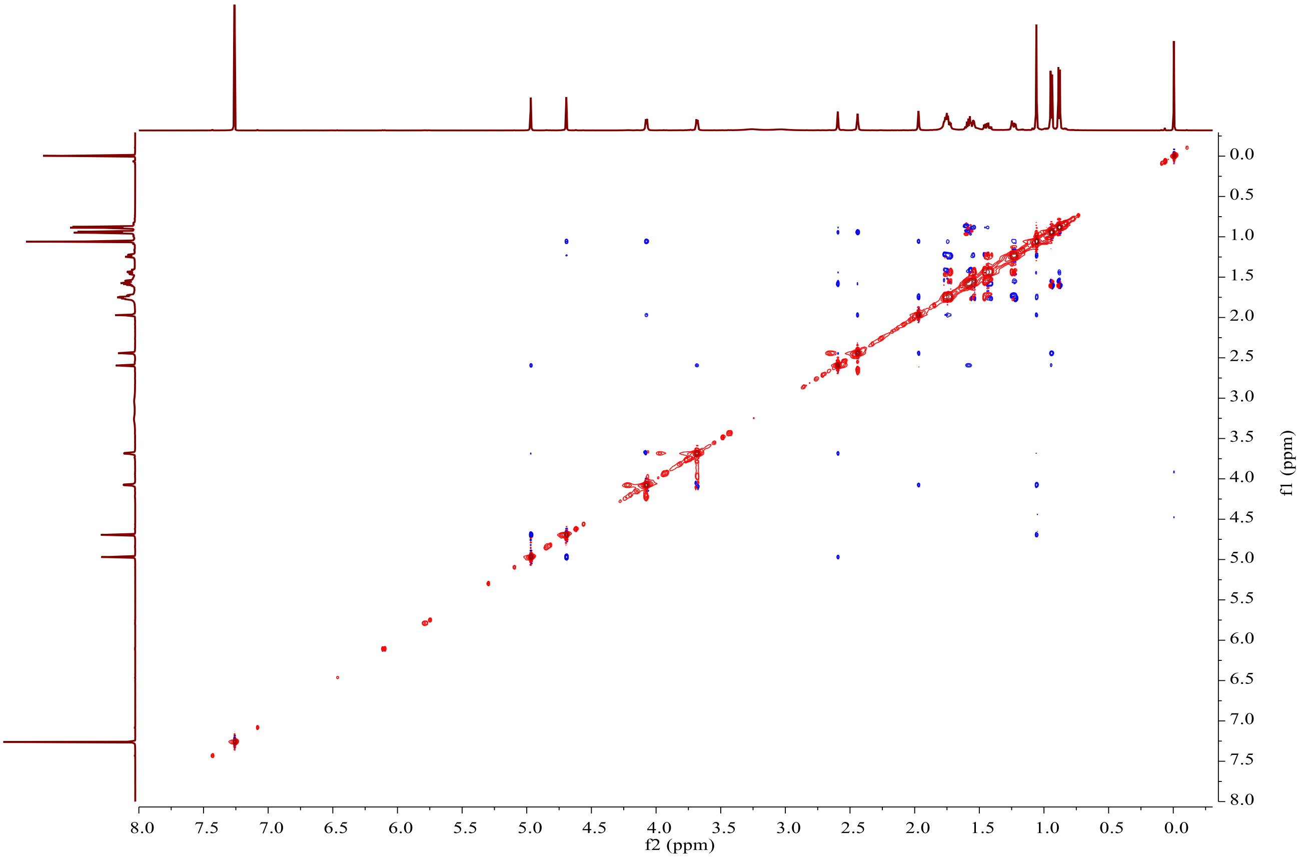

HRESIMS

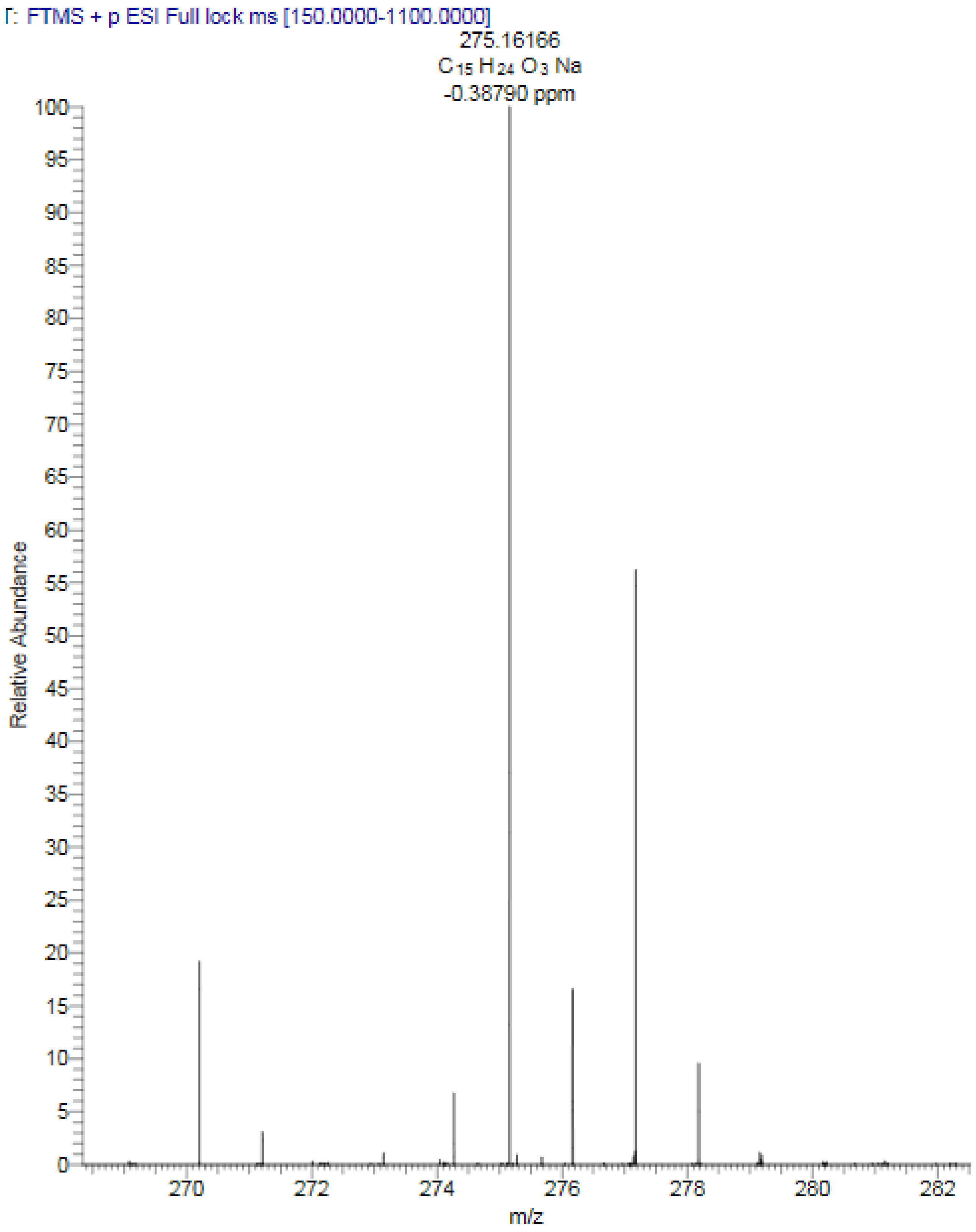
T: FTMS + p ESI Full lock ms [150.0000-1100.0000]
275.16166
C 15 H 24 O 3 Na
-0.38790 ppm
Relative Abundance
m/z

S1.3 NMR, HRESIMS and CD spectra of bipolarisorokin C (3)

^{1}H NMR spectrum

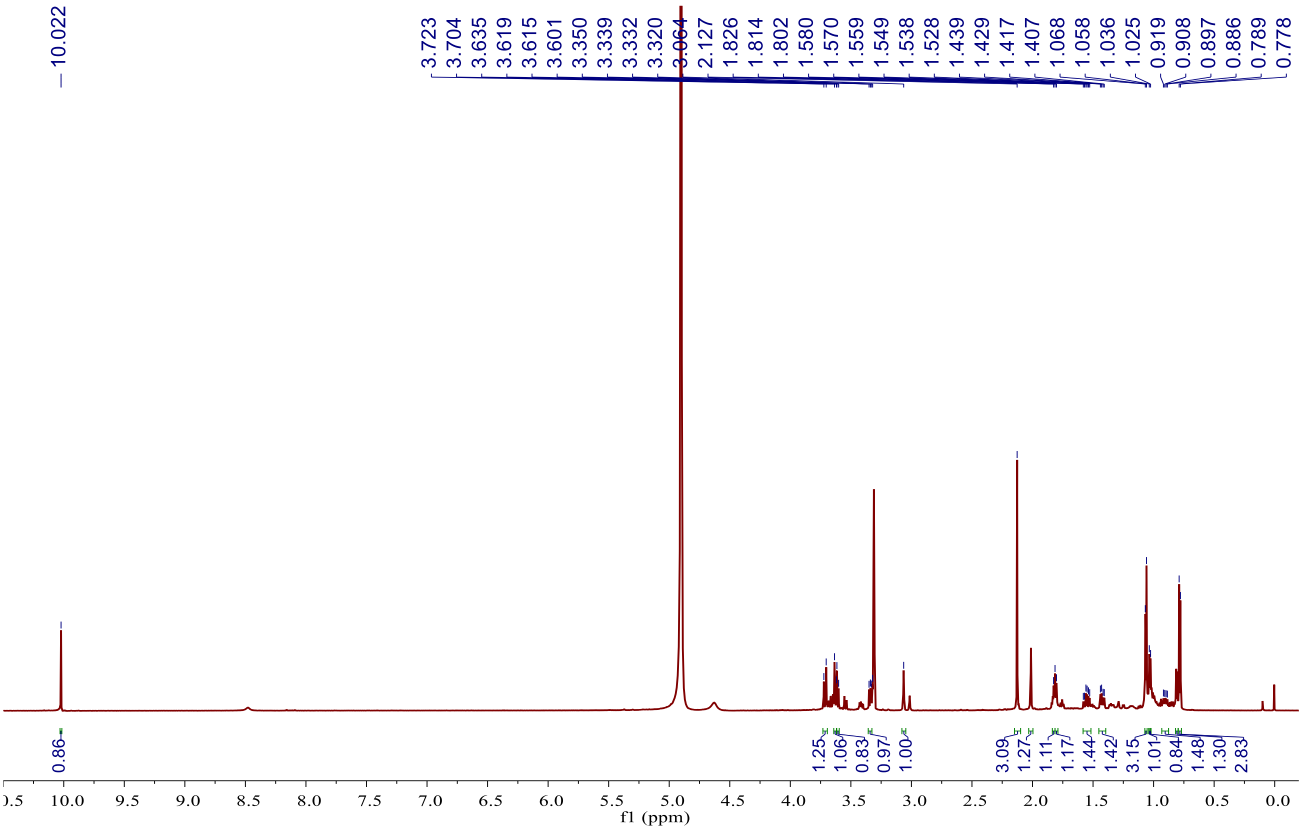

^{13}C NMR and DEPT spectra

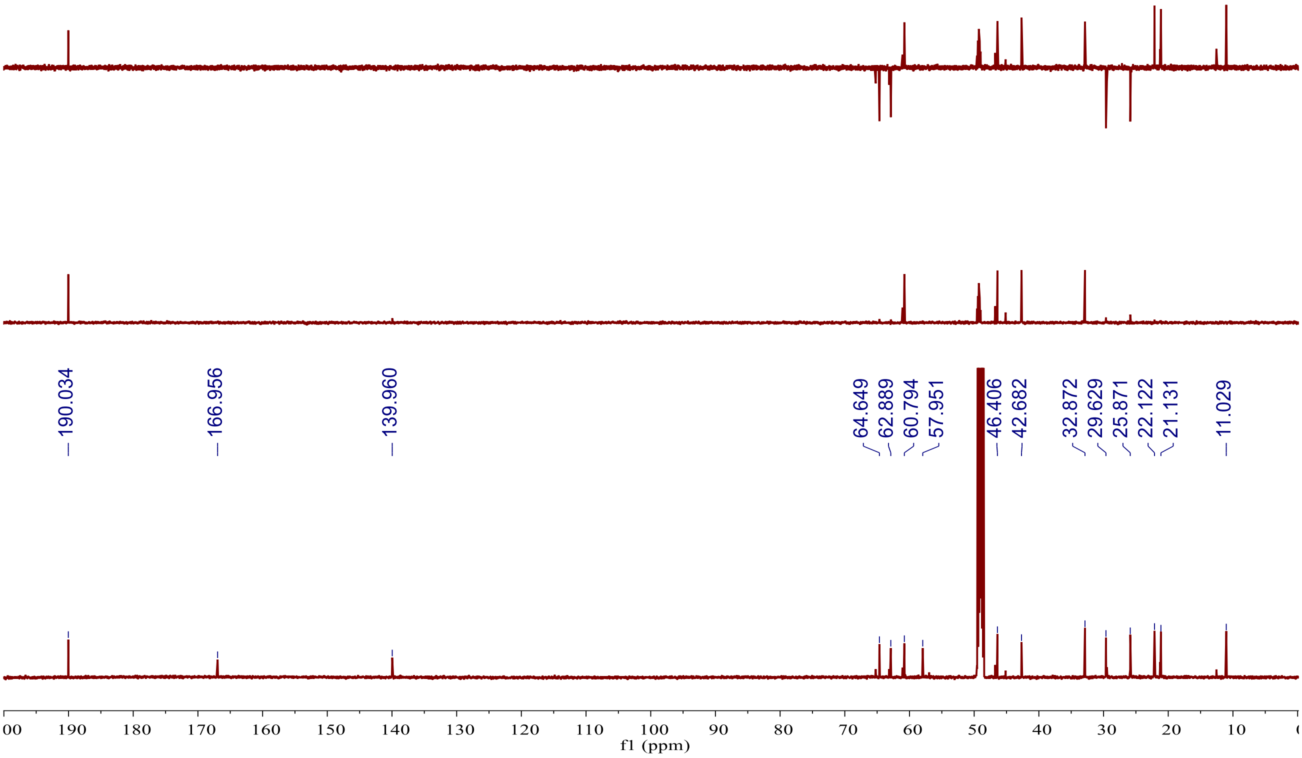

HSQC spectrum

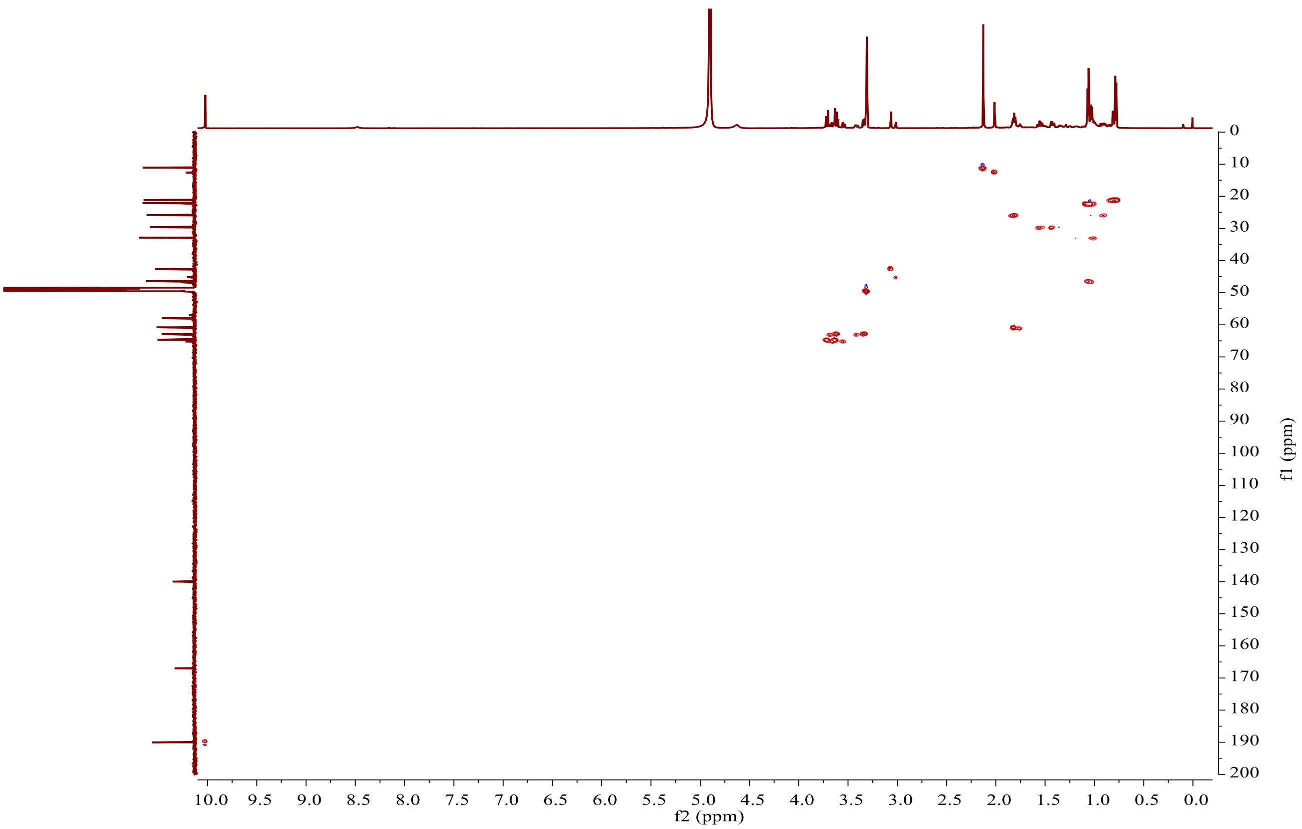

HMBC spectrum

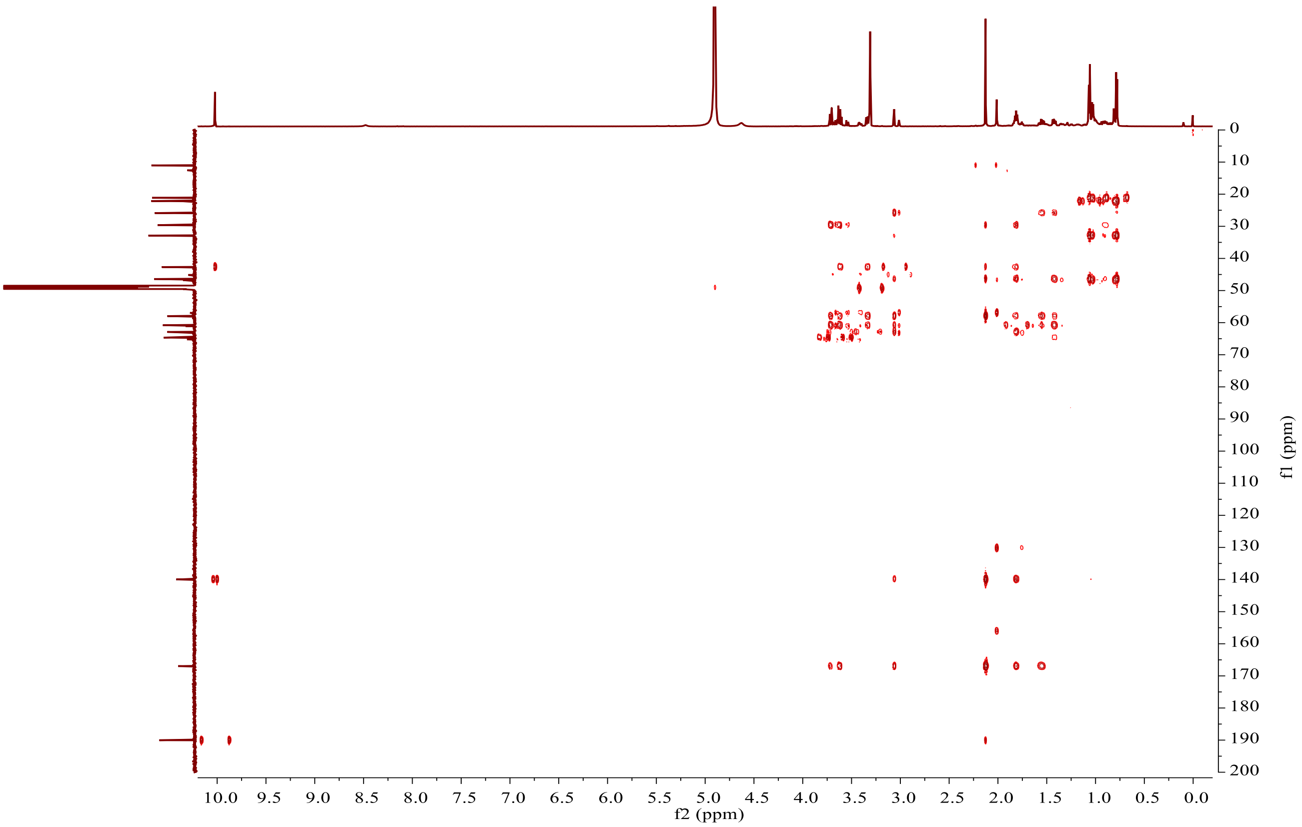

^{1}H-^{1}H COSY spectrum

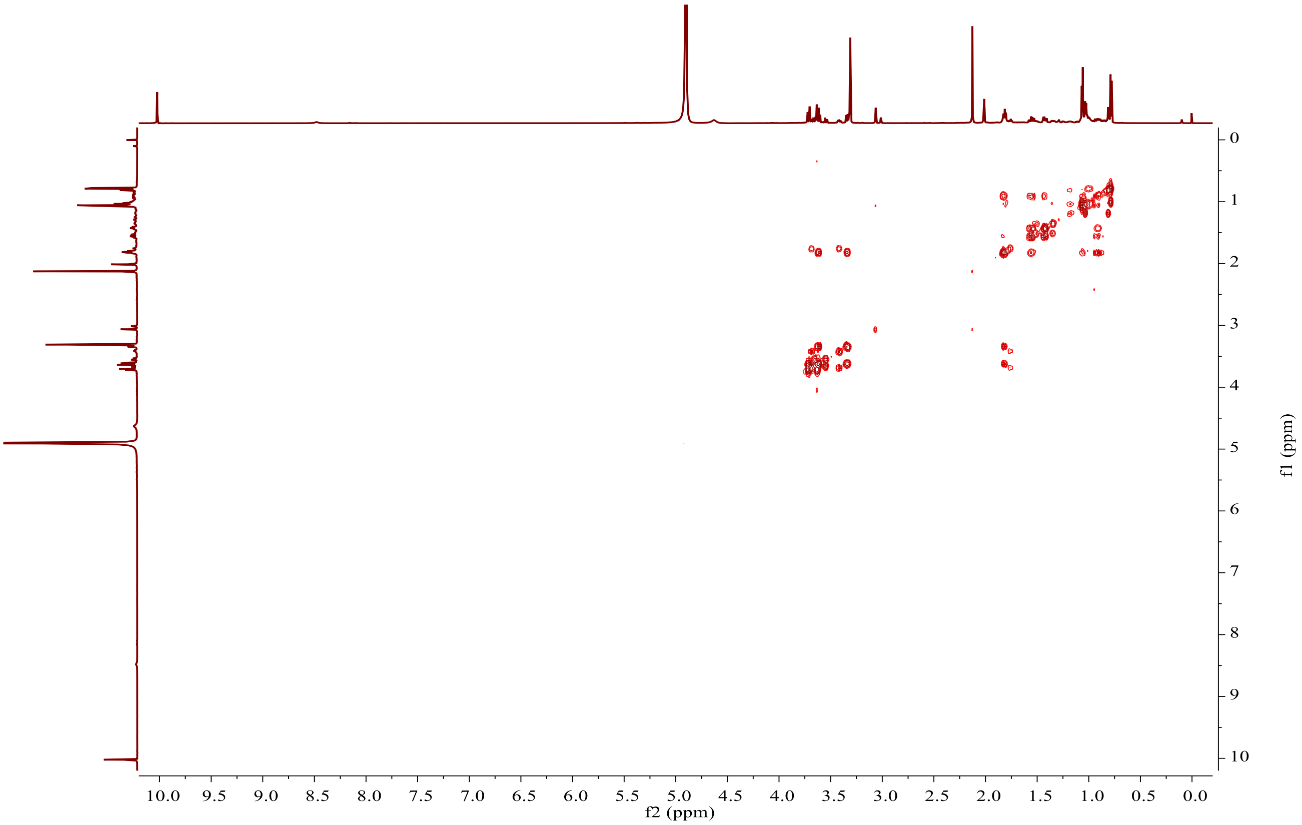

ROESY spectrum

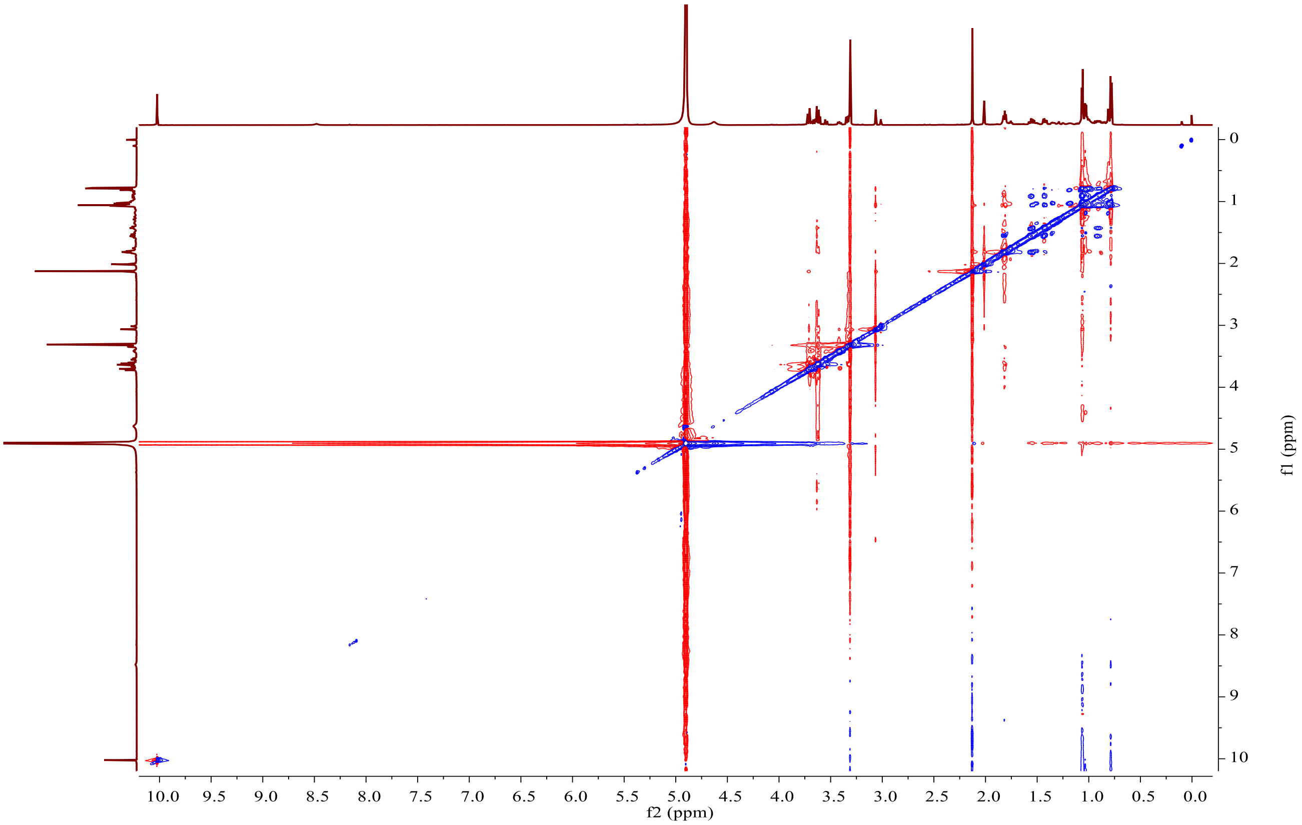

HRESIMS

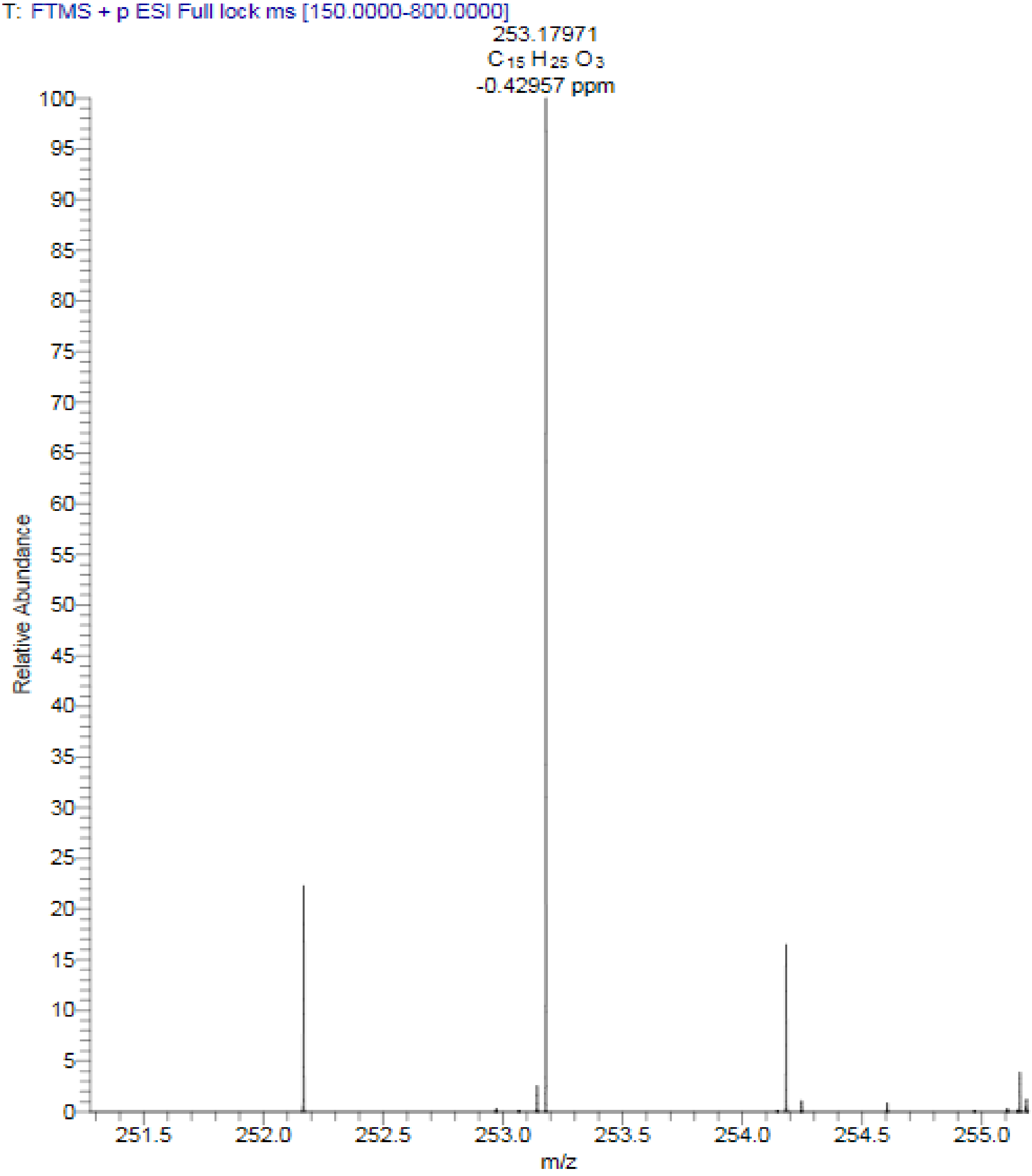

T: FTMS + p ESI Full lock ms [150.0000-800.0000]
253.17971
C 15 H 25 O 3
-0.42957 ppm
Relative Abundance
m/z
100
95
90
85
80
75
70
65
60
55
50
45
40
35
30
25
20
15
10
5
0
251.5
252.0
252.5
253.0
253.5
254.0
254.5
255.0

CD spectra

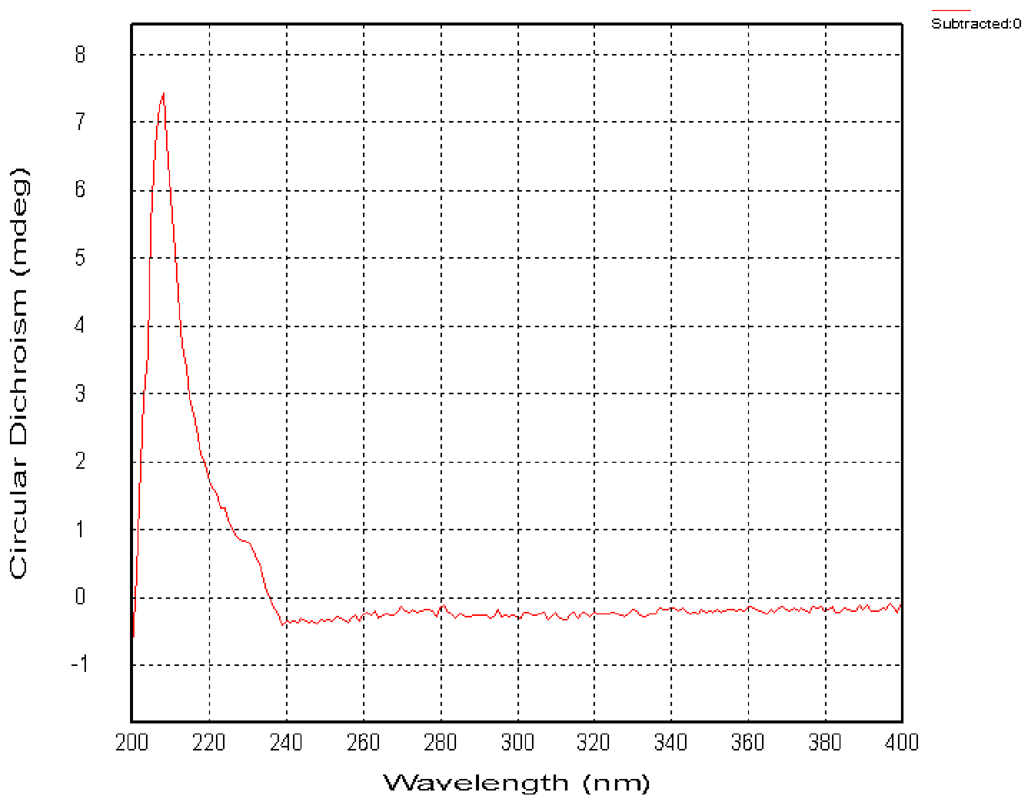

Subtracted:0
Circular Dichroism (mdeg)
8
7
6
5
4
3
2
1
0
-1
200
220
240
260
280
300
320
340
360
380
400
Wavelength (nm)

S1.4 NMR, HRESIMS and CD spectra of bipolarisorokin D (4)

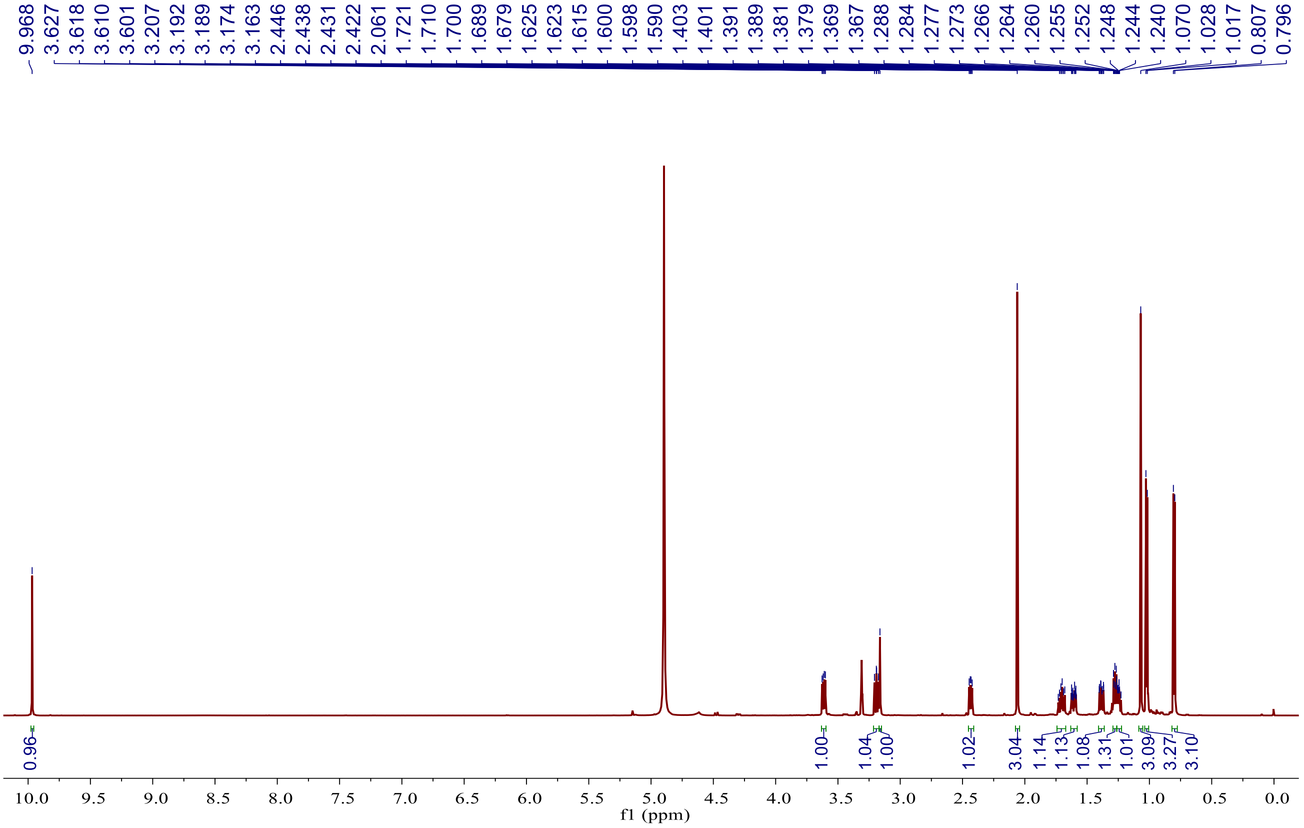

^{13}C NMR and DEPT spectra

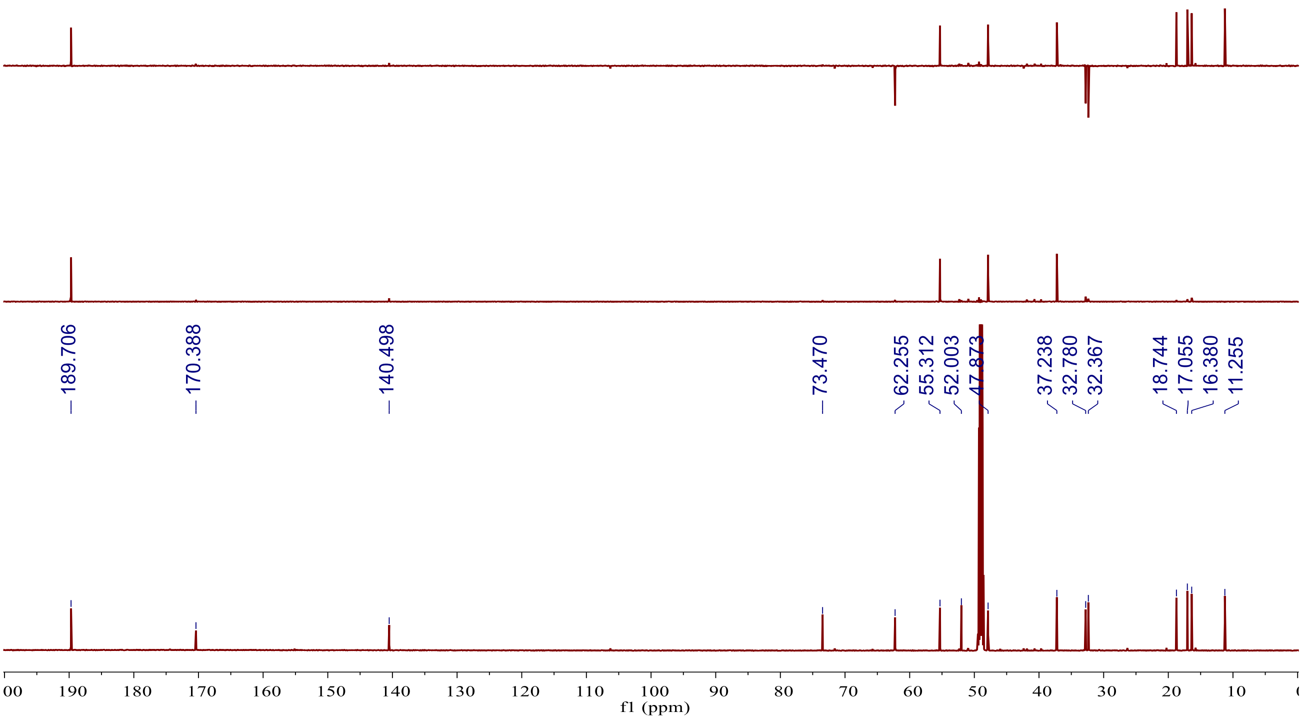

HSQC spectrum

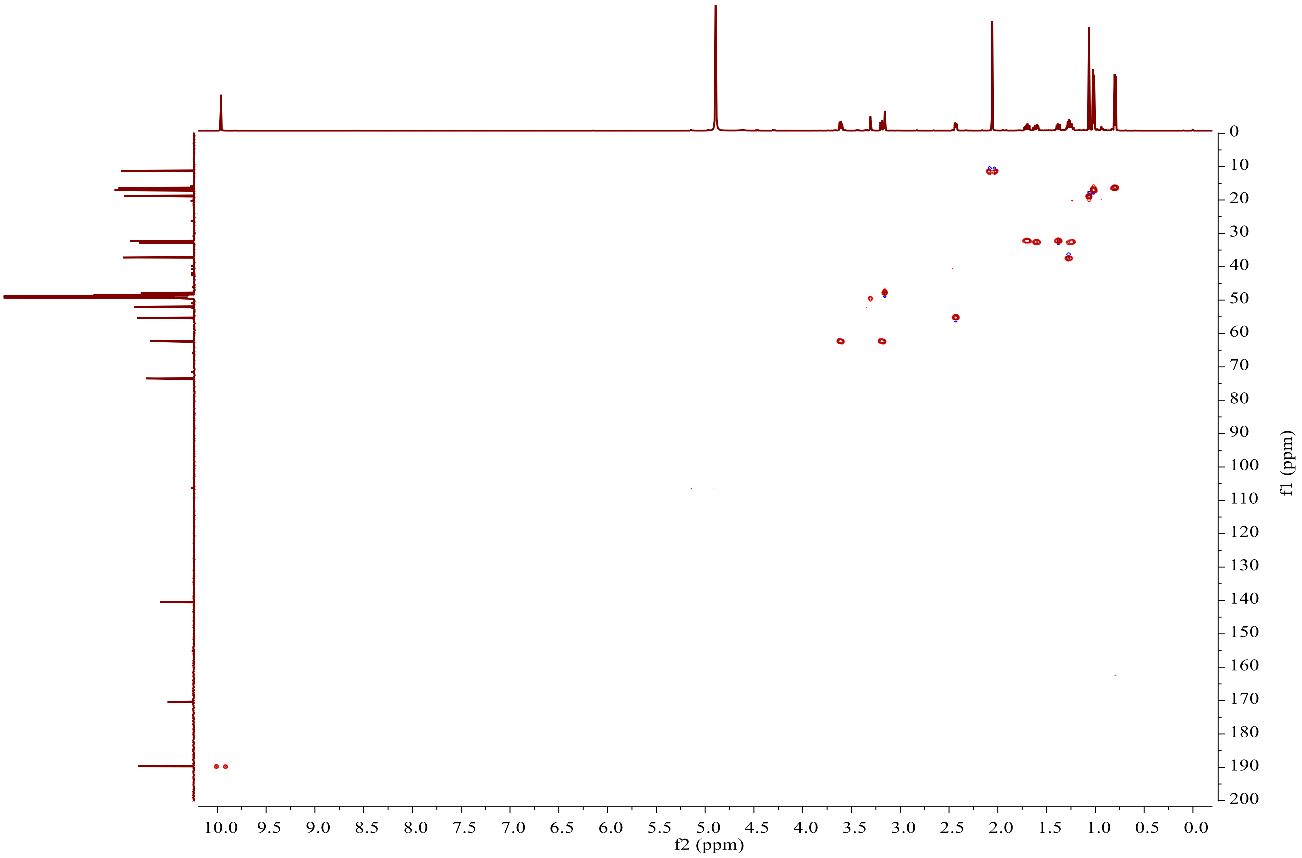

HMBC spectrum

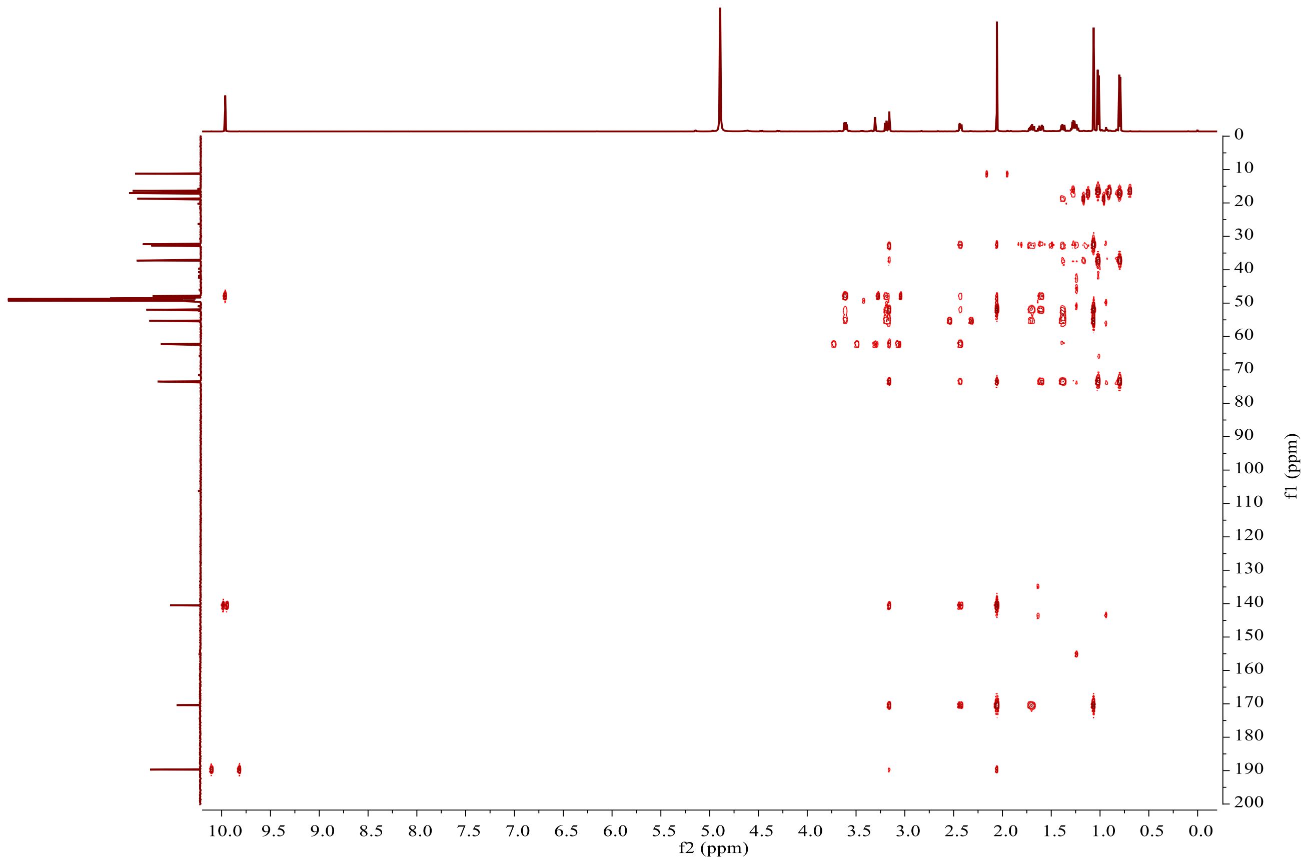

^{1}H-^{1}H COSY spectrum

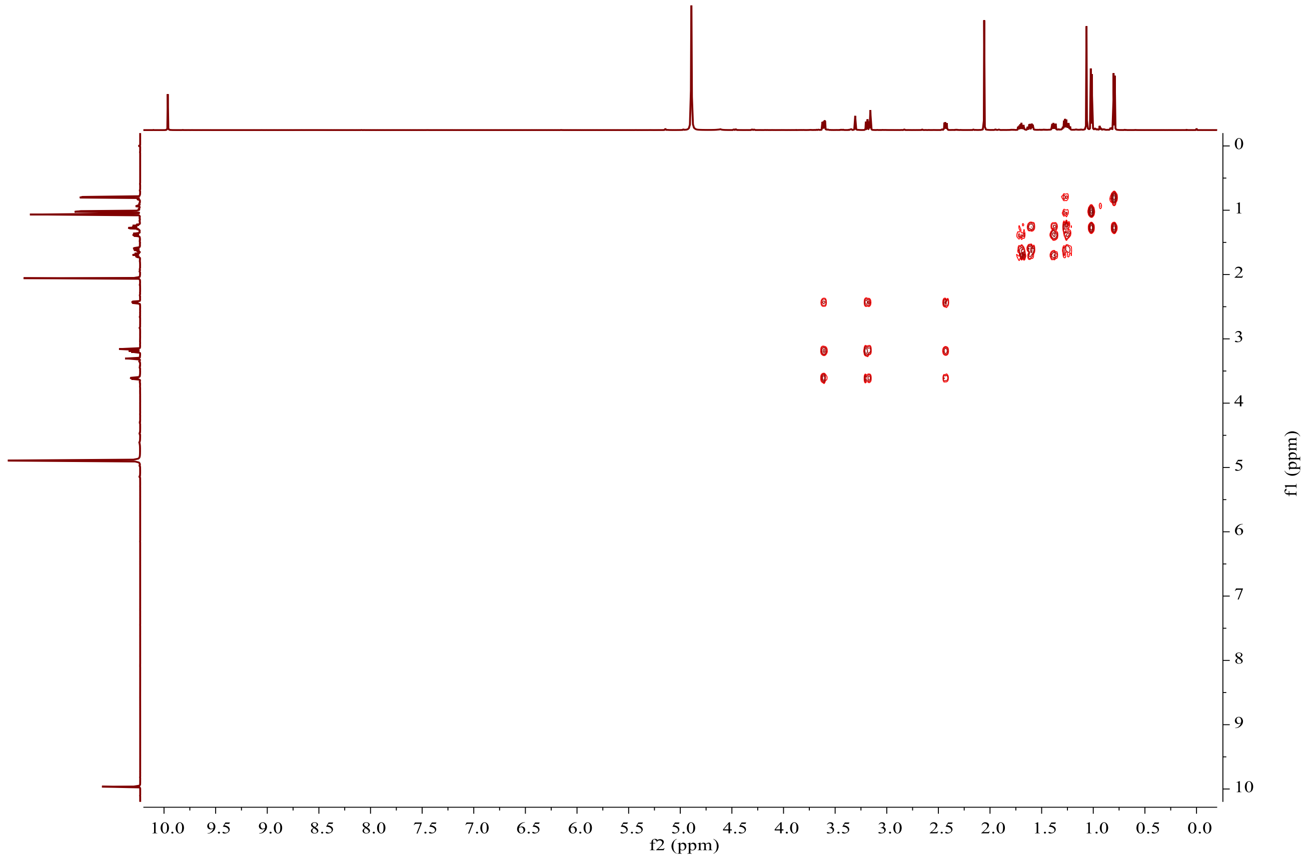

ROESY spectrum

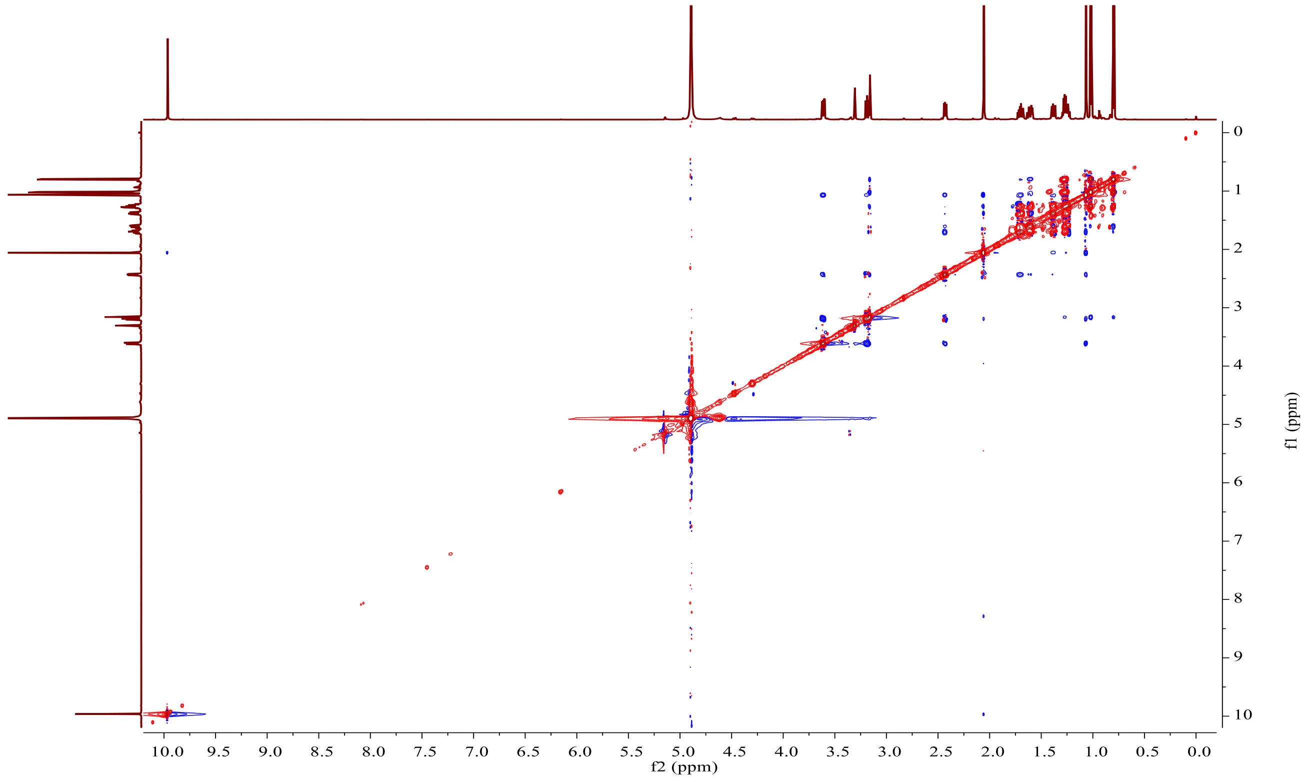

HRESIMS

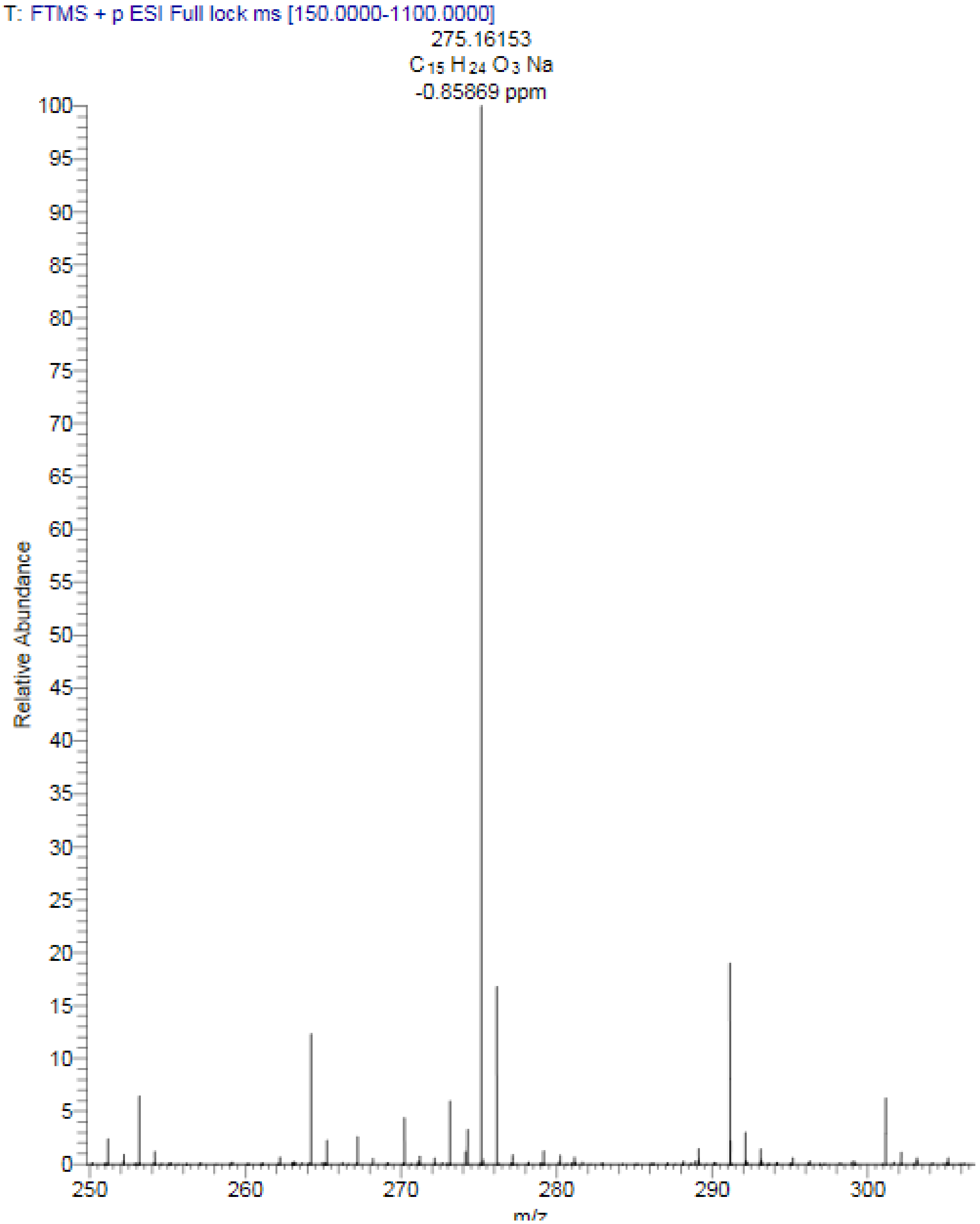

T: FTMS + p ESI Full lock ms [150.0000-1100.0000]
275.16153
C 15 H 24 O 3 Na
-0.85869 ppm
Relative Abundance
m/z

CD spectra

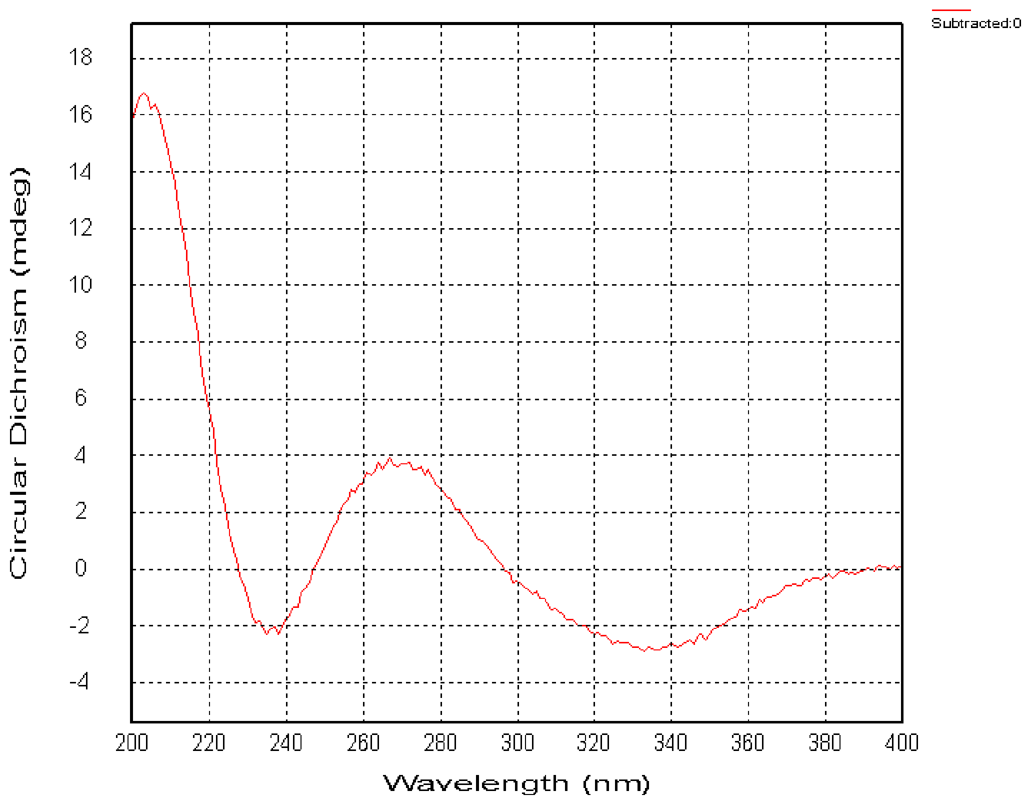

Subtracted:0
Circular Dichroism (mdeg)
18
16
14
12
10
8
6
4
2
0
-2
-4
200
220
240
260
280
300
320
340
360
380
400
Wavelength (nm)

S1.5 NMR, HRESIMS and CD spectra of bipolarisorokin E (5)

^{1}H NMR spectrum

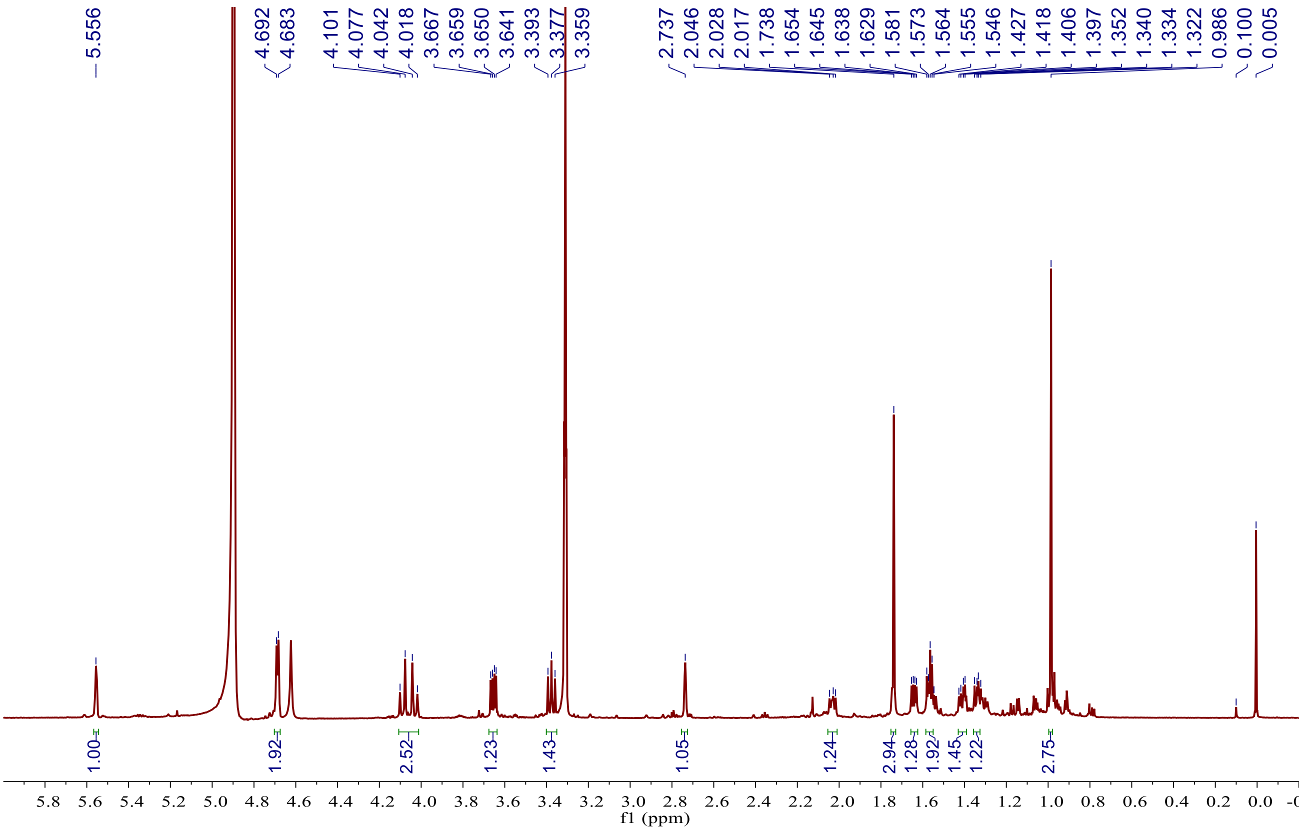

^{13}C NMR and DEPT spectra

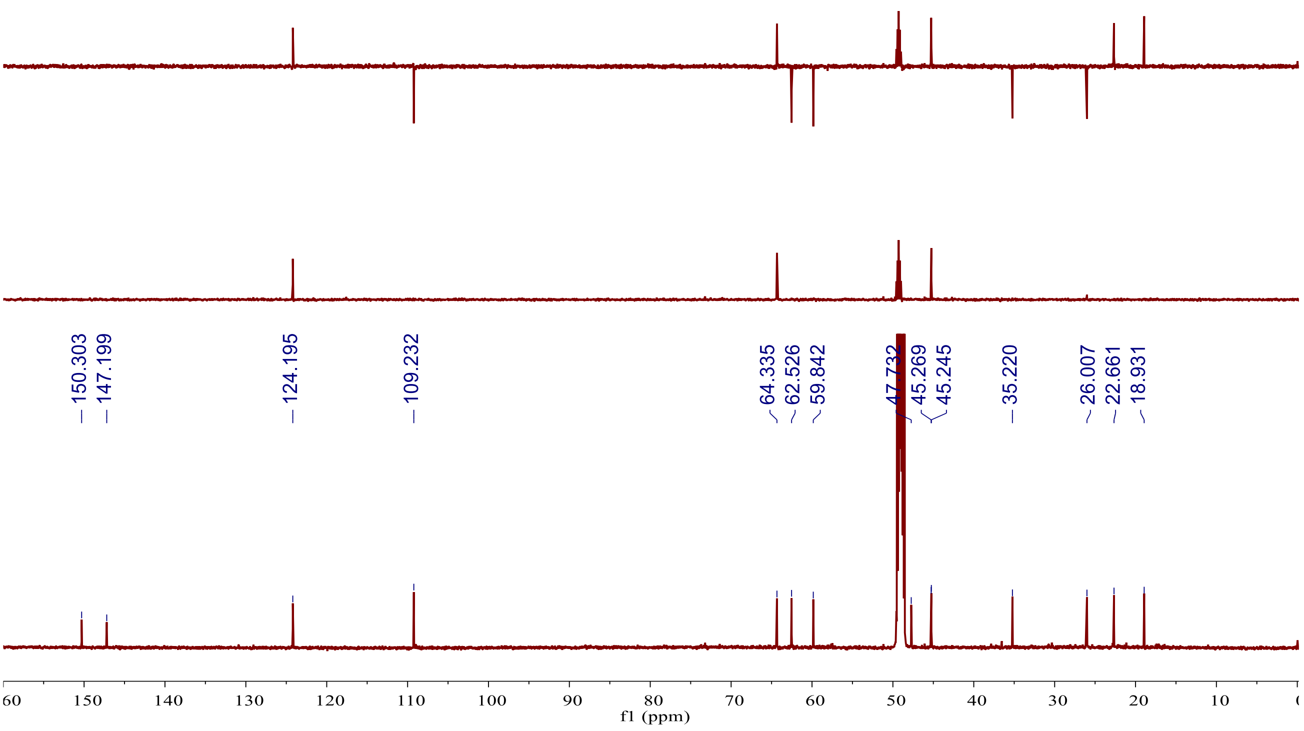

HSQC spectrum

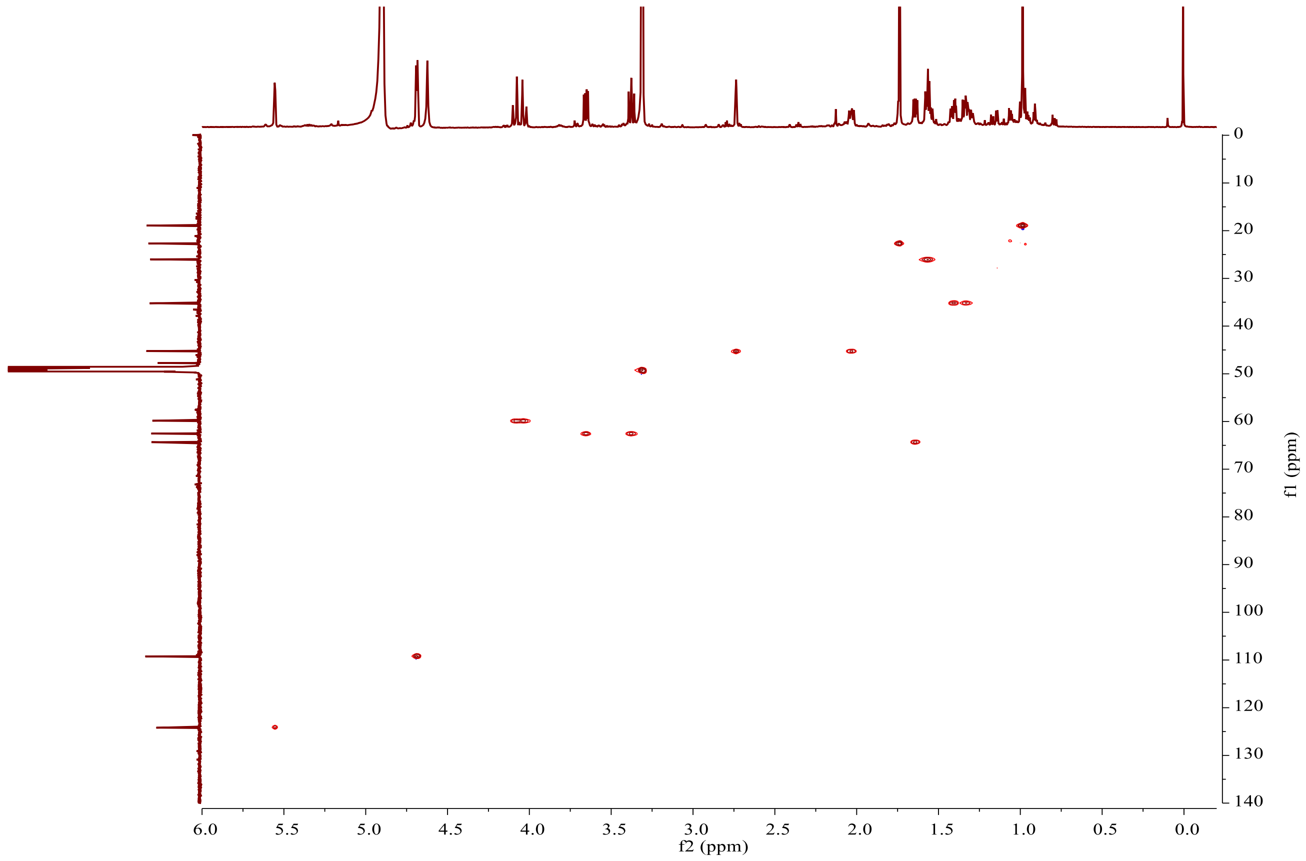

HMBC spectrum

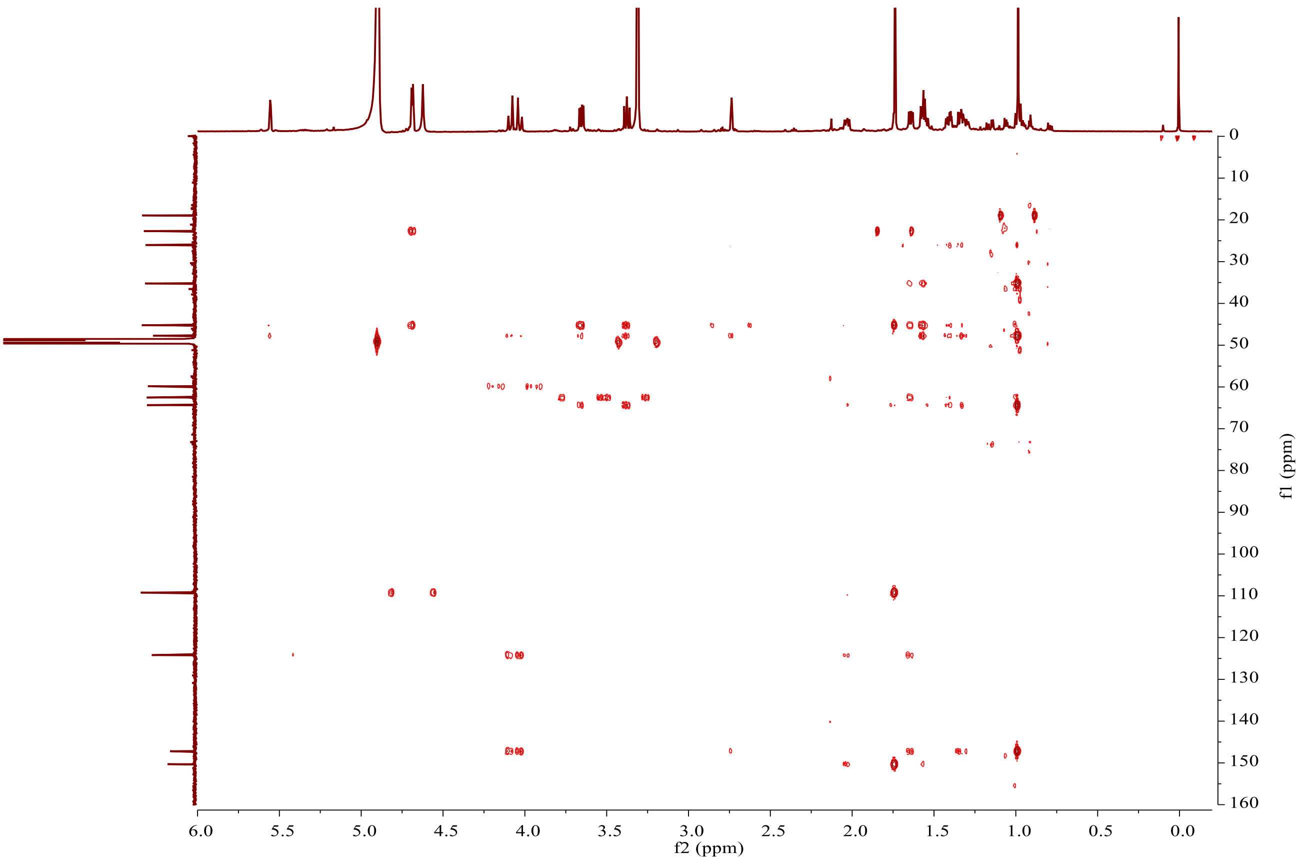

^{1}H-^{1}H COSY spectrum

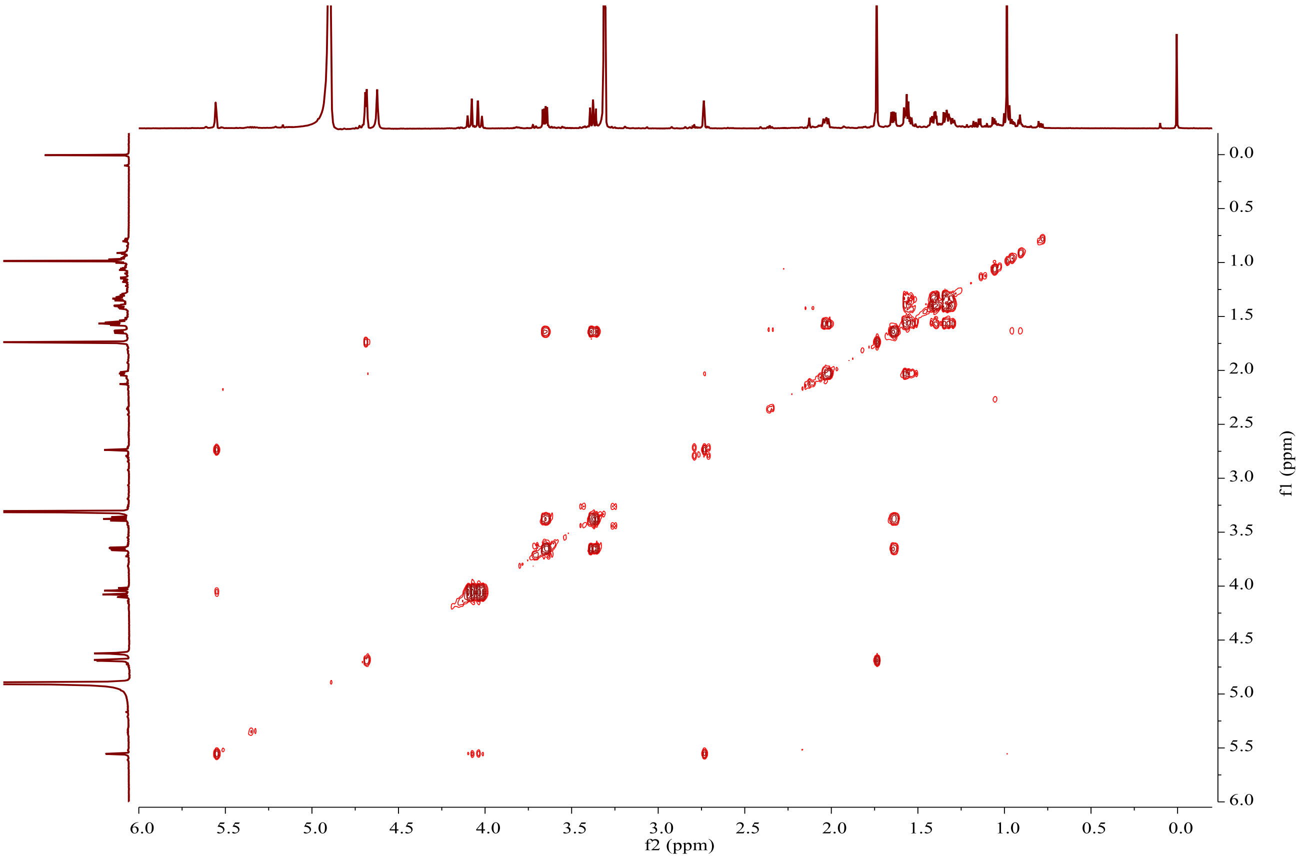

ROESY spectrum

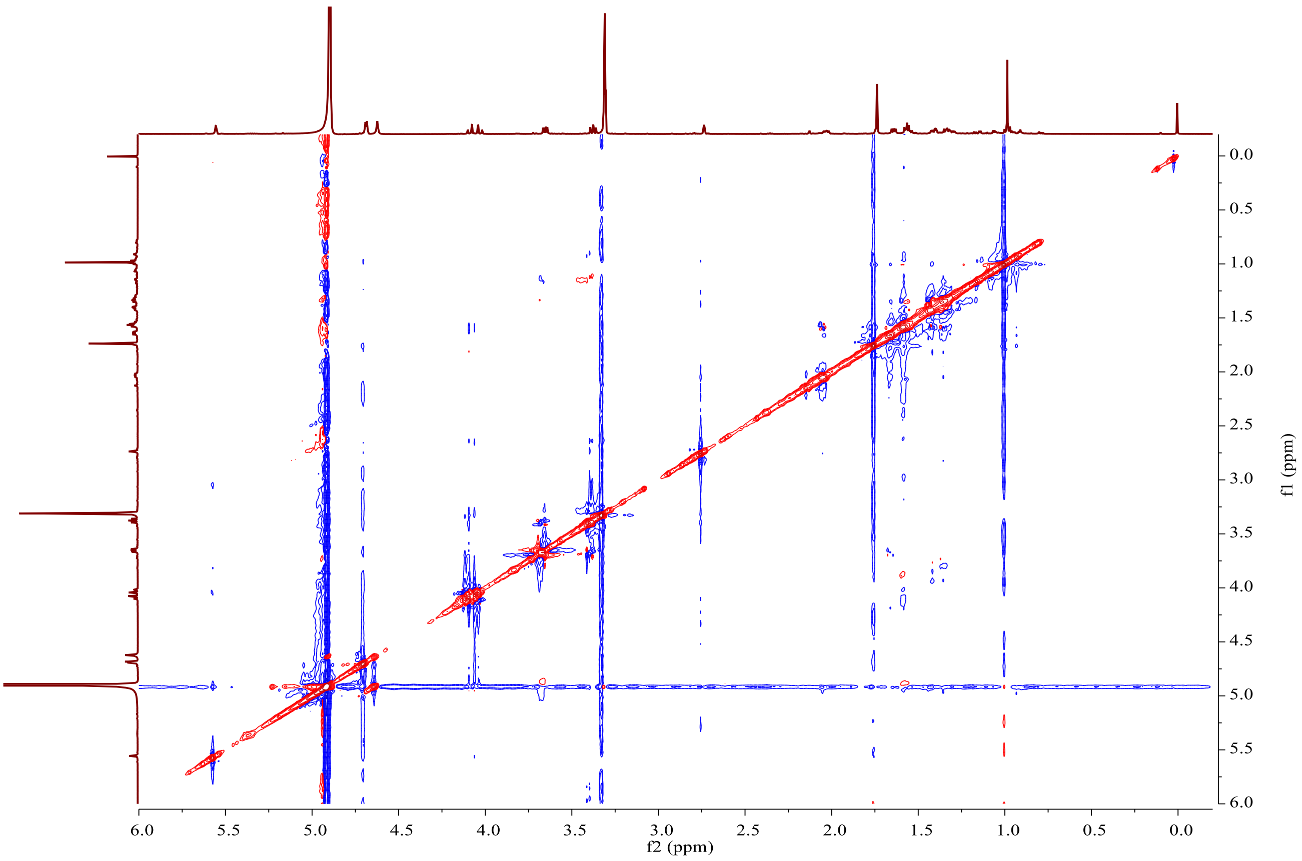

HRESIMS

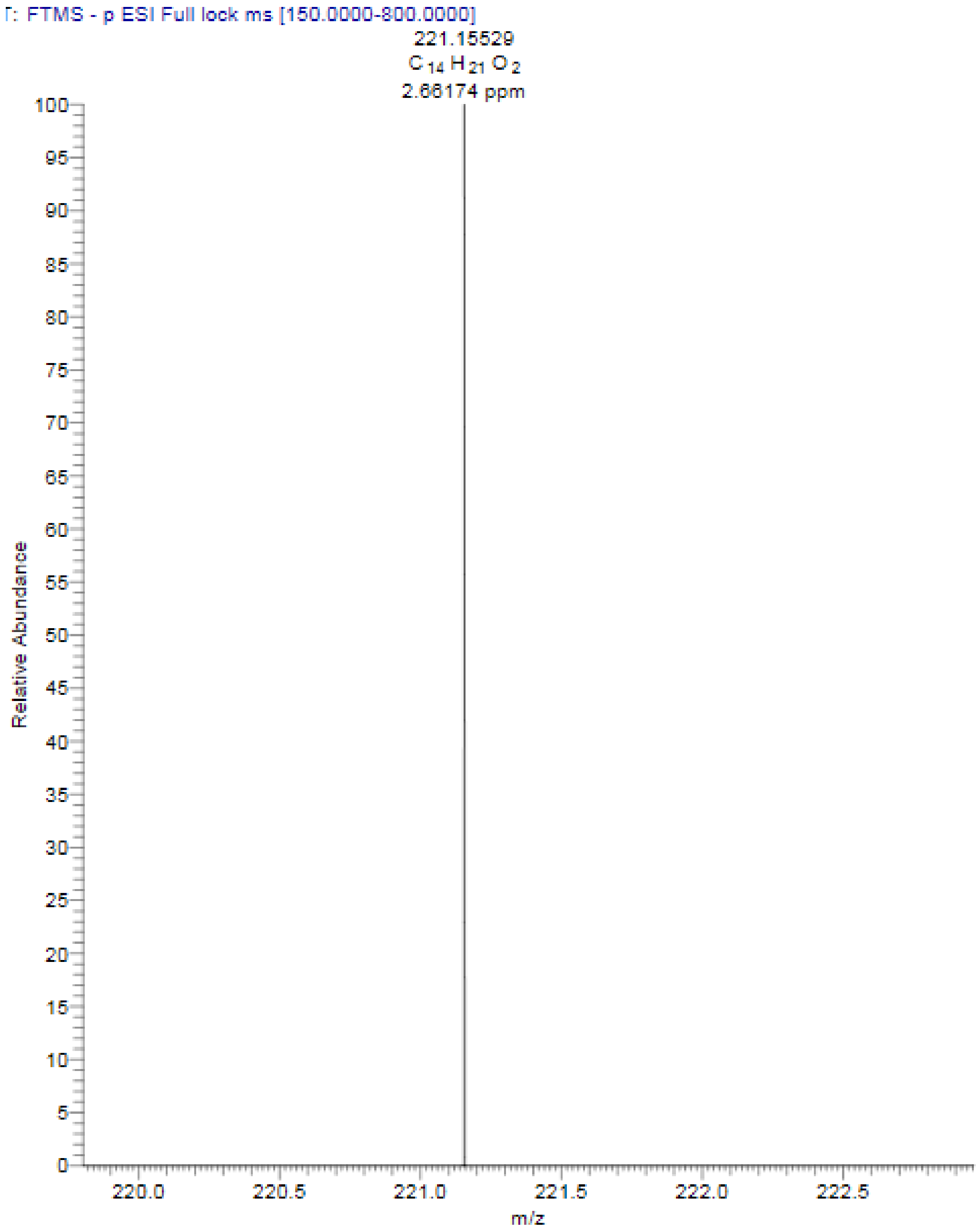
T: FTMS - p ESI Full lock ms [150.0000-800.0000]
221.15529
C 14 H 21 O 2
2.66174 ppm
Relative Abundance
m/z

CD spectra

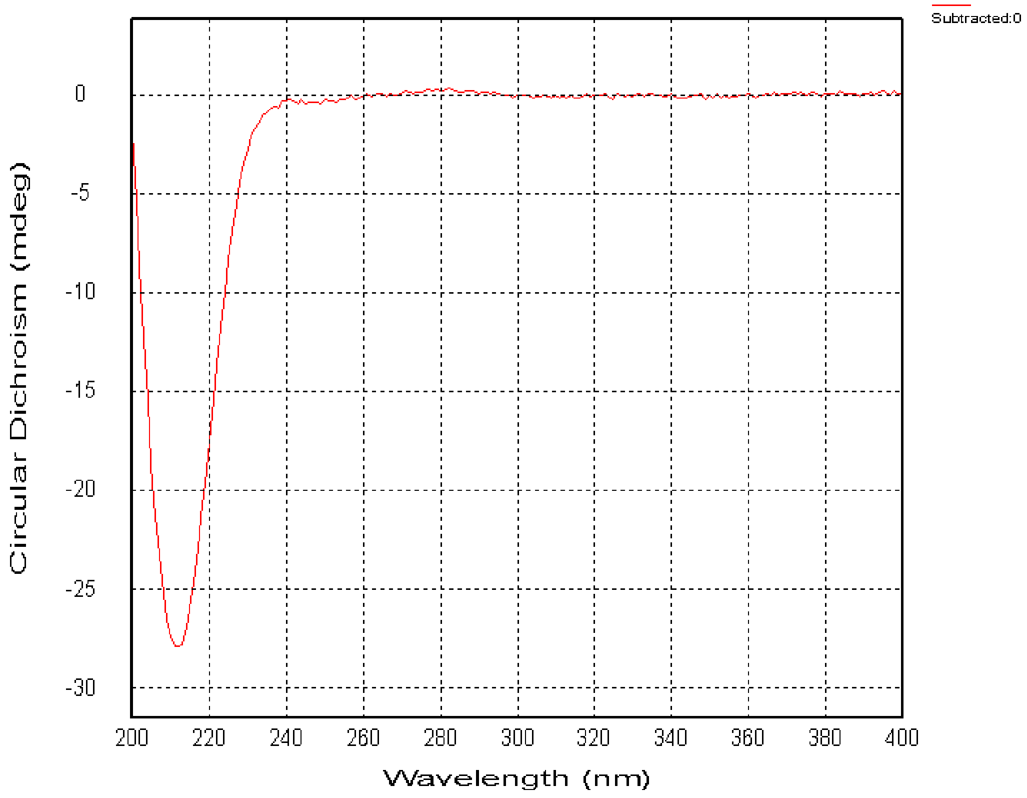
Subtracted:0
0
-5
-10
-15
-20
-25
-30
200
220
240
260
280
300
320
340
360
380
400
Circular Dichroism (mdeg)
Wavelength (nm)

S1.6 NMR and HRESIMS spectra of bipolarisorokin F (6)

^{1}H NMR spectrum

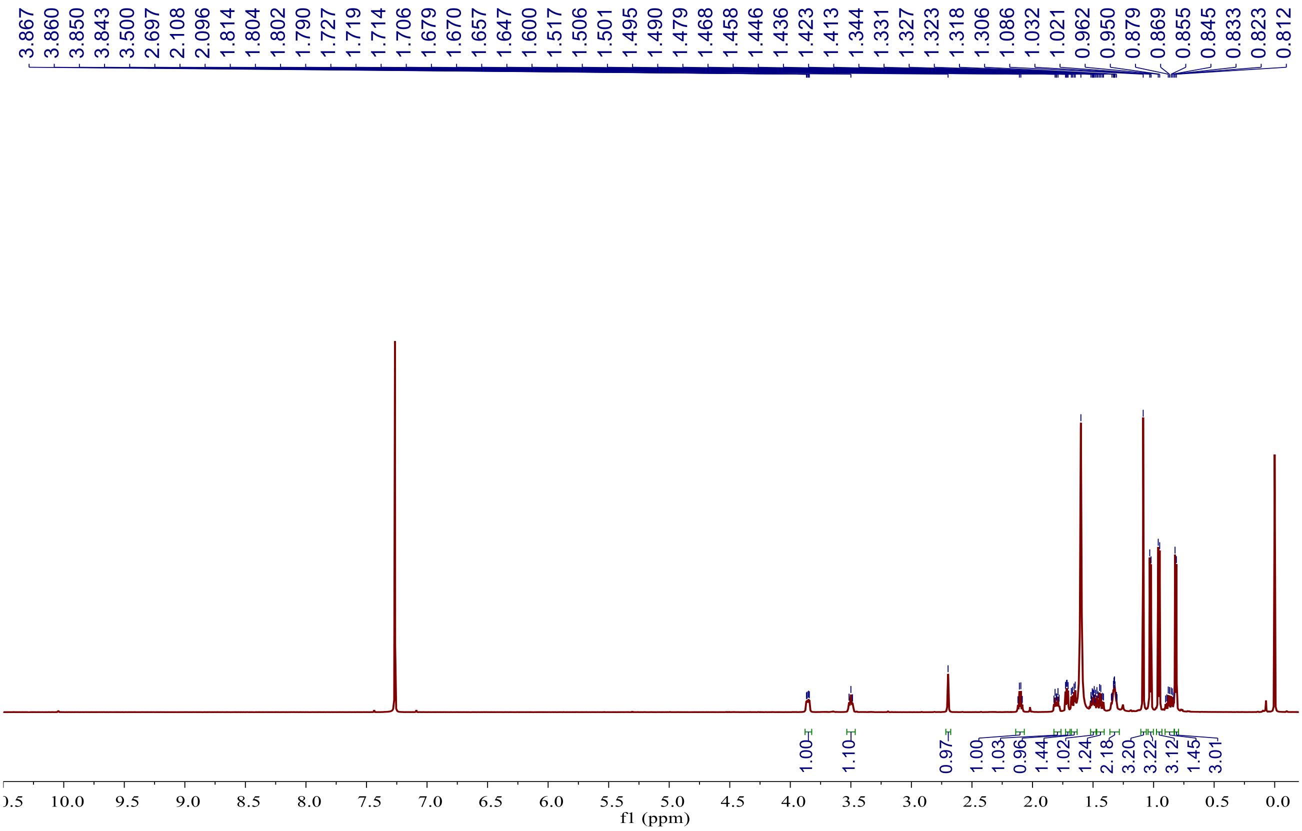

^{13}C NMR and DEPT spectra

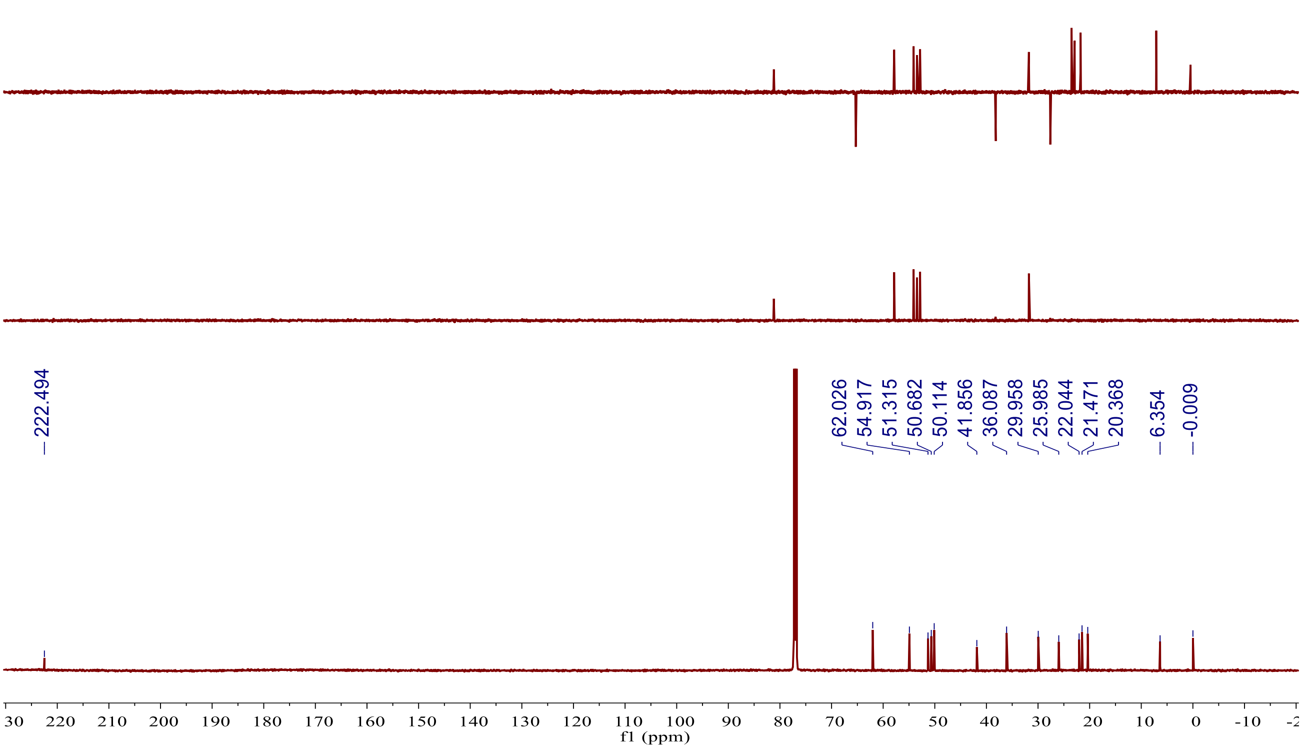

HSQC spectrum

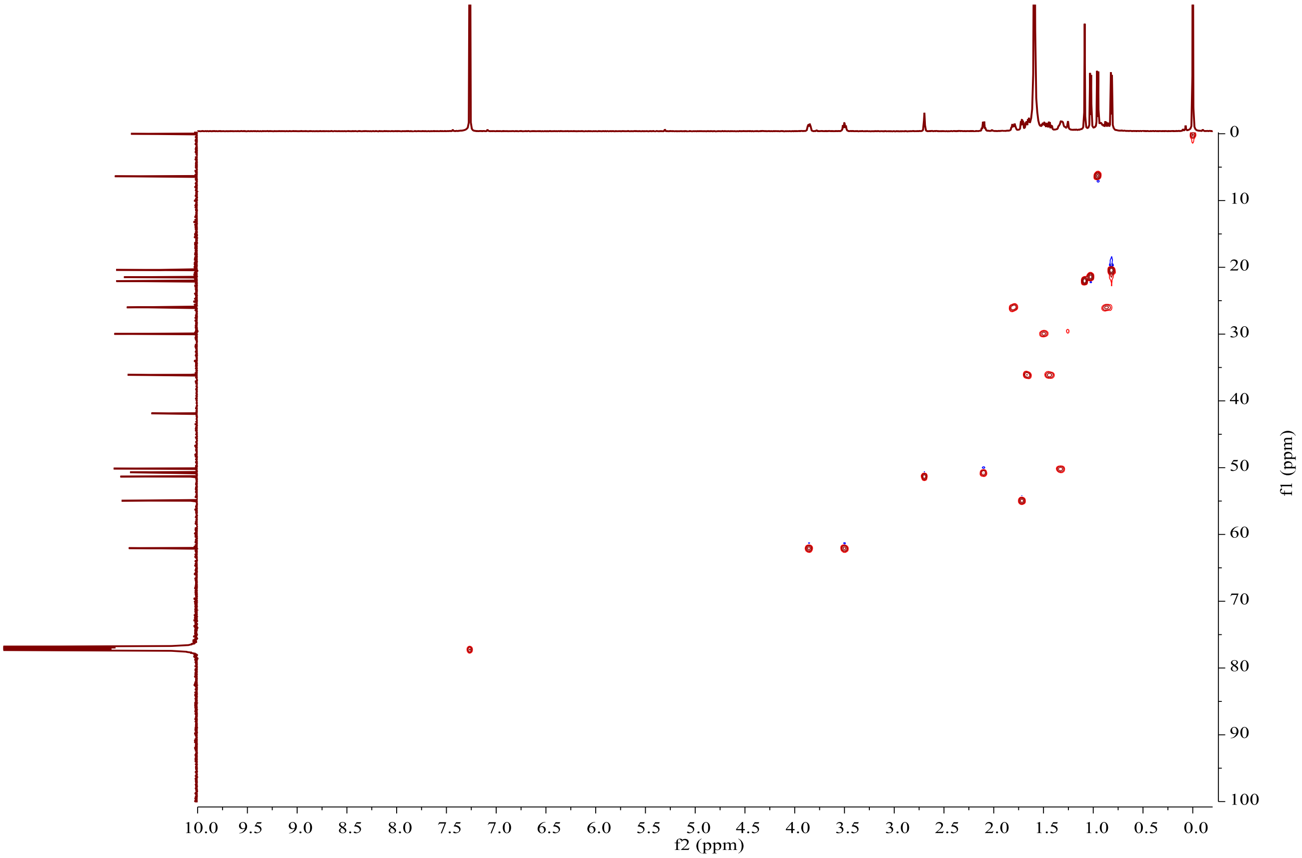

HMBC spectrum

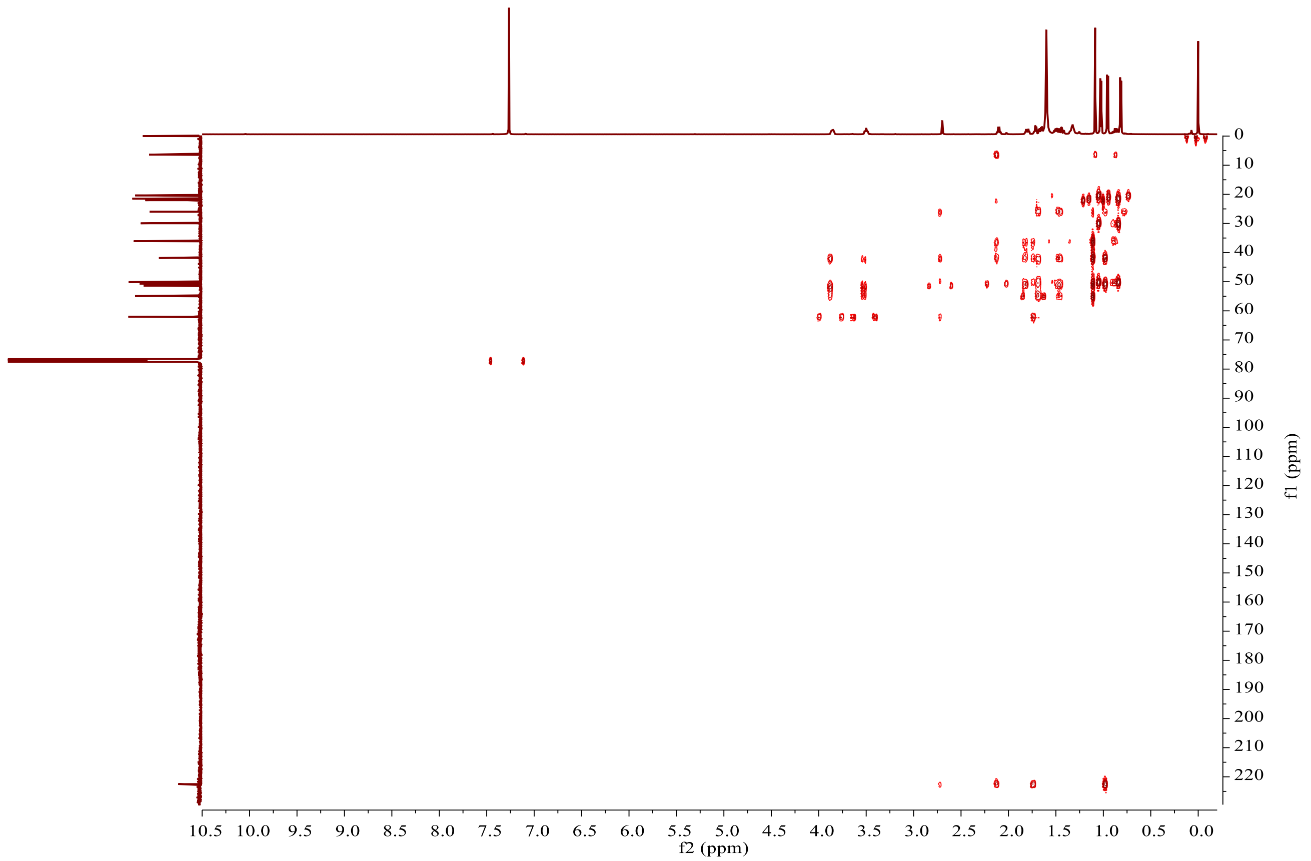

^{1}H-^{1}H COSY spectrum

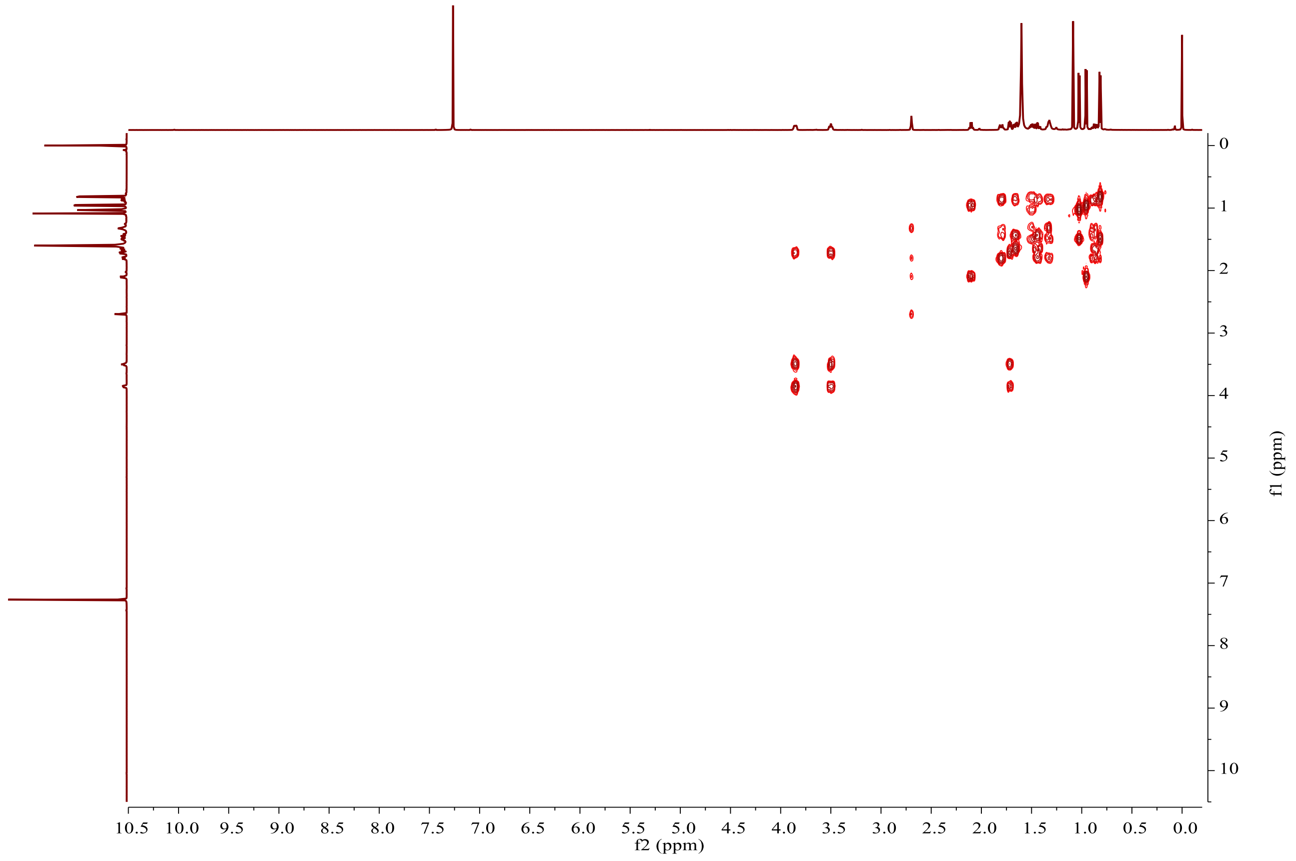

ROESY spectrum

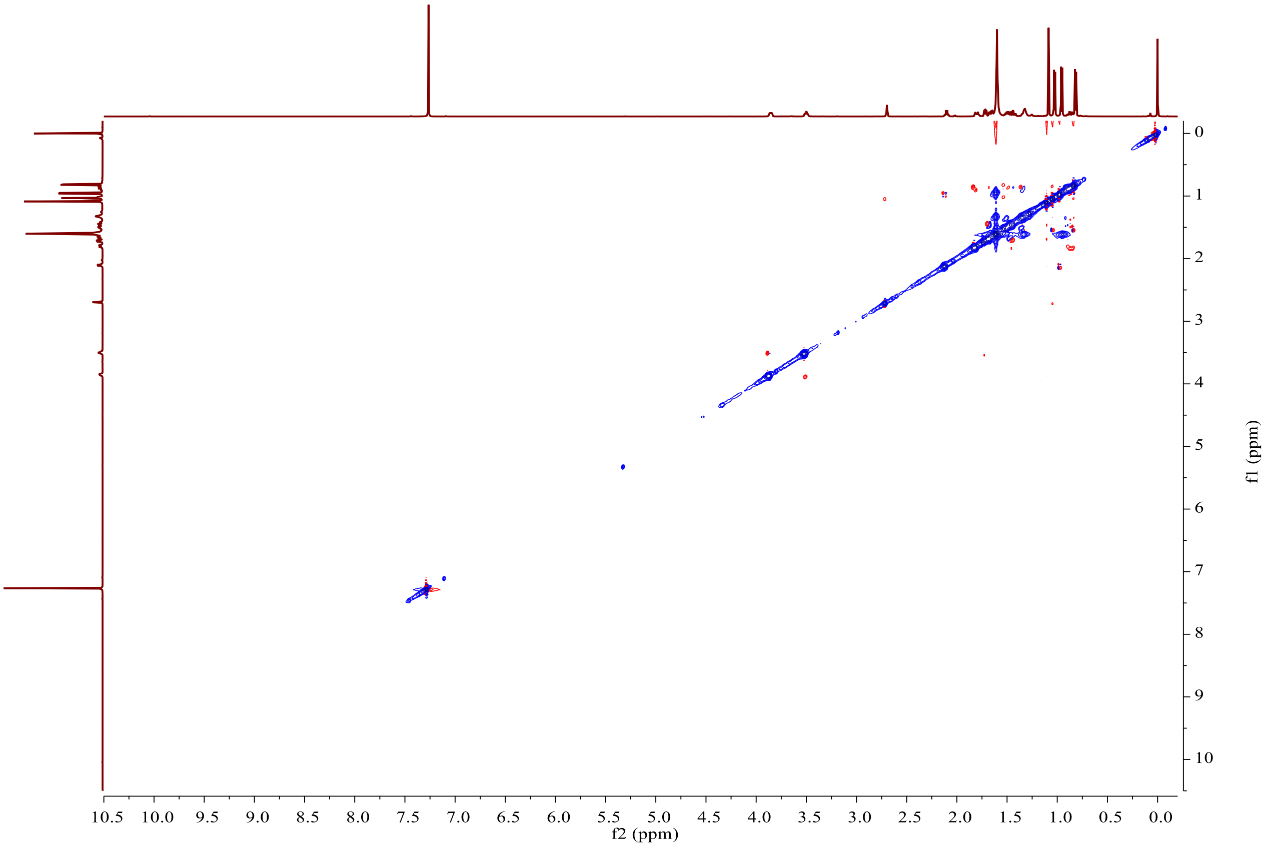

HRESIMS

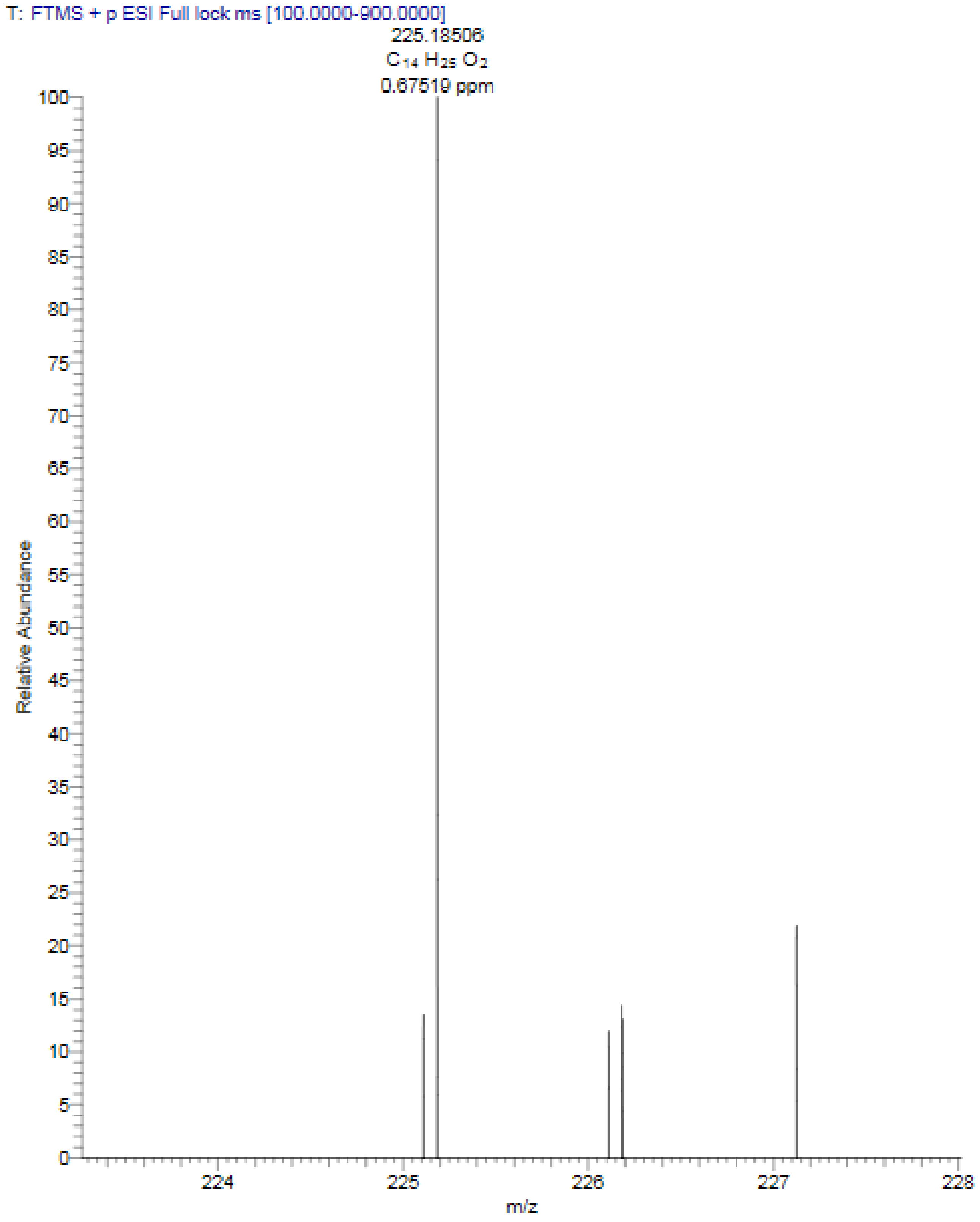

T: FTMS + p ESI Full lock ms [100.0000-900.0000]
225.18506
C 14 H25 O2
0.67519 ppm
Relative Abundance
m/z

S1.7 NMR, HRESIMS and CD spectra of bipolarisorokin G (7)

^{1}H NMR spectrum

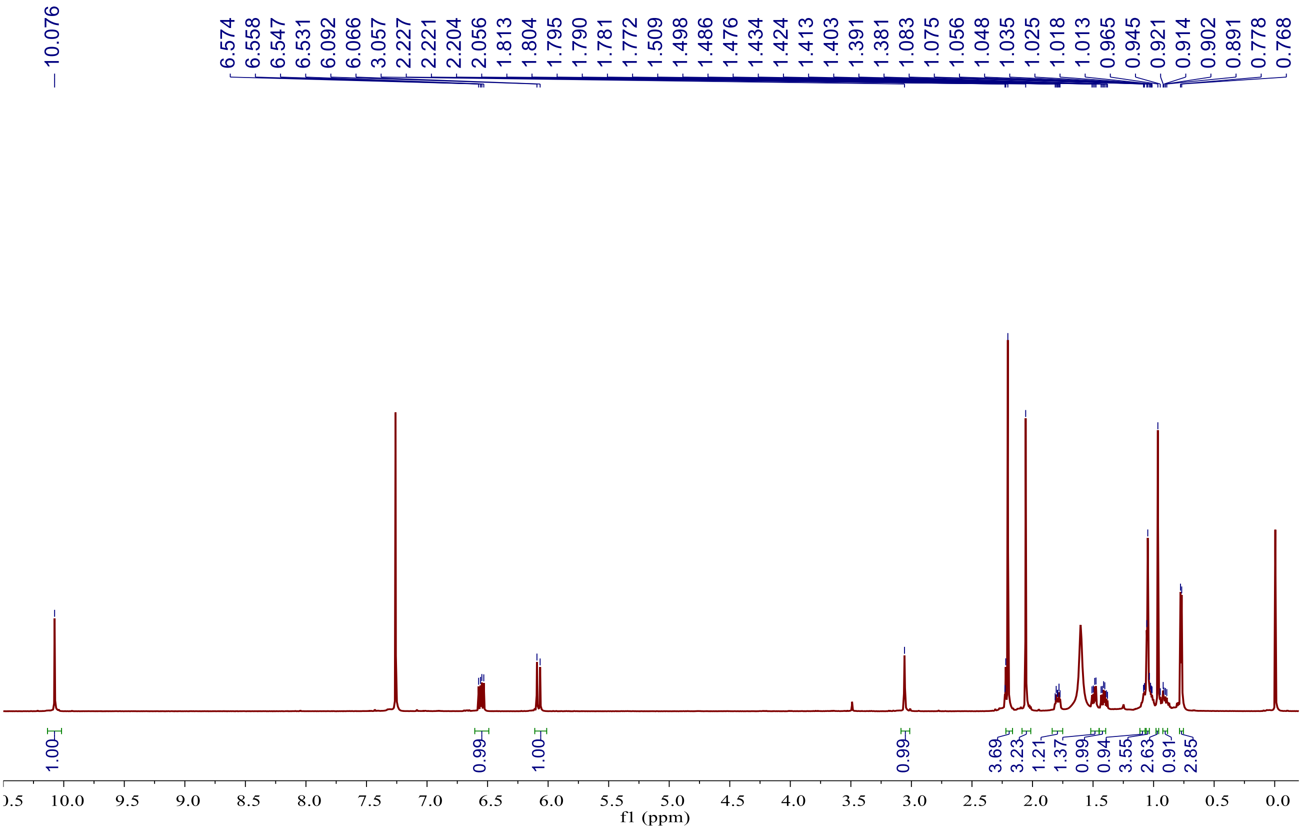

^{13}C NMR and DEPT spectra

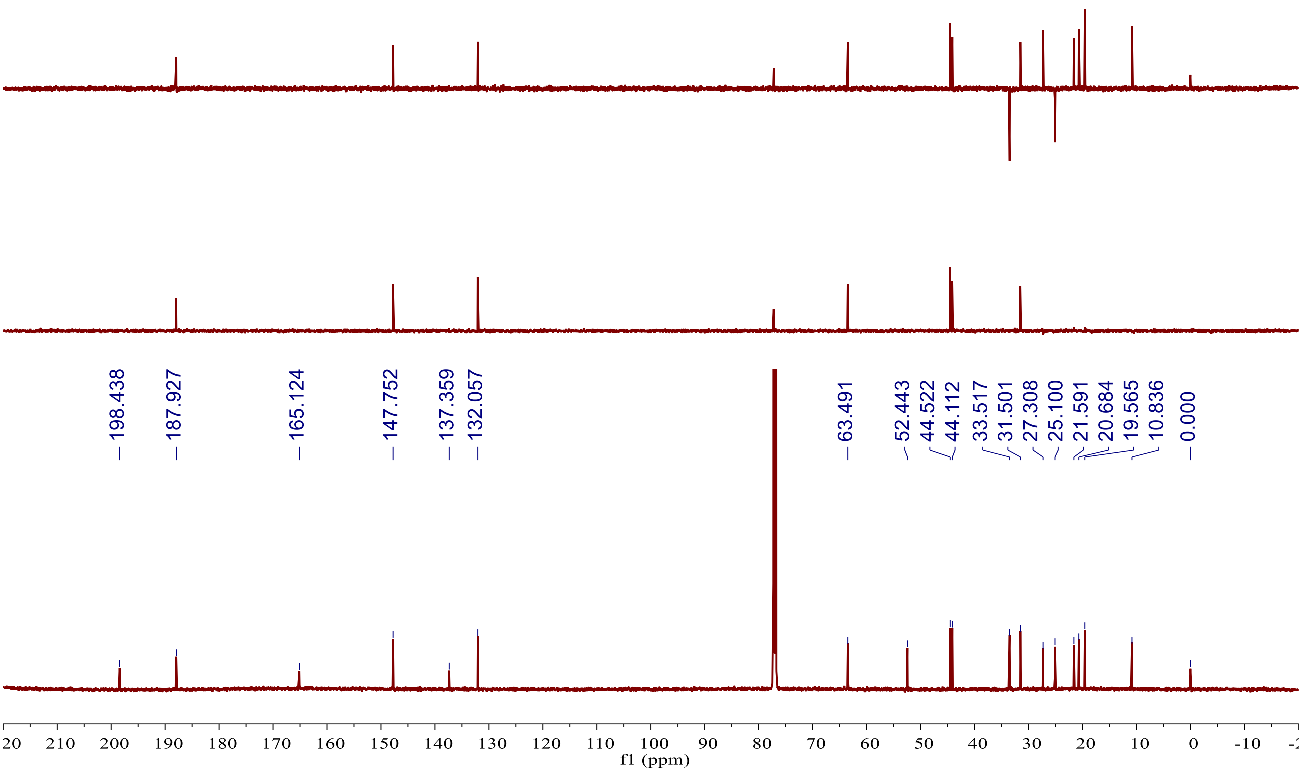

HSQC spectrum

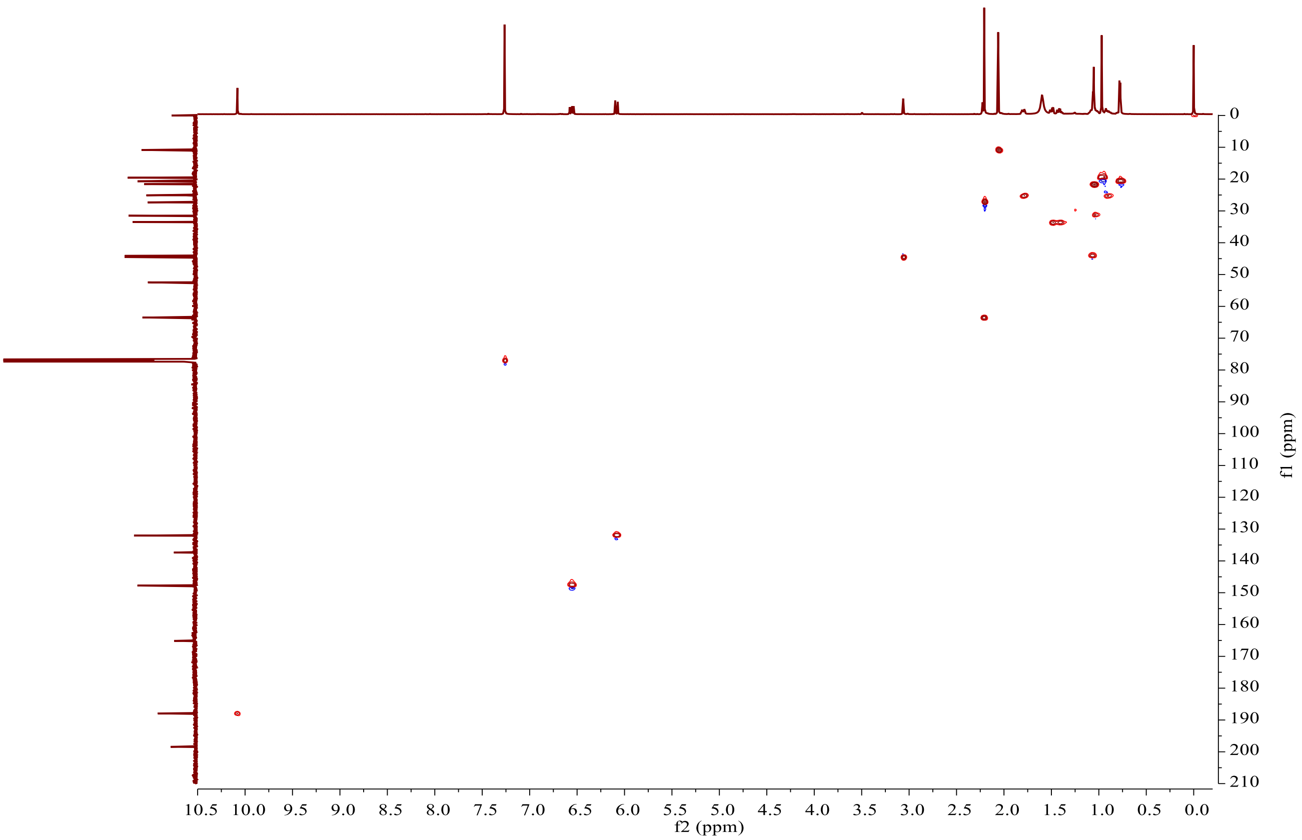

HMBC spectrum

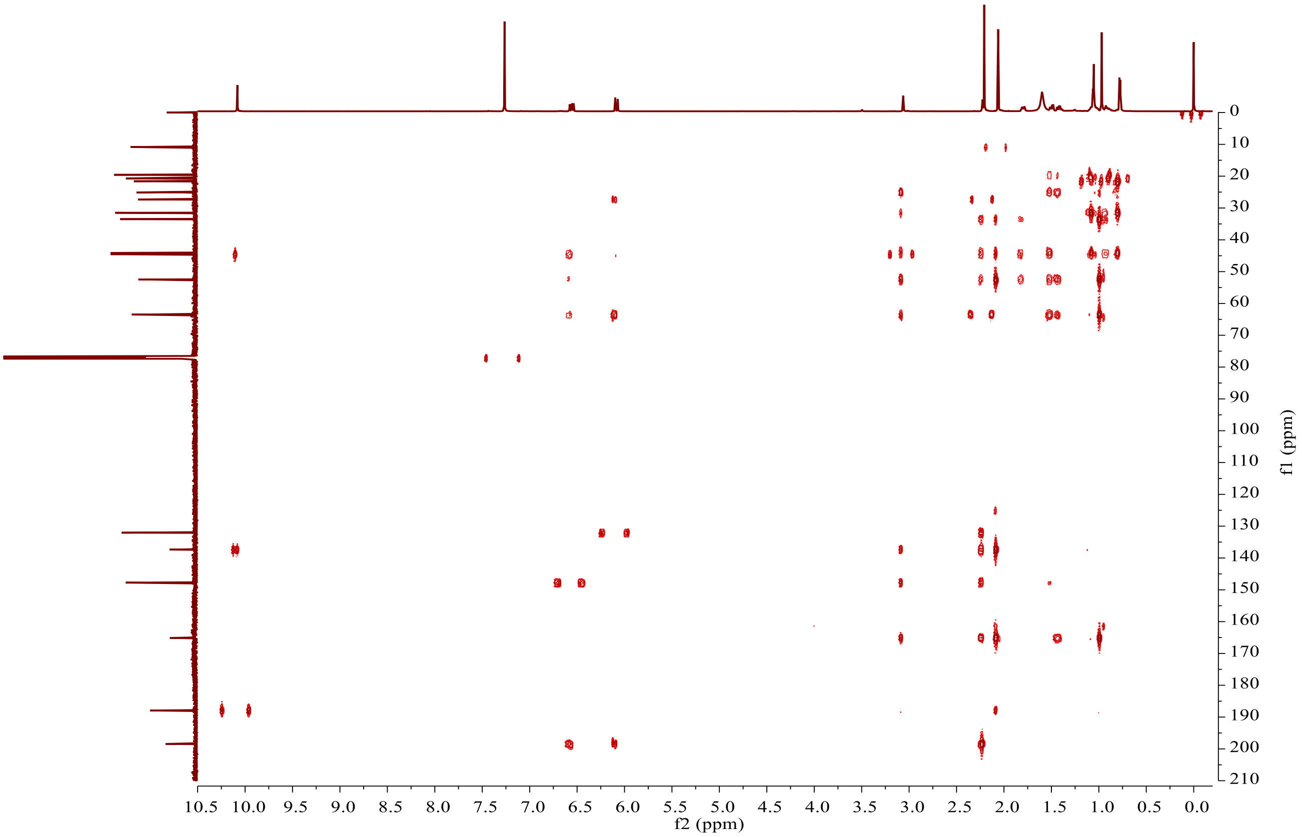

^{1}H-^{1}H COSY spectrum

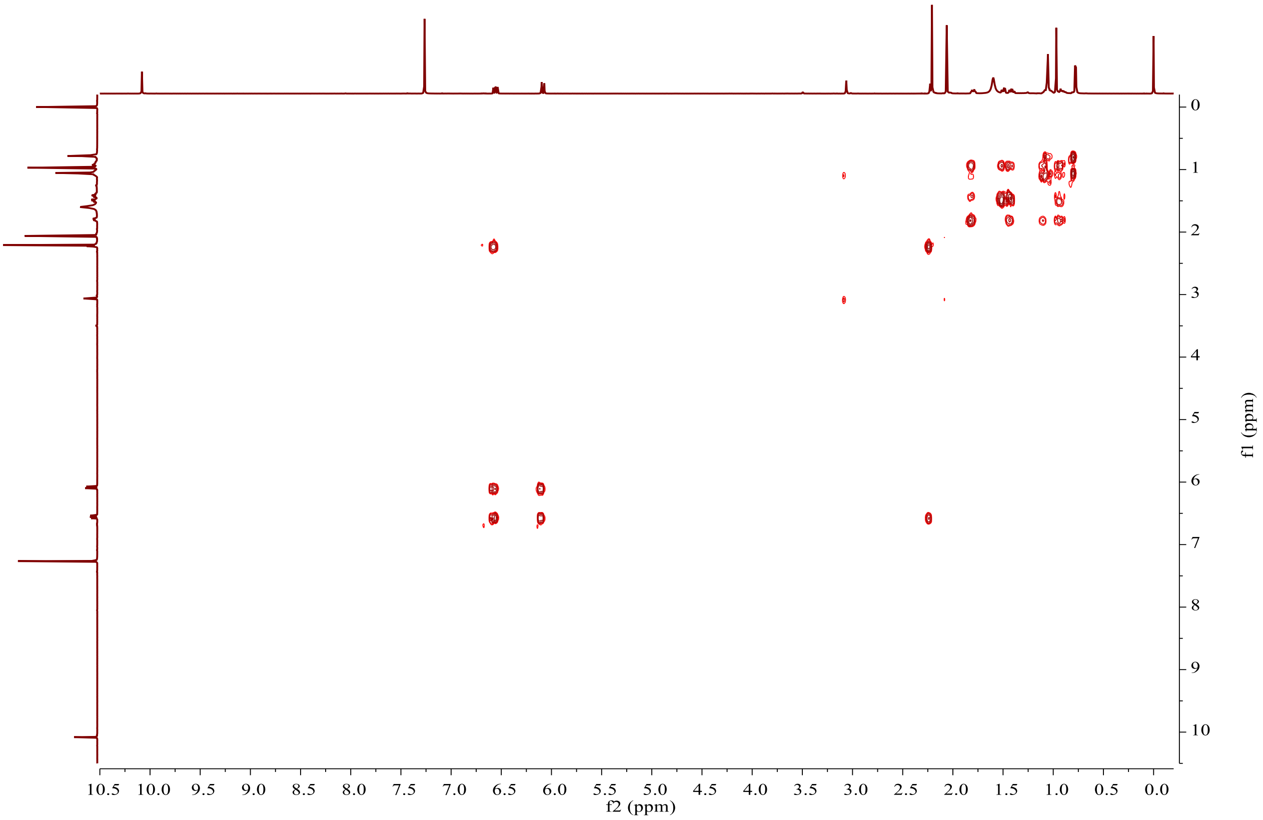

ROESY spectrum

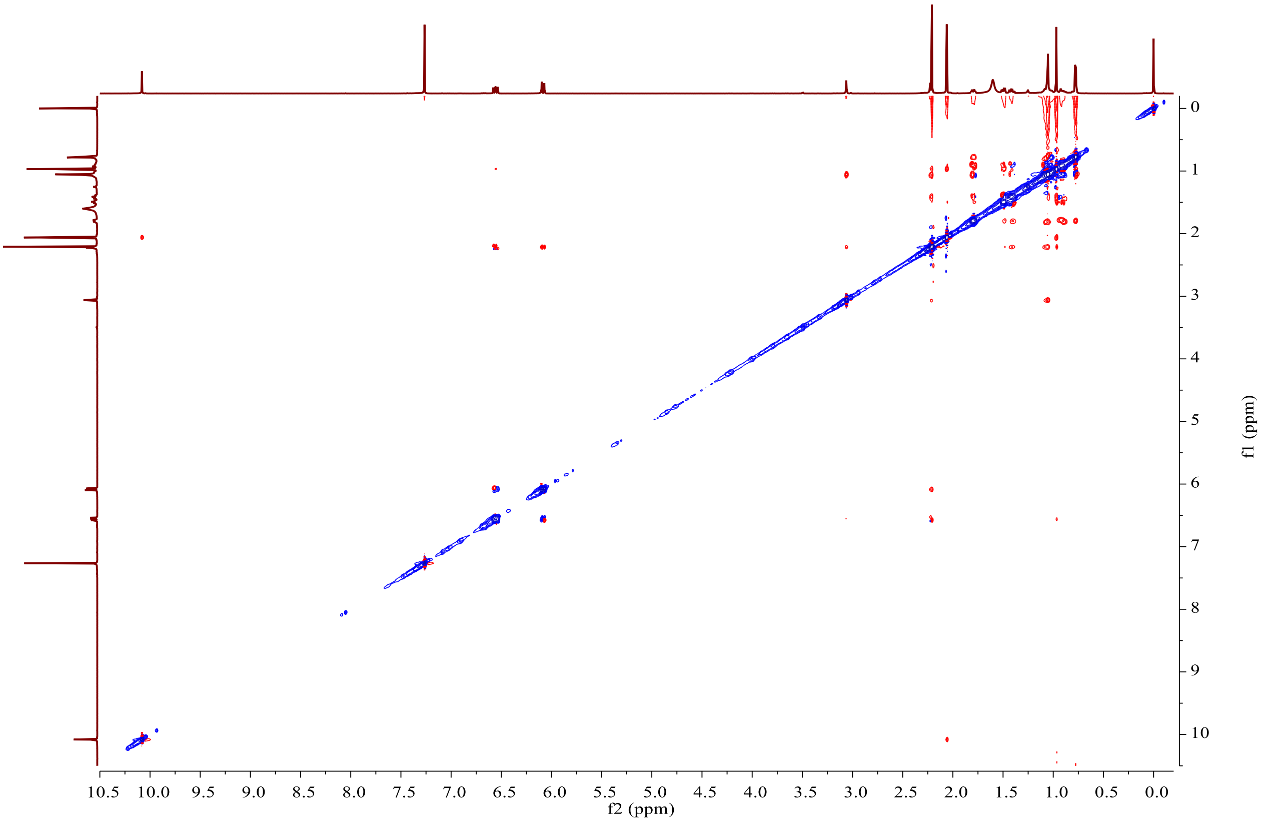

HRESIMS

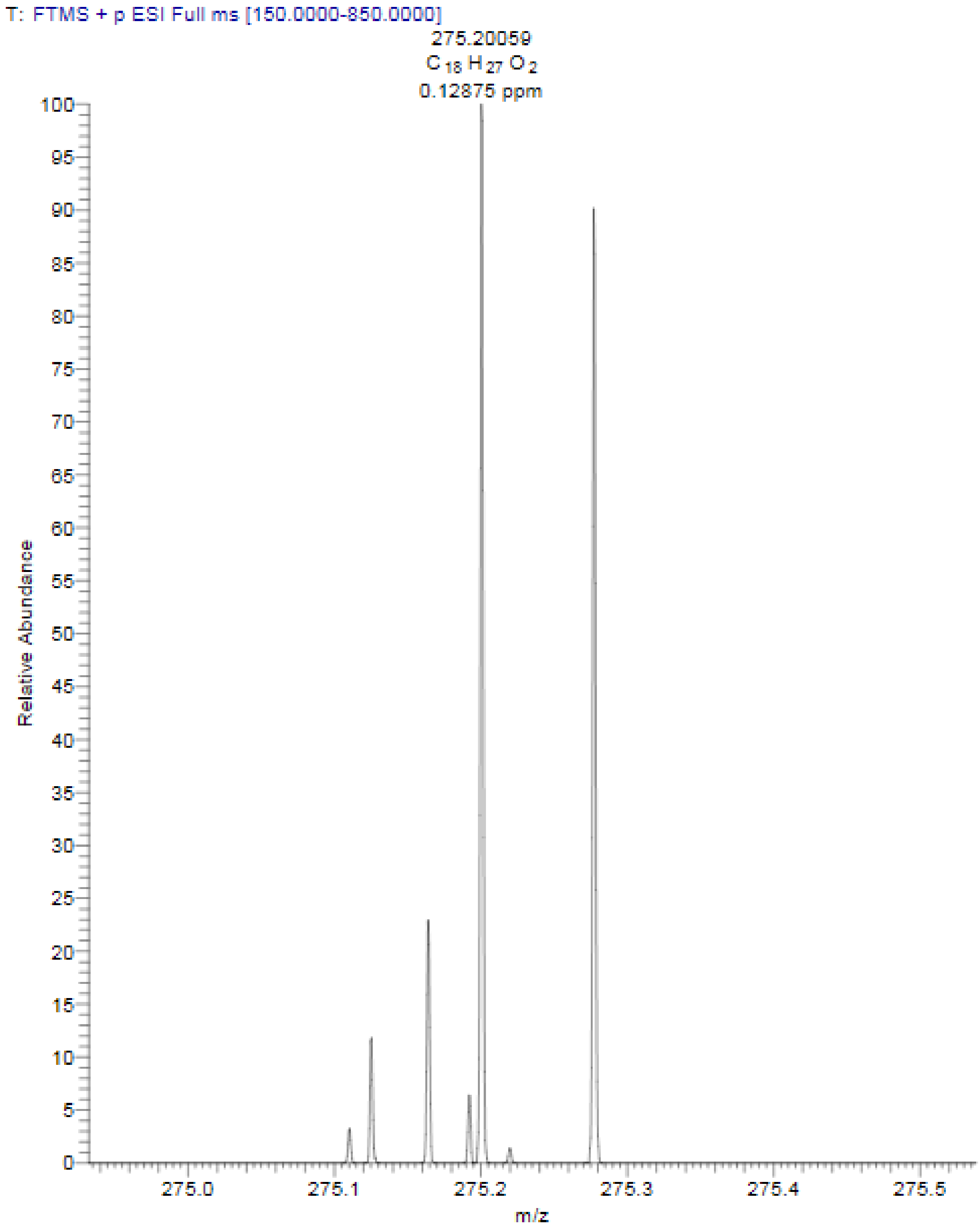
T: FTMS + p ESI Full ms [150.0000-850.0000]
275.20059
C 18 H 27 O 2
0.12875 ppm
Relative Abundance
m/z
275.0
275.1
275.2
275.3
275.4
275.5

CD spectra

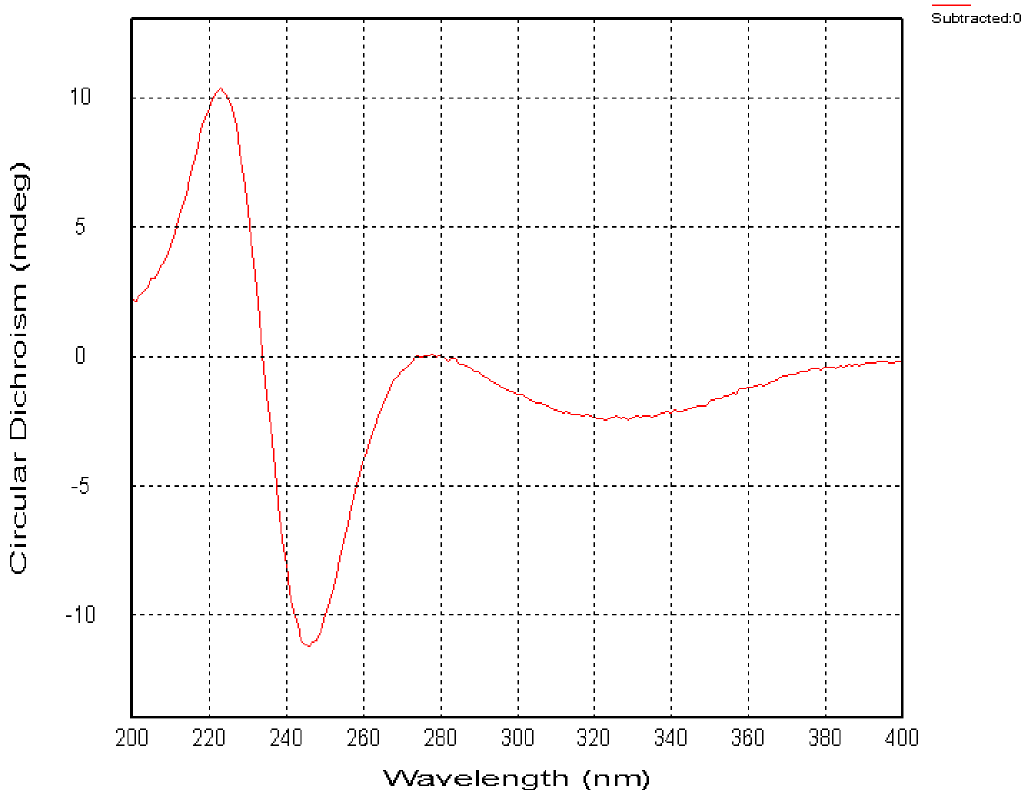

Subtracted:0
10
5
0
-5
-10
Circular Dichroism (mdeg)
200
220
240
260
280
300
320
340
360
380
400
Wavelength (nm)

S1.8 NMR, HRESIMS and CD spectra of bipolarisorokin H (8)

^{1}H NMR spectrum

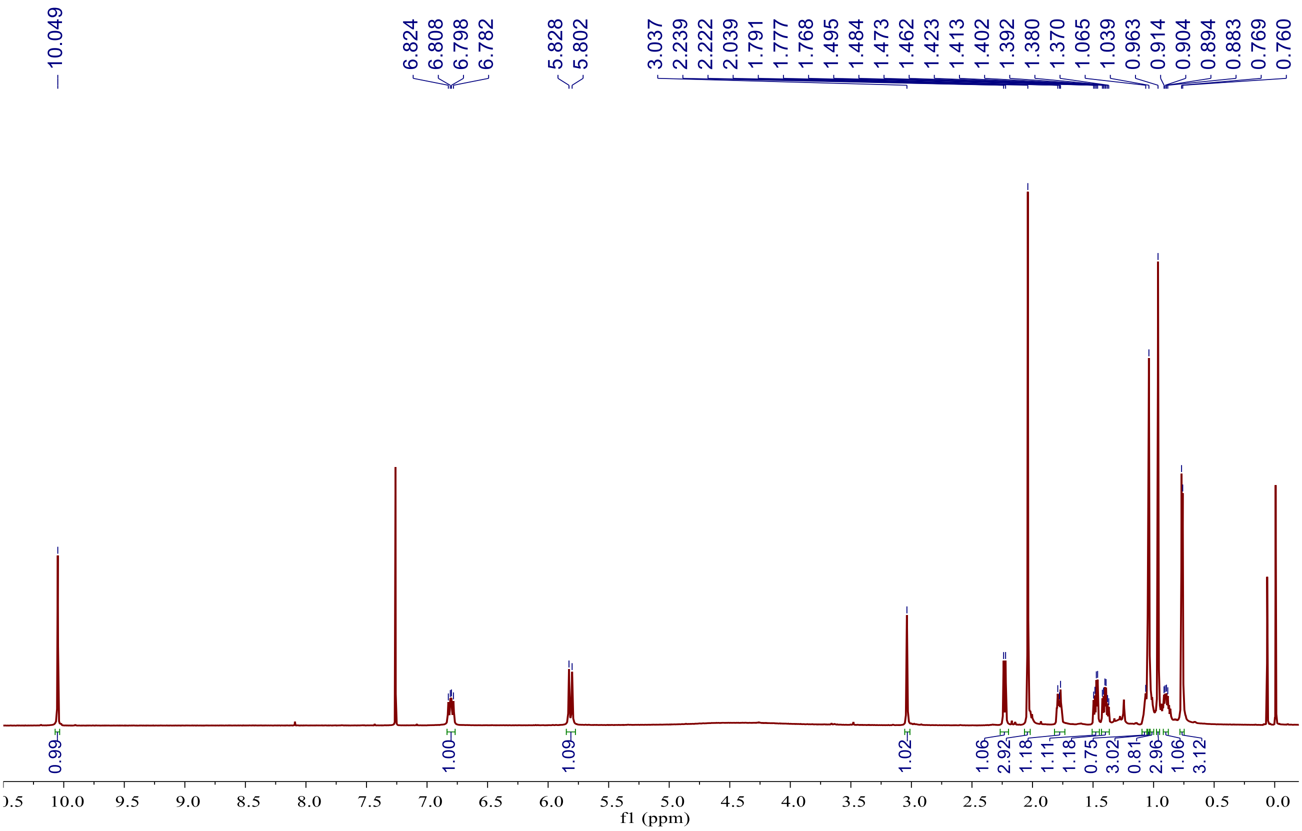

^{13}C NMR and DEPT spectra

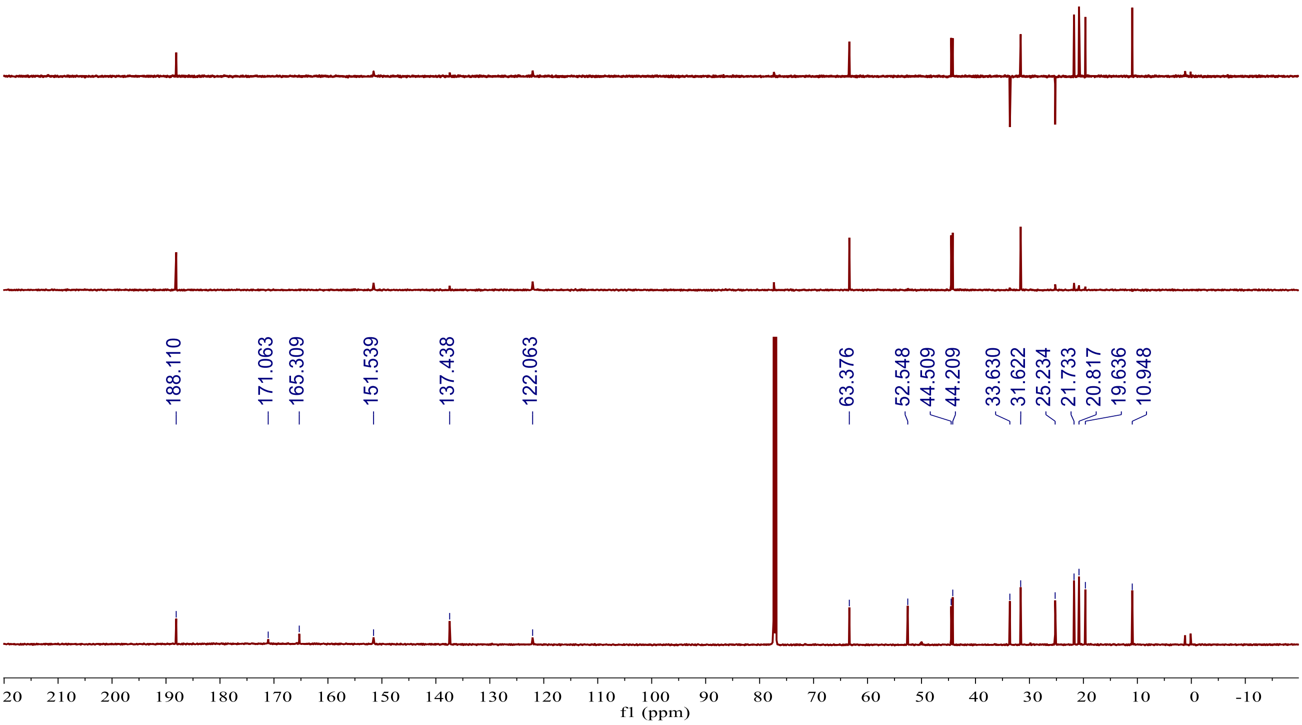

HSQC spectrum

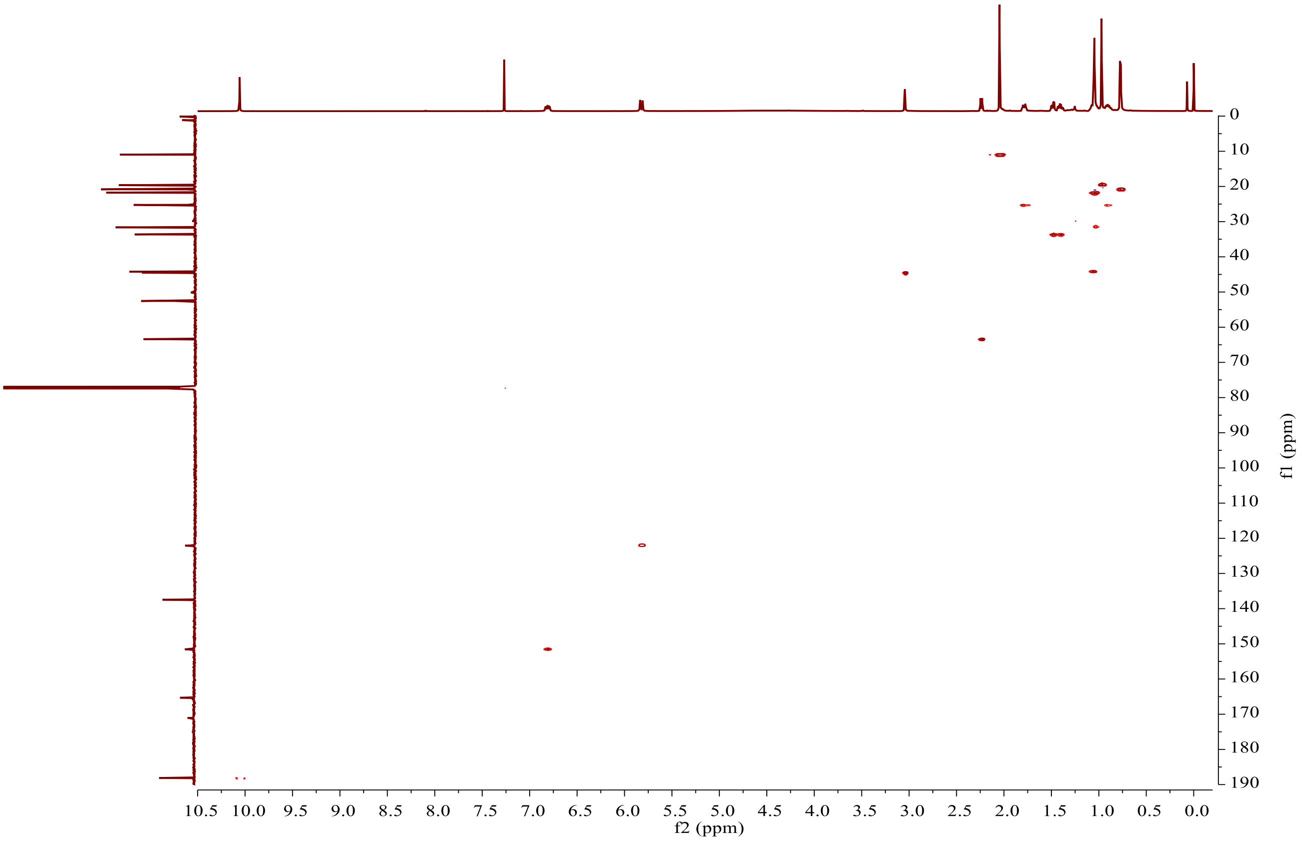

HMBC spectrum

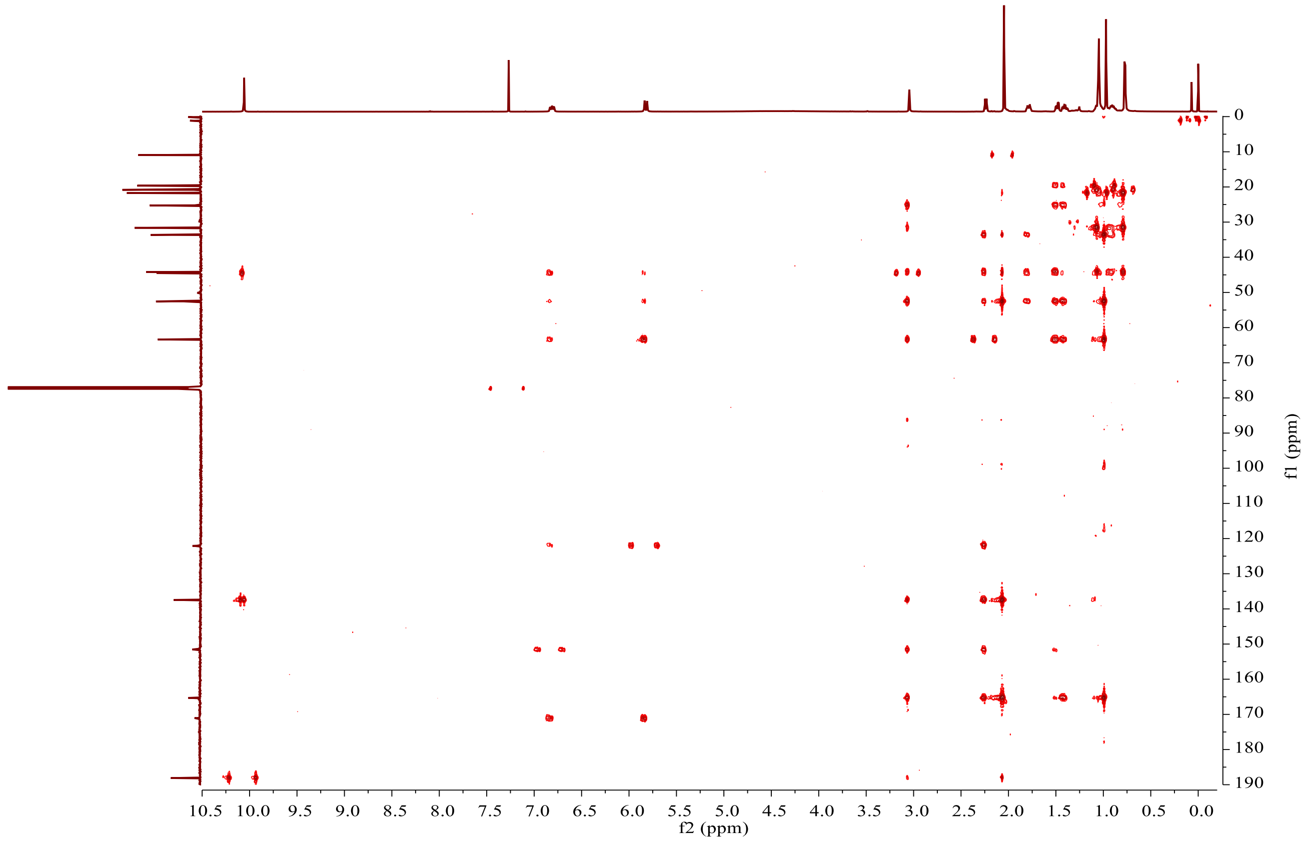

^1H-^1H COSY spectrum

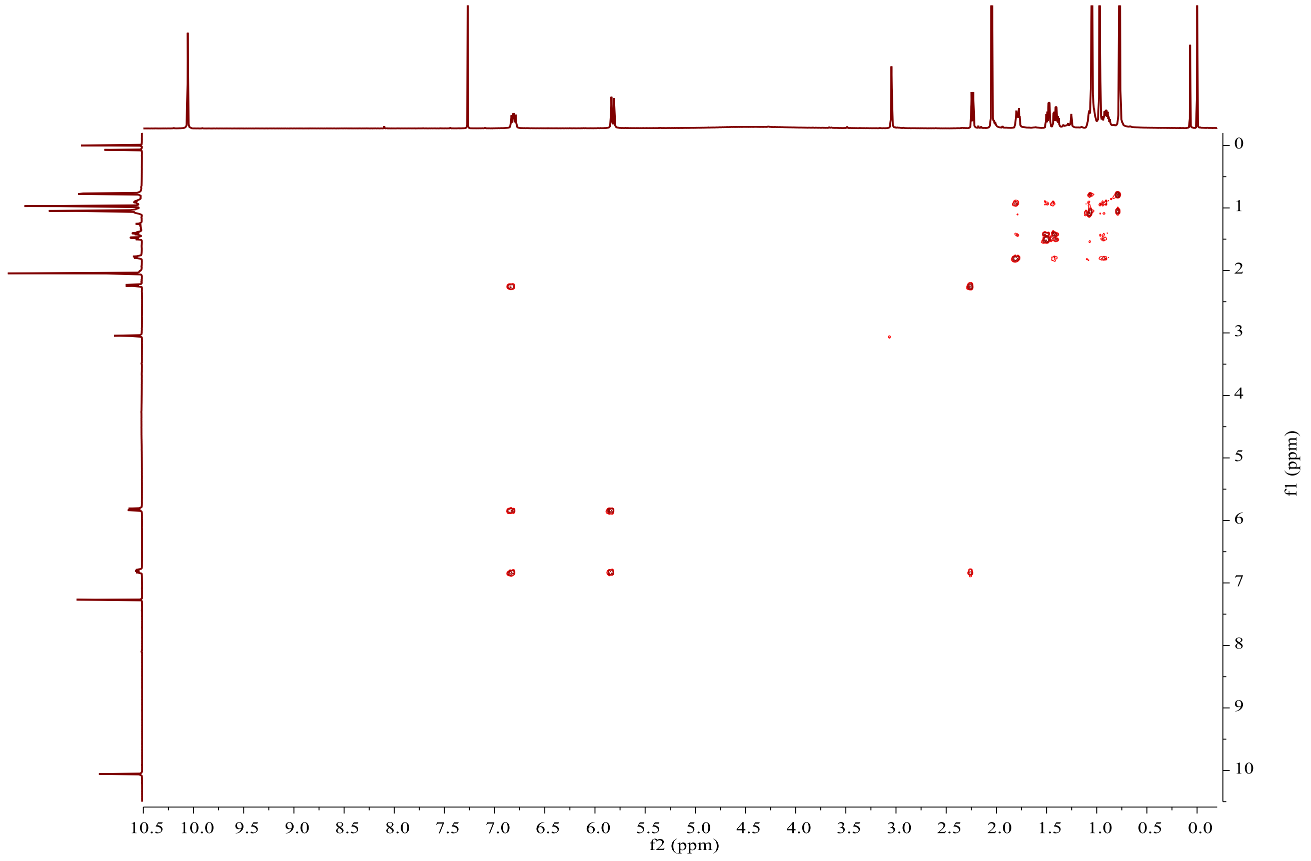

ROESY spectrum

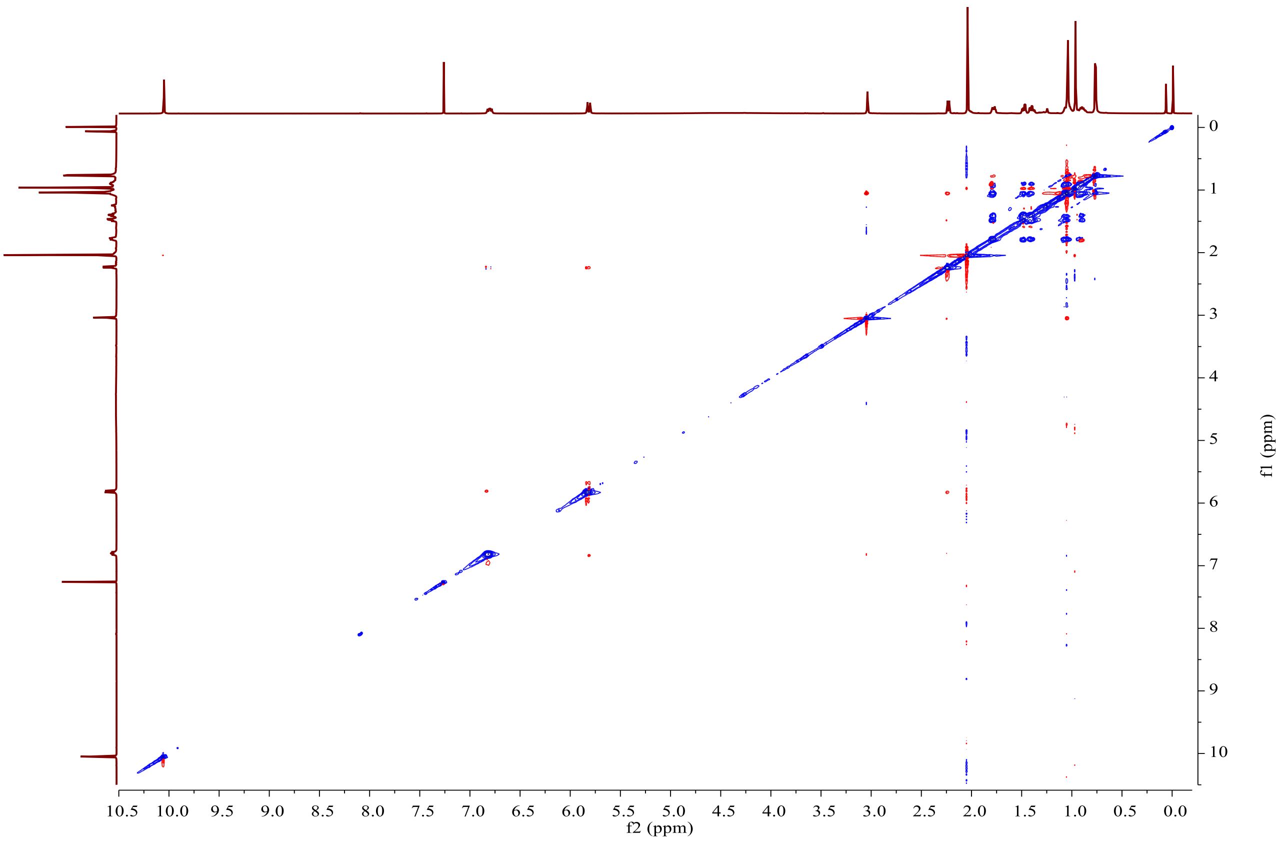

HRESIMS

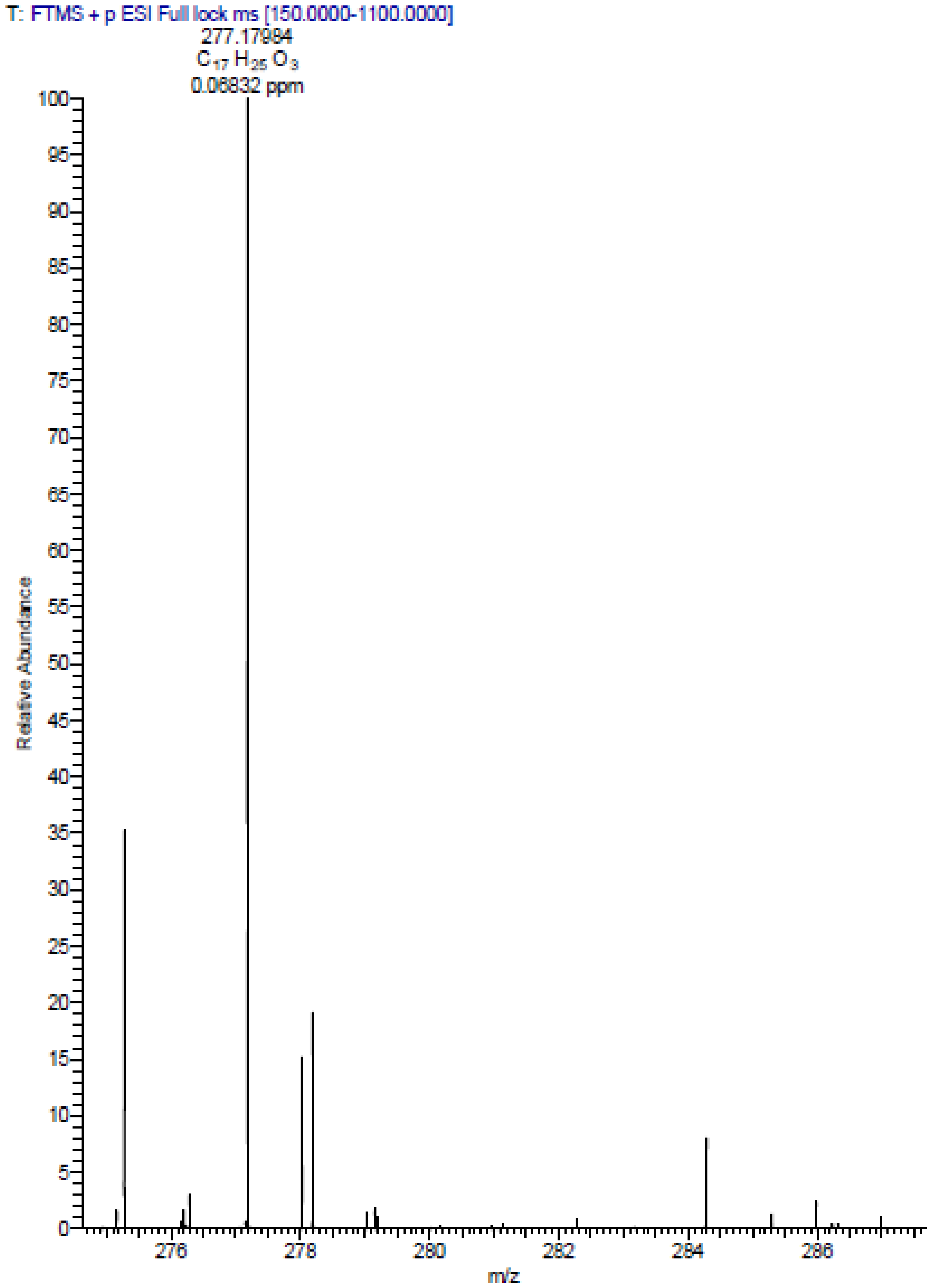
T: FTMS + p ESI Full lock ms [150.0000-1100.0000]
277.17984
C17 H25 O3
0.06832 ppm
Relative Abundance
m/z

CD spectra

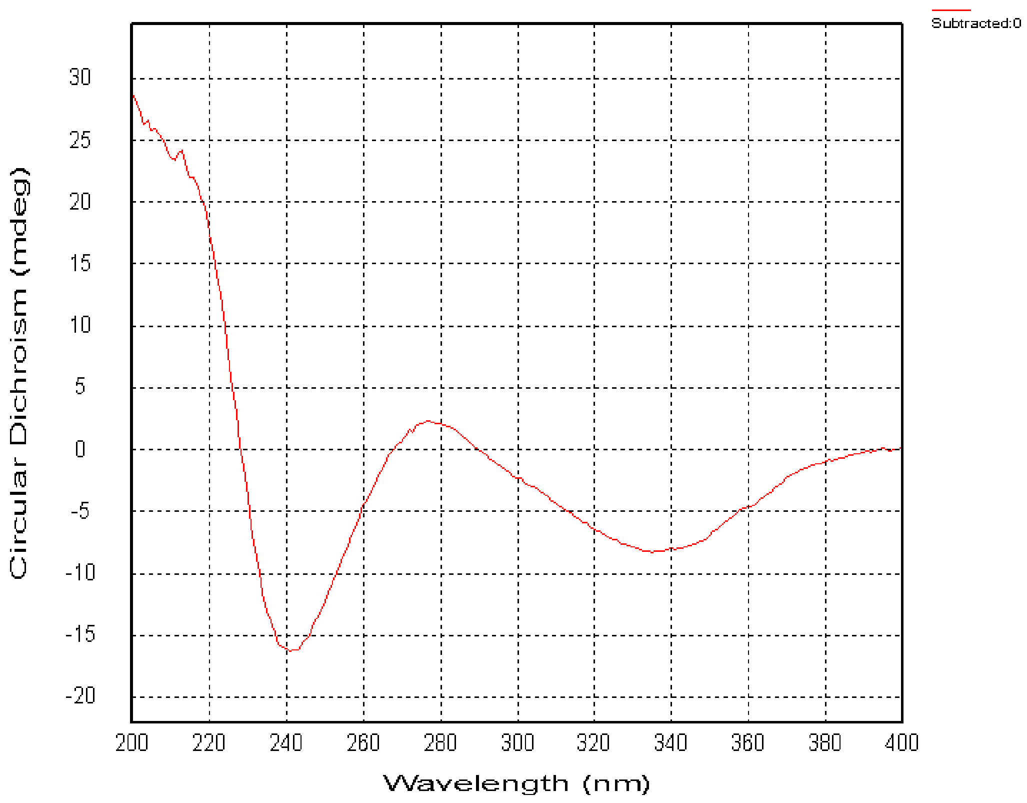

Subtracted:0
Circular Dichroism (mdeg)
30
25
20
15
10
5
0
-5
-10
-15
-20
200
220
240
260
280
300
320
340
360
380
400
Wavelength (nm)

S1.9 NMR and HRESIMS spectra of bipolarisorokin I (9)

^{1}H NMR spectrum

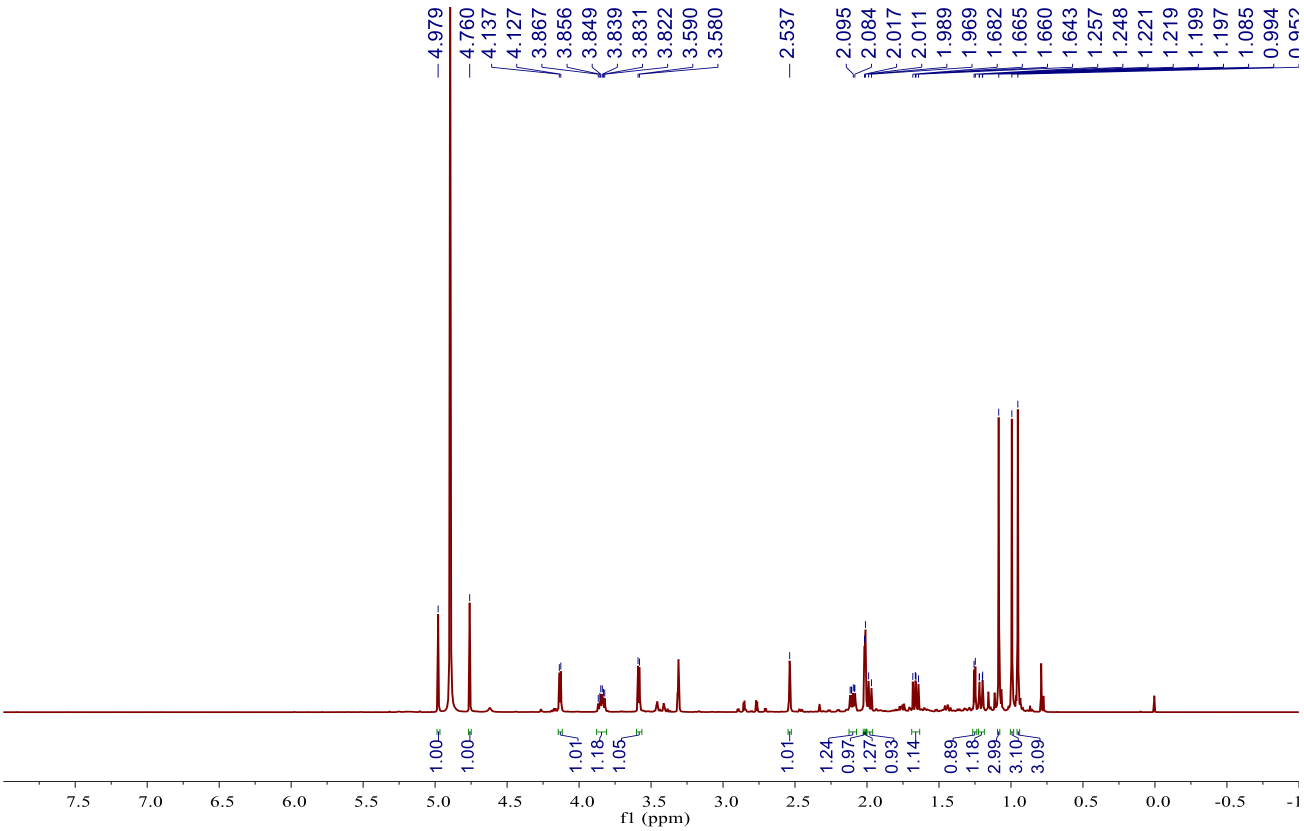

^{13}C NMR and DEPT spectra

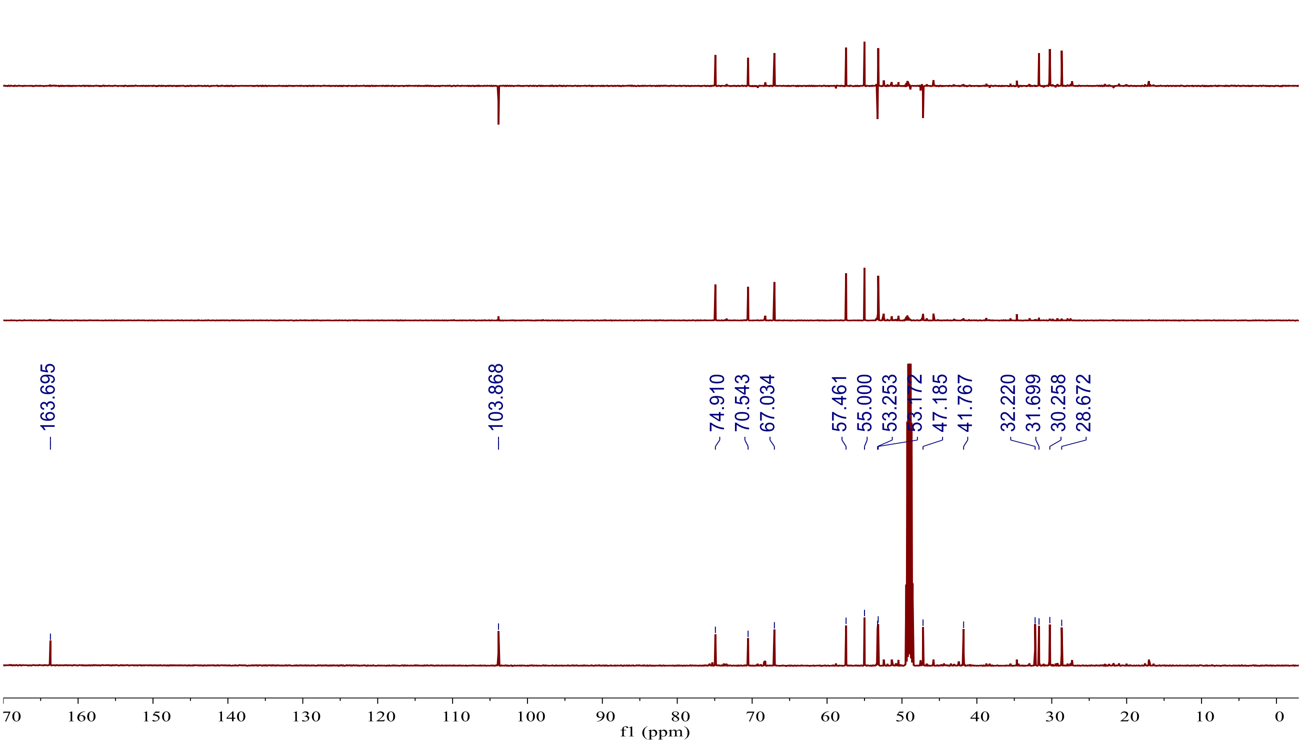

HSQC spectrum

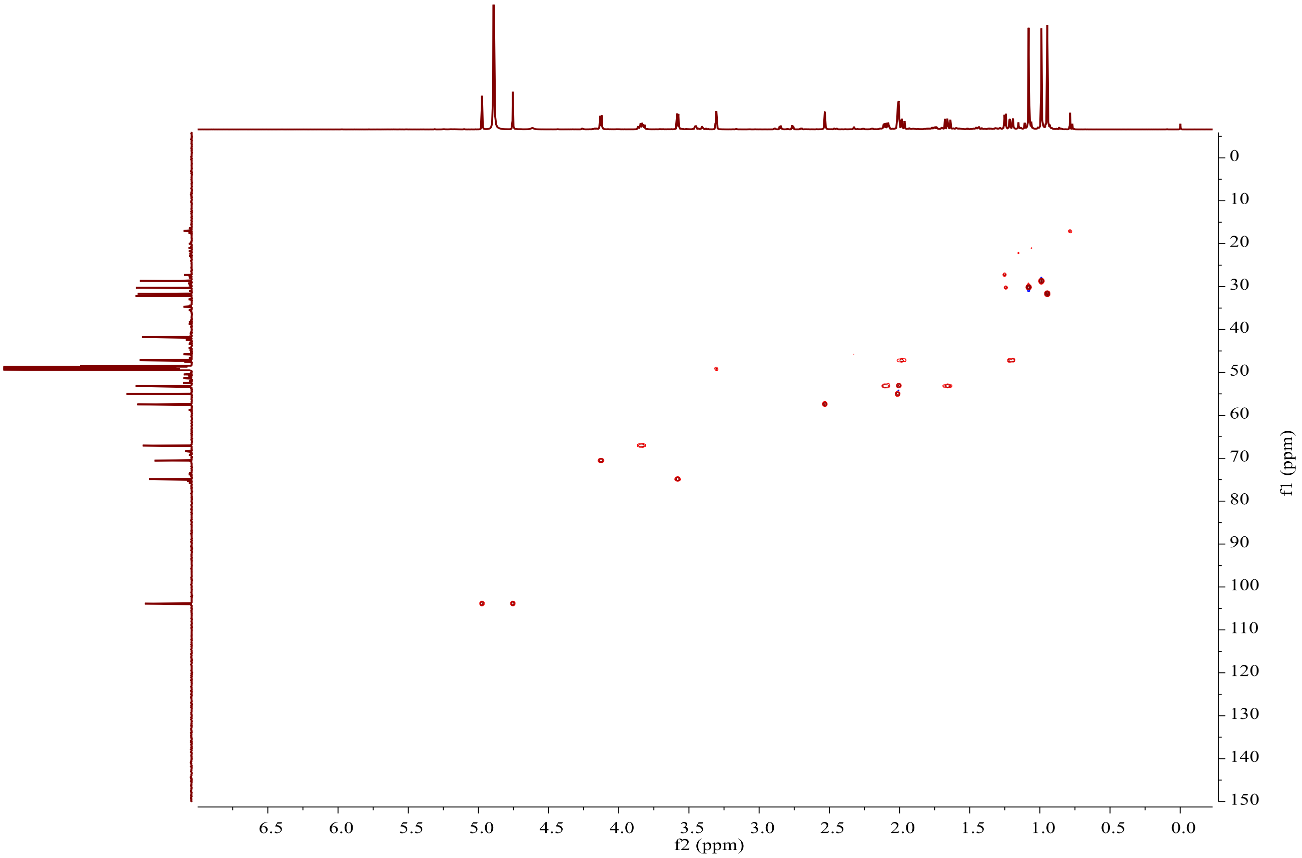

HMBC spectrum

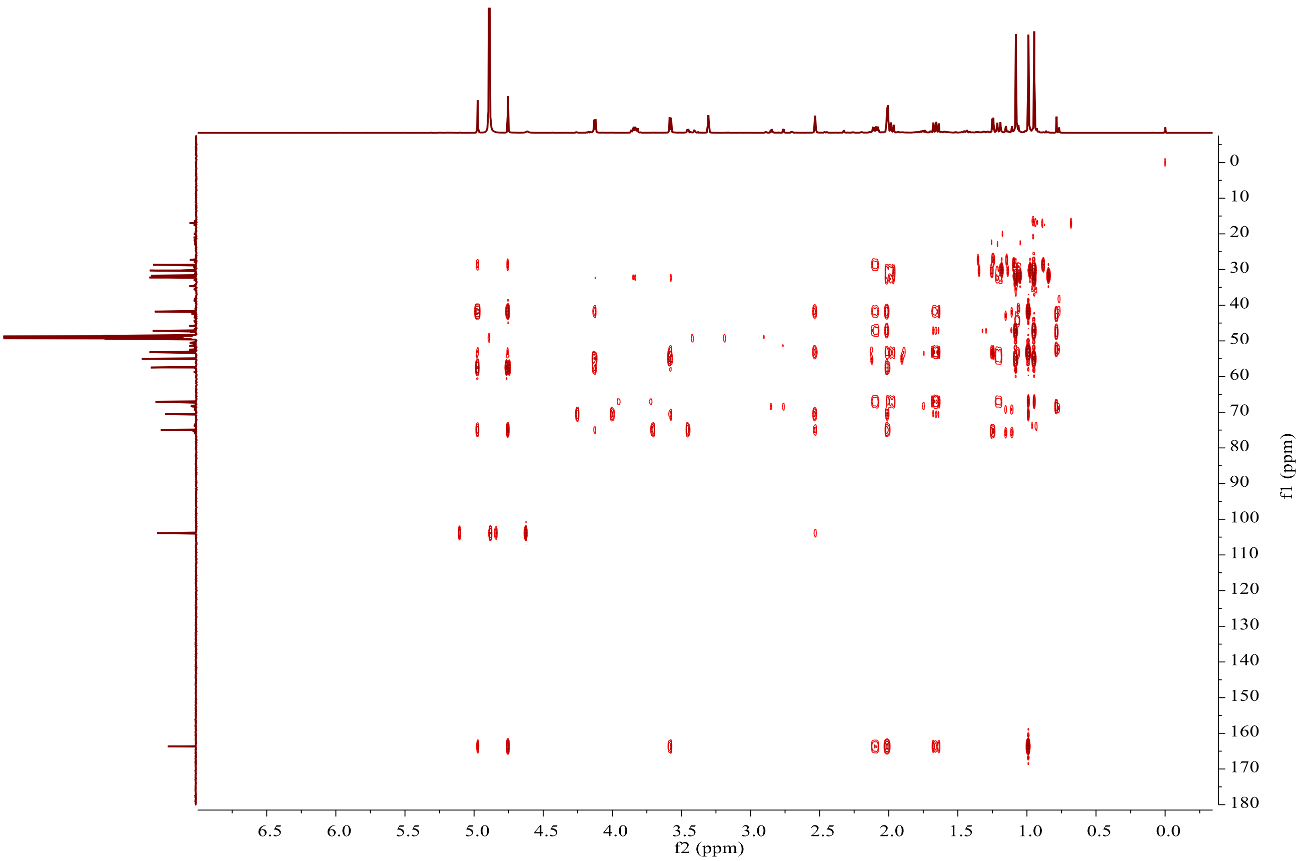

^1H-^1H COSY spectrum

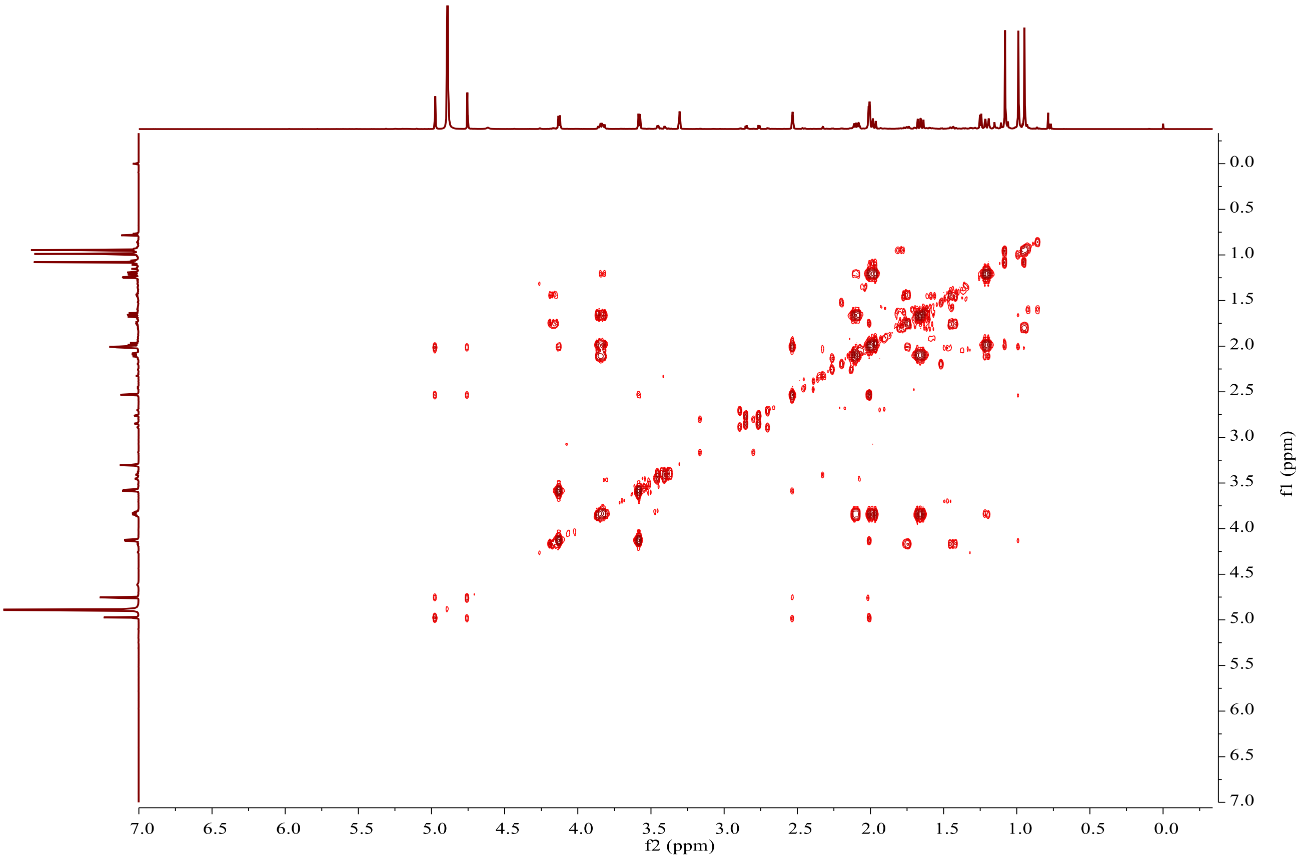

ROESY spectrum

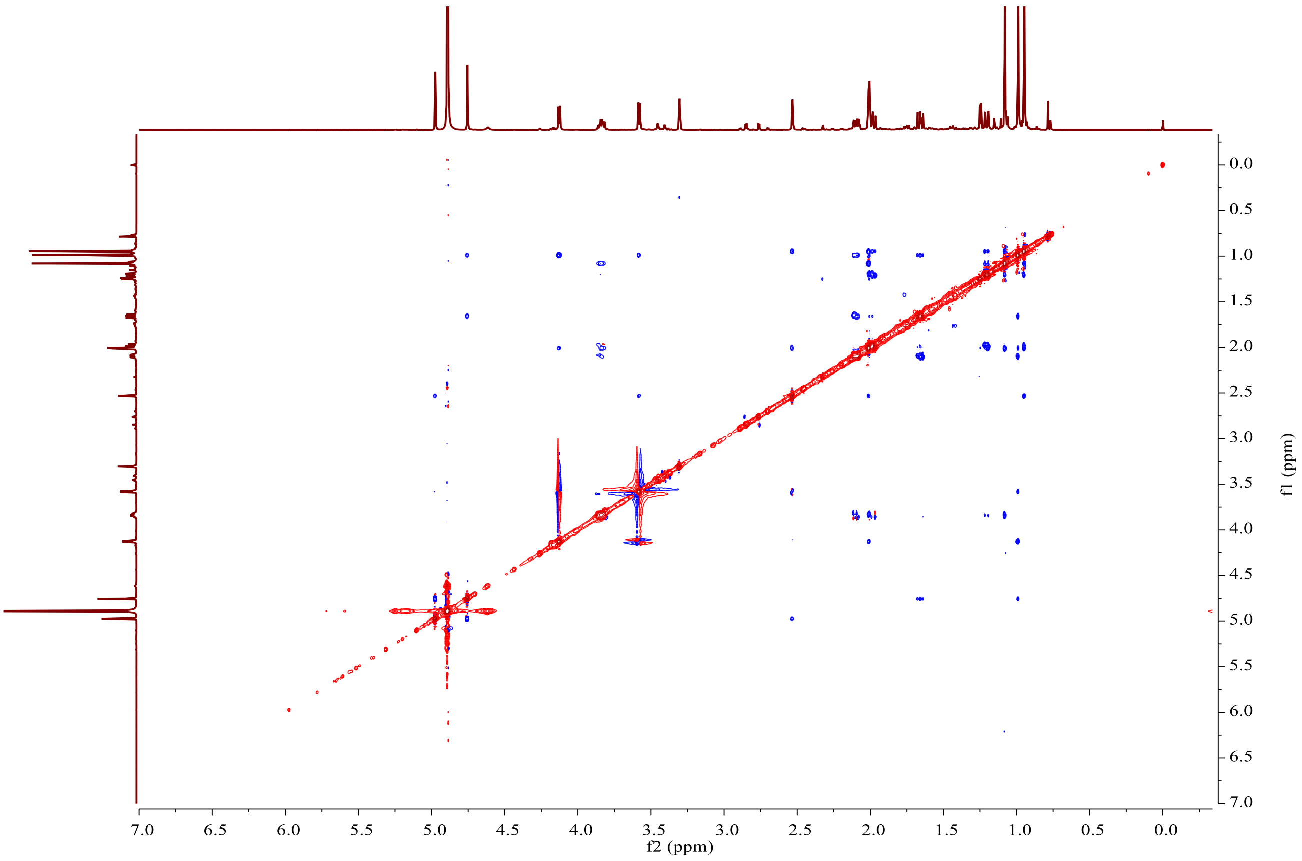

HRESIMS

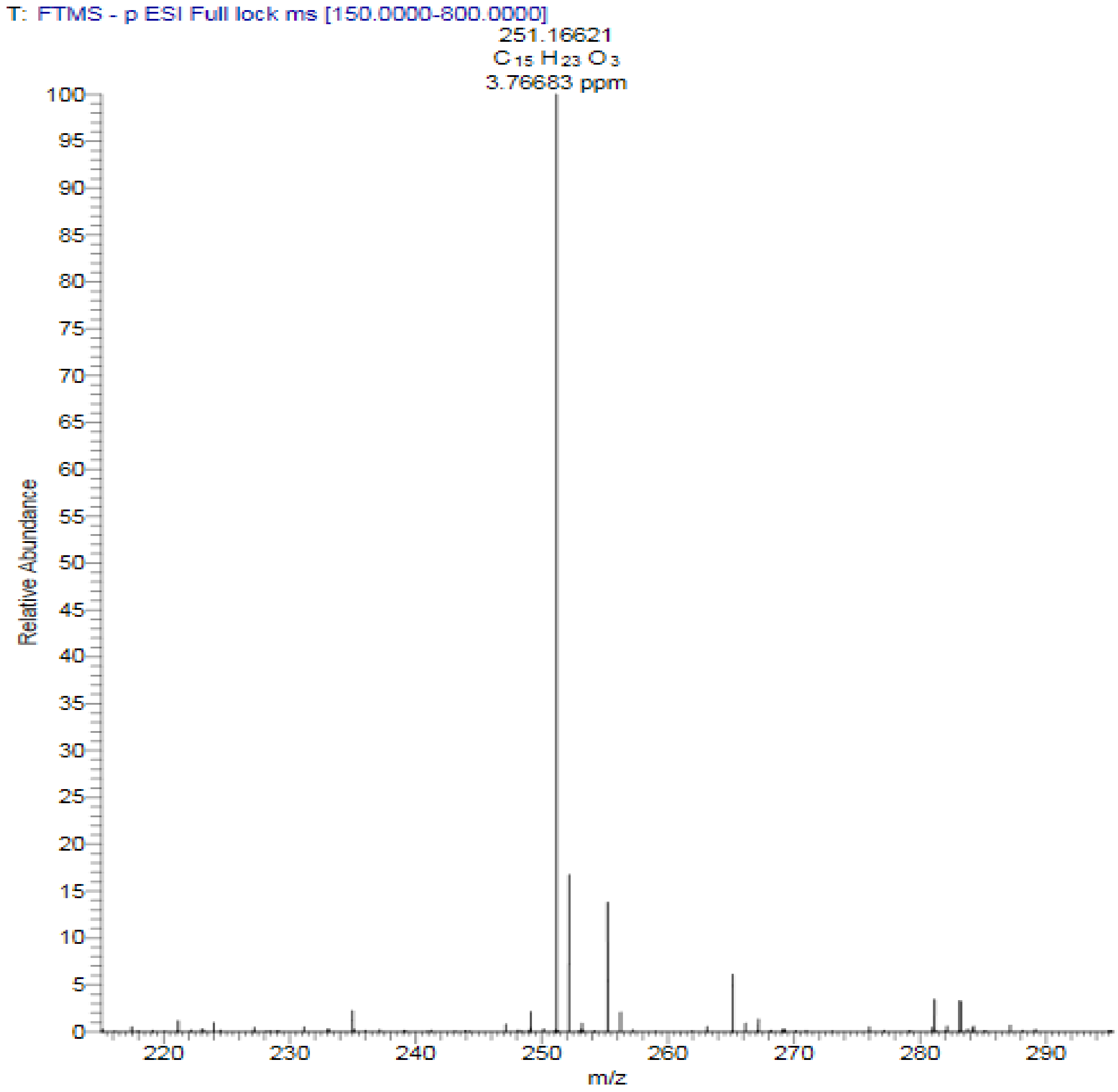

T: FTMS - p ESI Full lock ms [150.0000-800.0000]
251.16621
C 15 H 23 O 3
3.76683 ppm
Relative Abundance
m/z

S1.10 NMR, HRESIMS and CD spectra of bipolarithone A (10)

^{1}H NMR spectrum

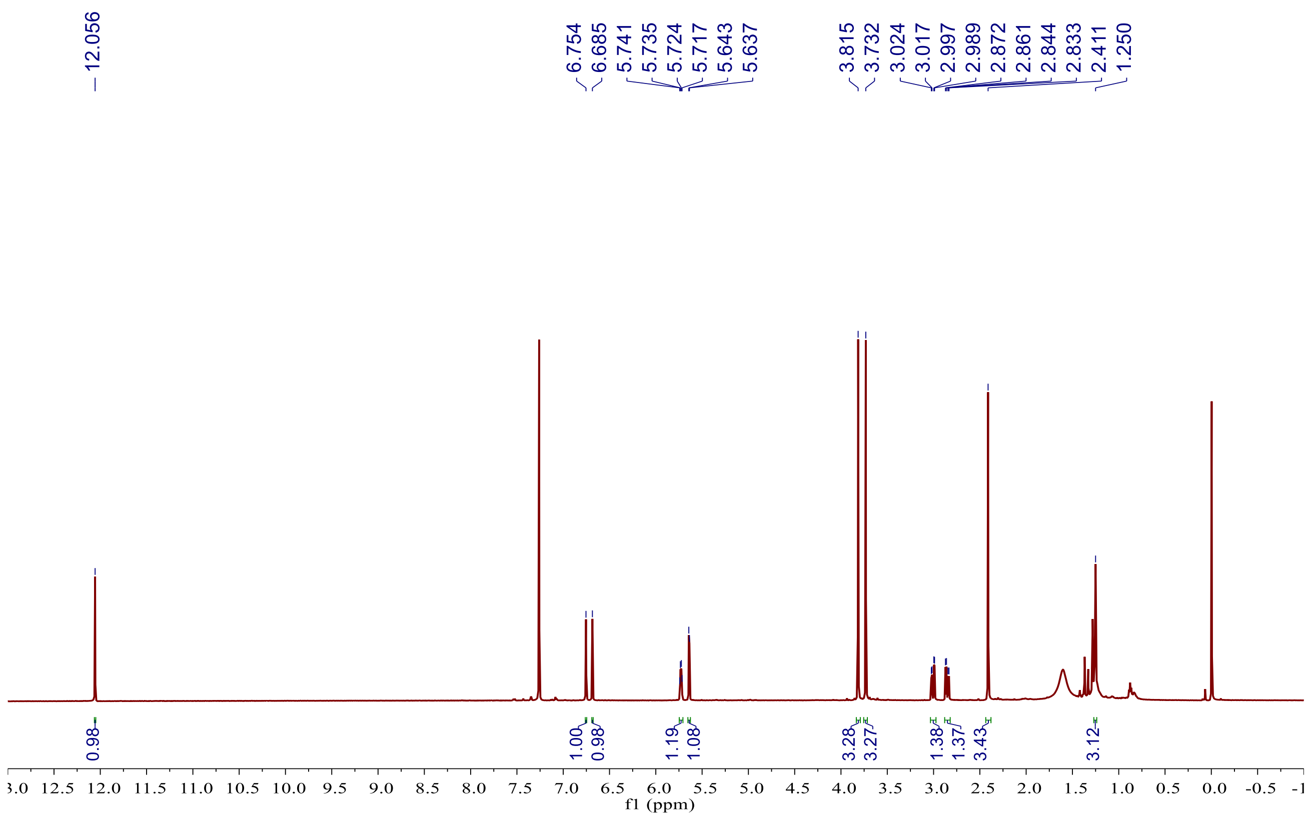

^{13}C NMR and DEPT spectra

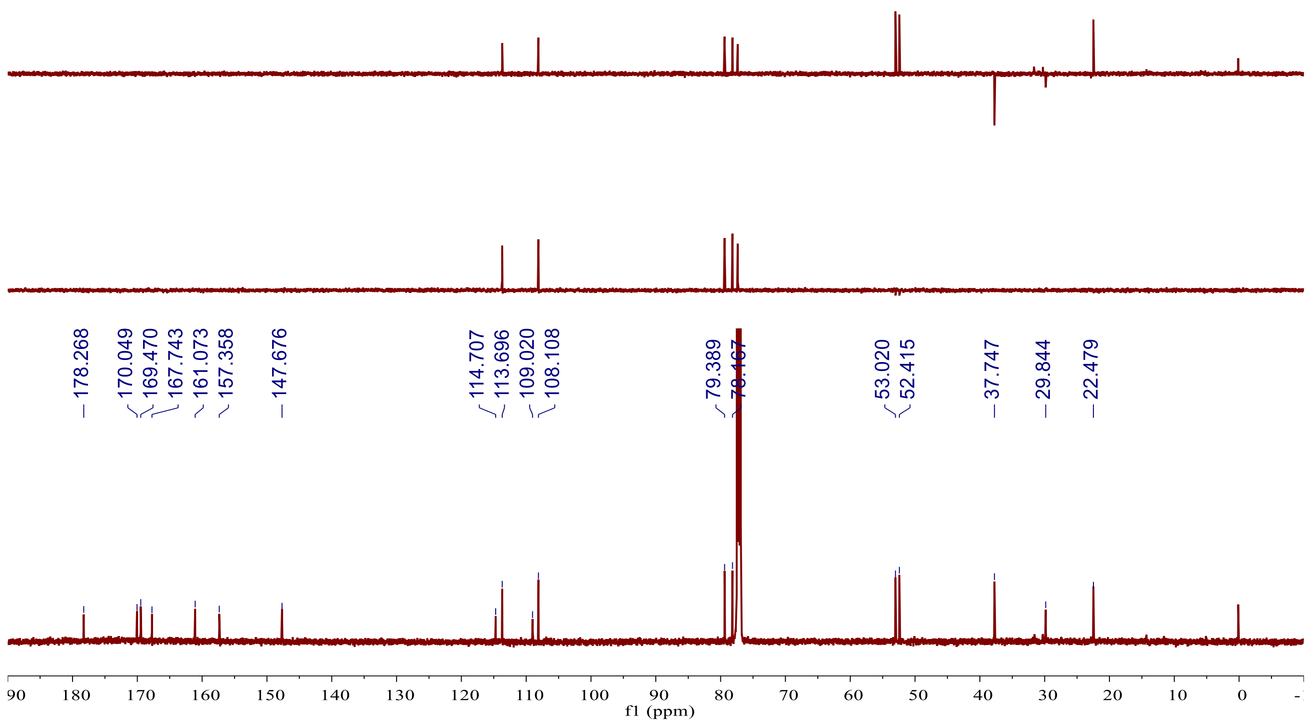

HSQC spectrum

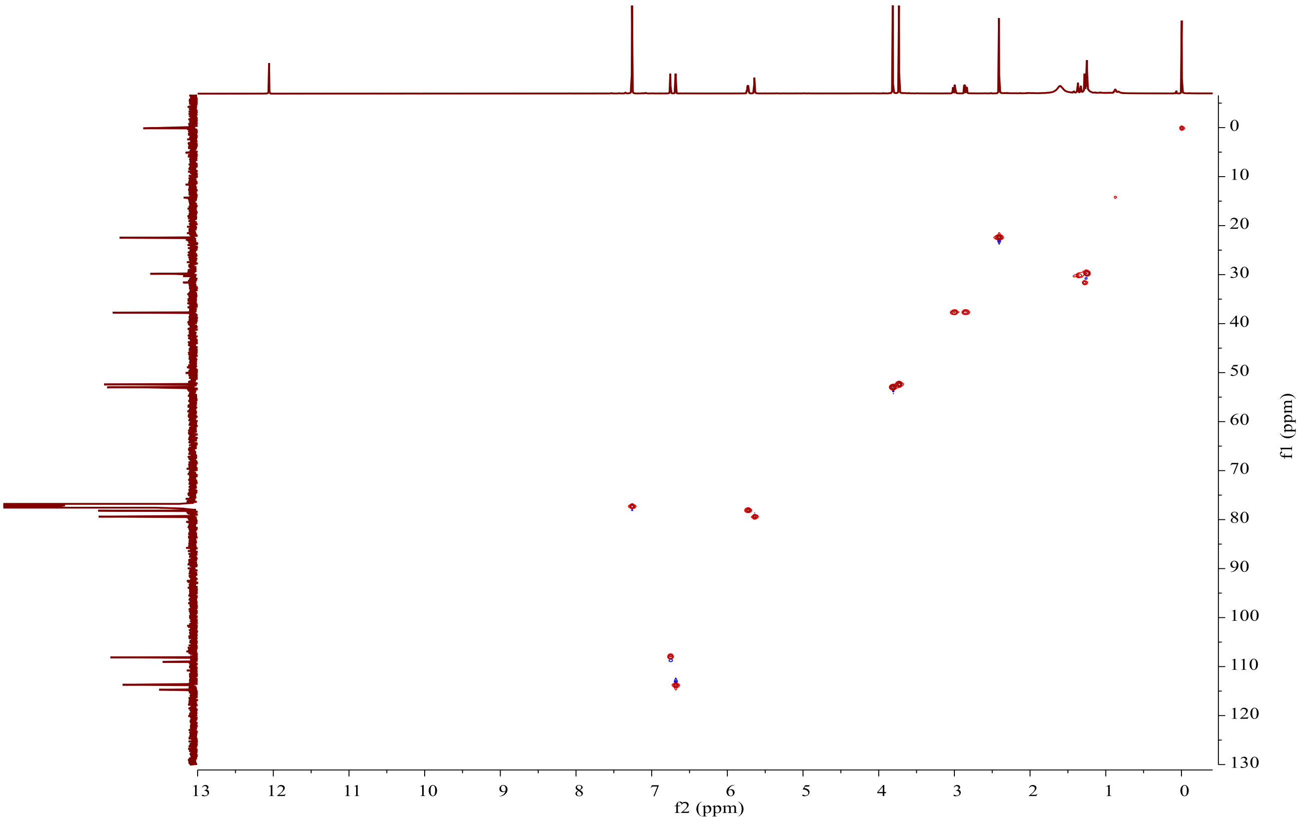

HMBC spectrum

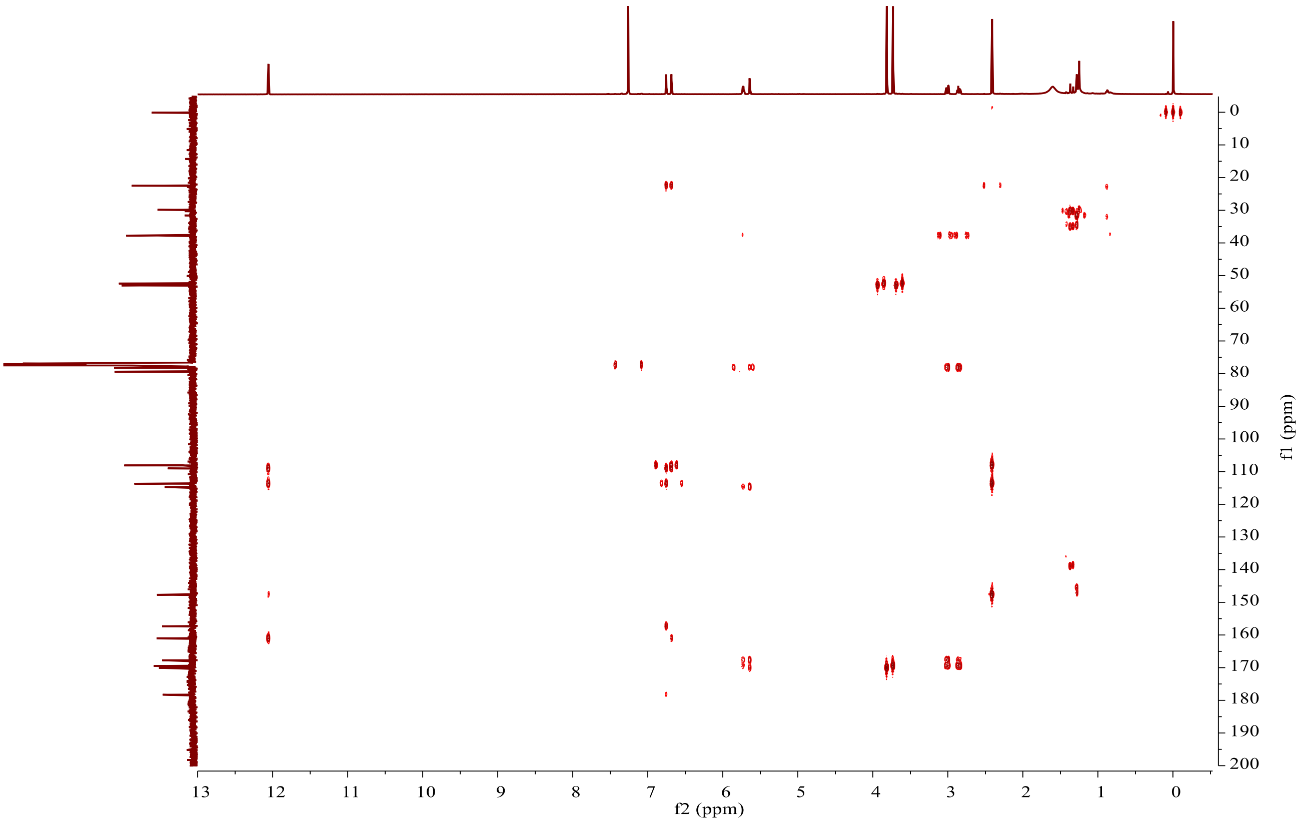

^1H-^1H COSY spectrum

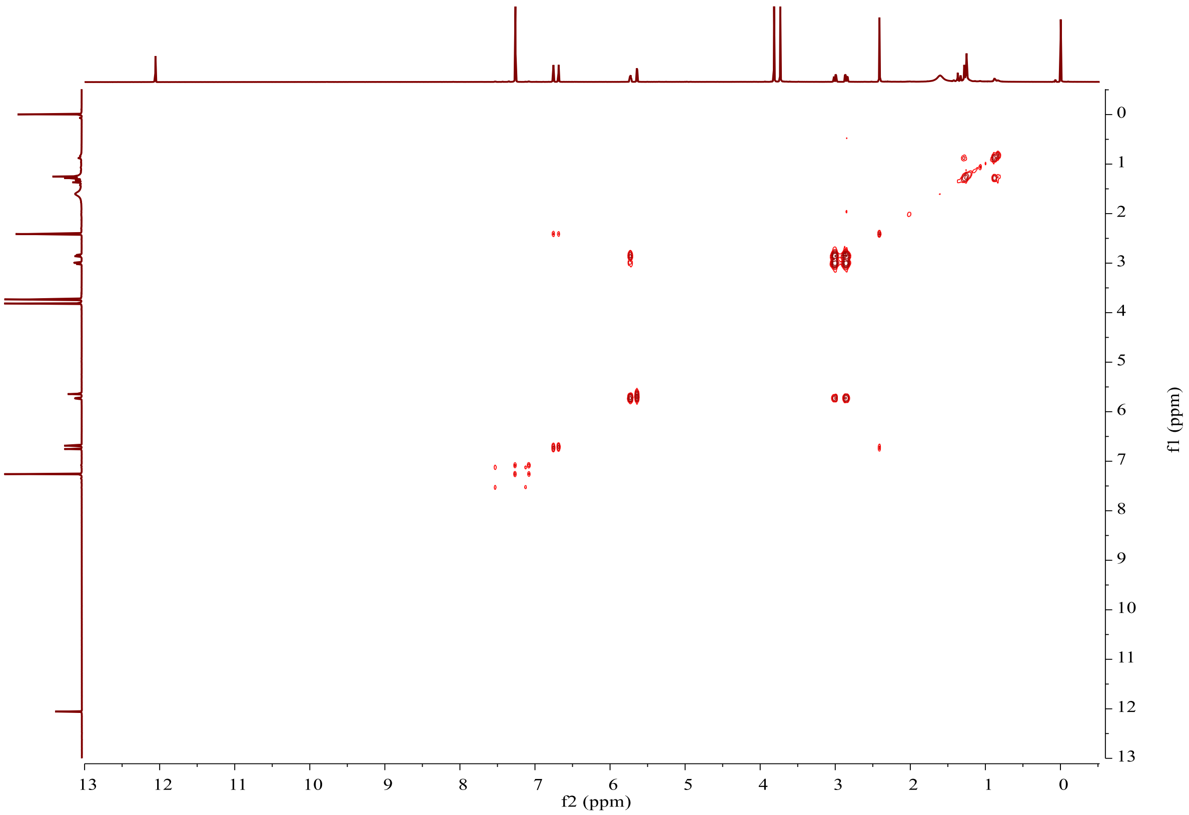

ROESY spectrum

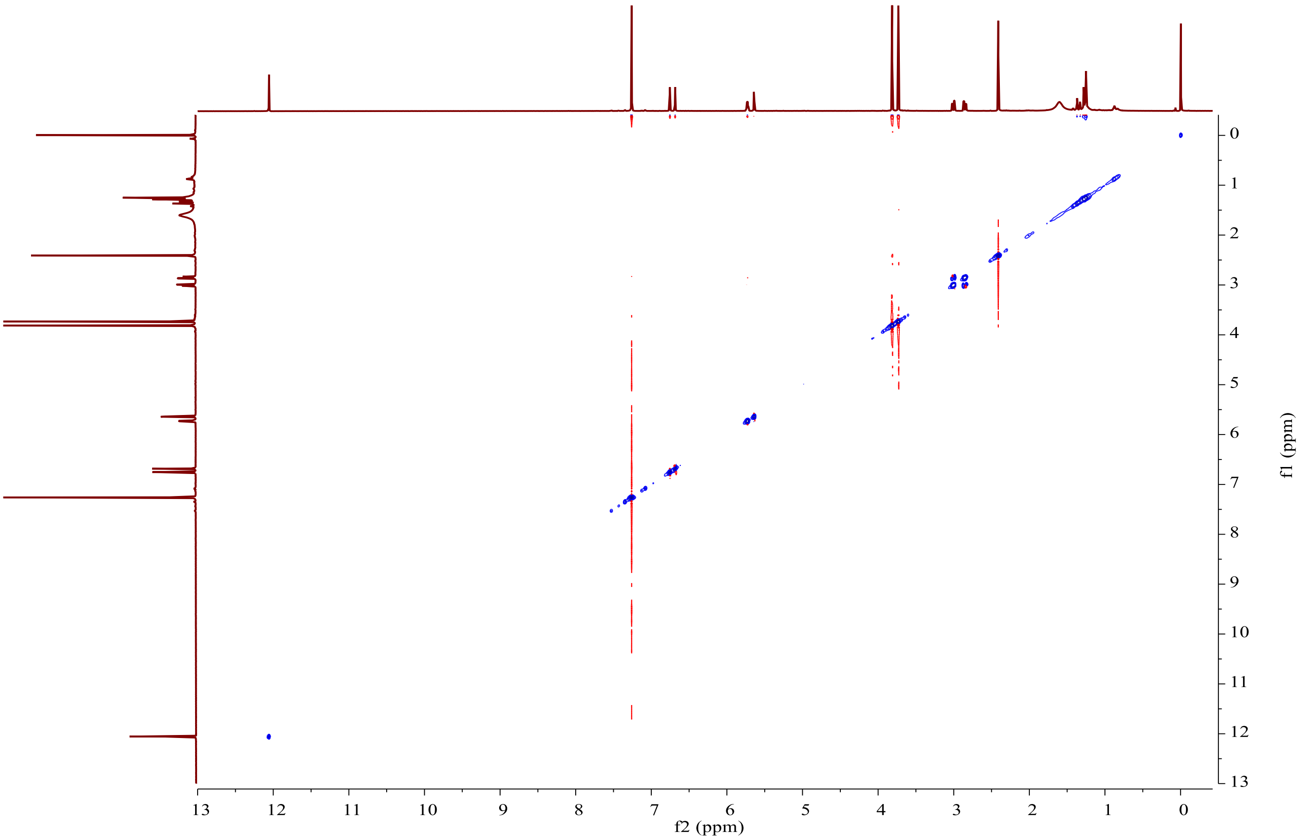

HRESIMS

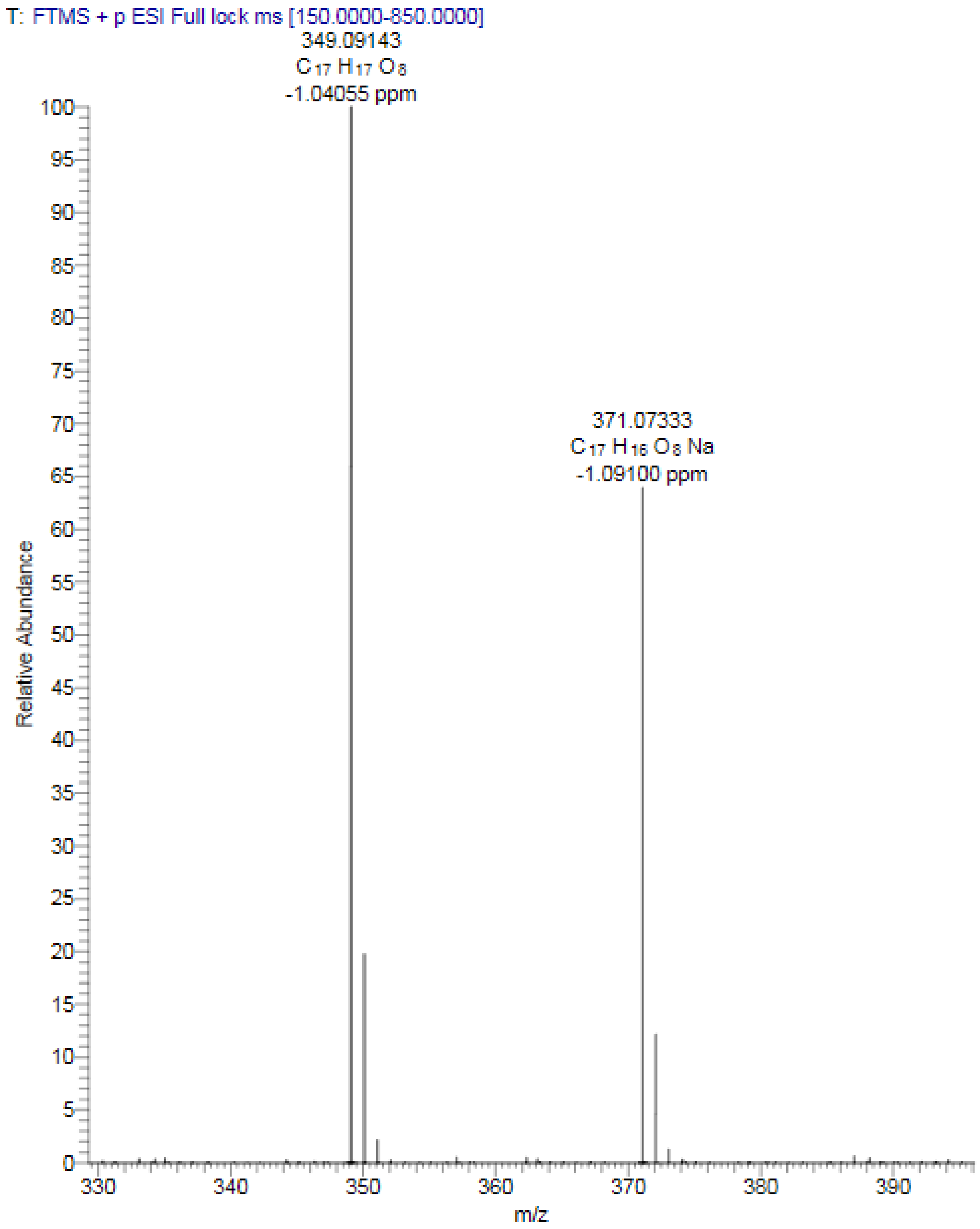
T: FTMS + p ESI Full lock ms [150.0000-850.0000]
349.09143
C 17 H 17 O 8
-1.04055 ppm
371.07333
C 17 H 16 O 8 Na
-1.09100 ppm
Relative Abundance
m/z
100
95
90
85
80
75
70
65
60
55
50
45
40
35
30
25
20
15
10
5
0
330
340
350
360
370
380
390

CD spectra

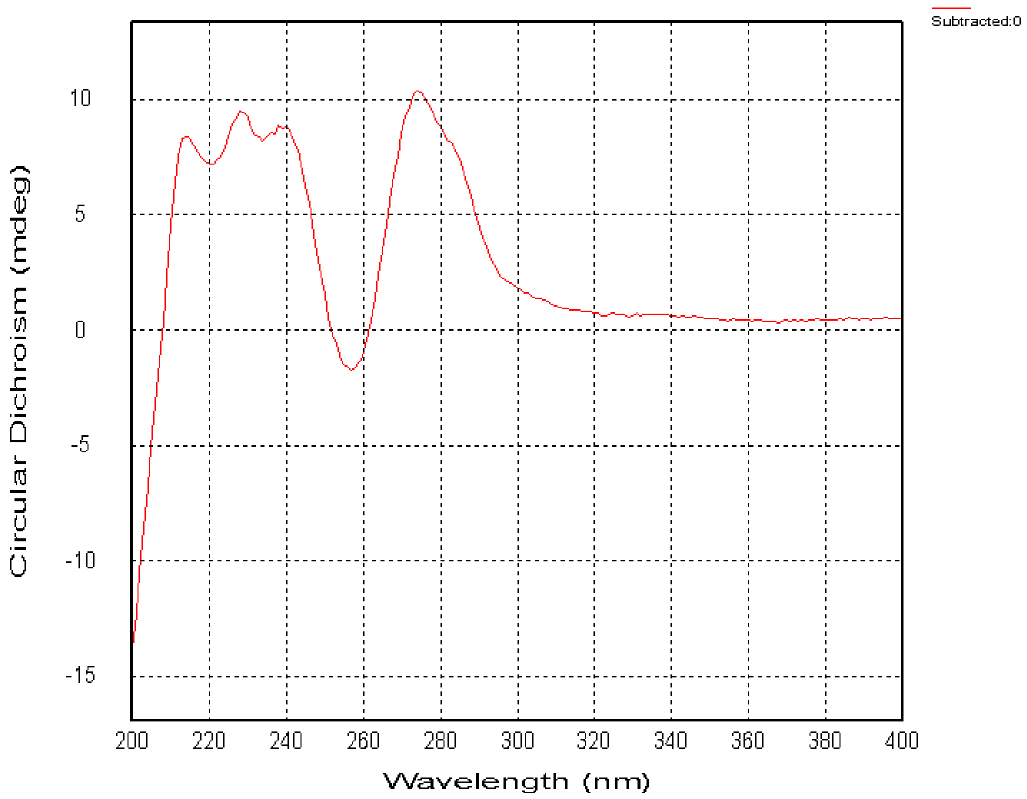

Subtracted:0
Circular Dichroism (mdeg)
10
5
0
-5
-10
-15
200
220
240
260
280
300
320
340
360
380
400
Wavelength (nm)

S1.11 NMR, HRESIMS and CD spectra of bipolarithone B (11)

^{1}H NMR spectrum

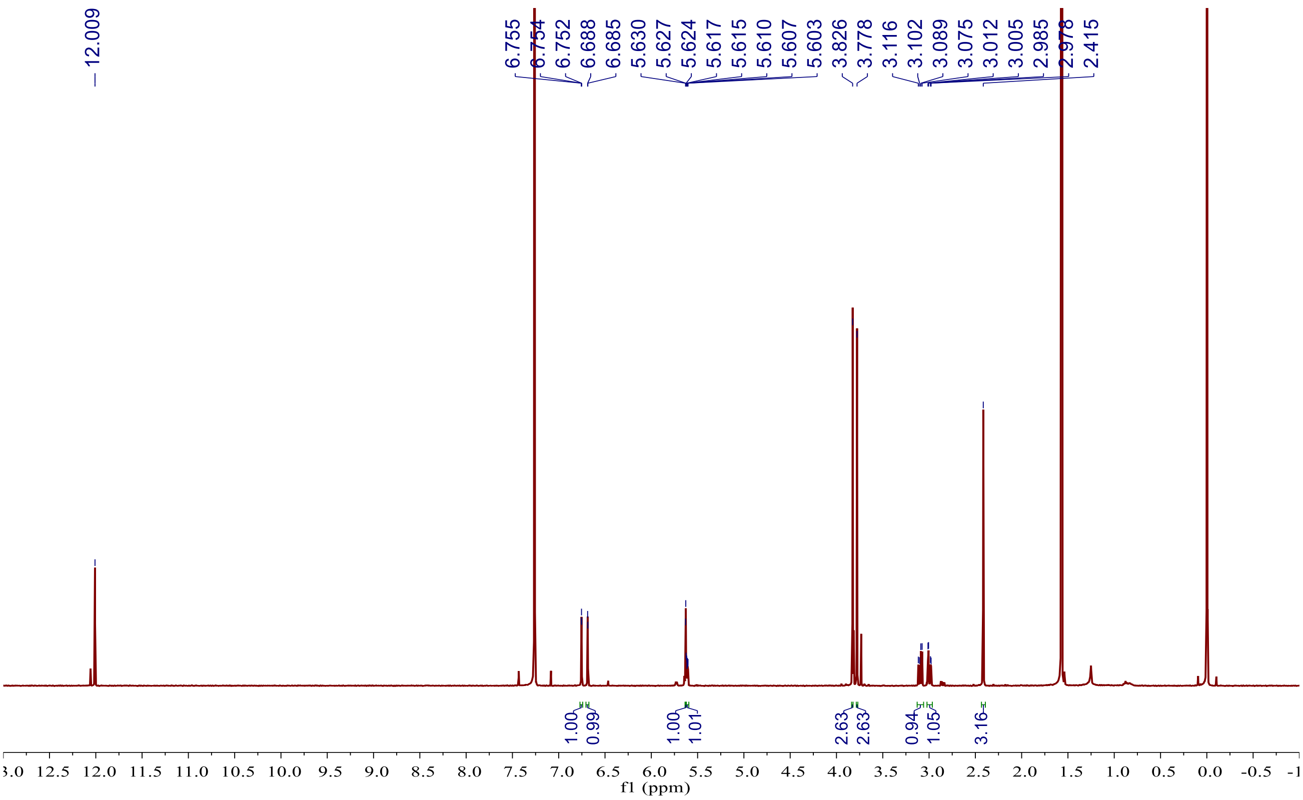

^{13}C NMR and DEPT spectra

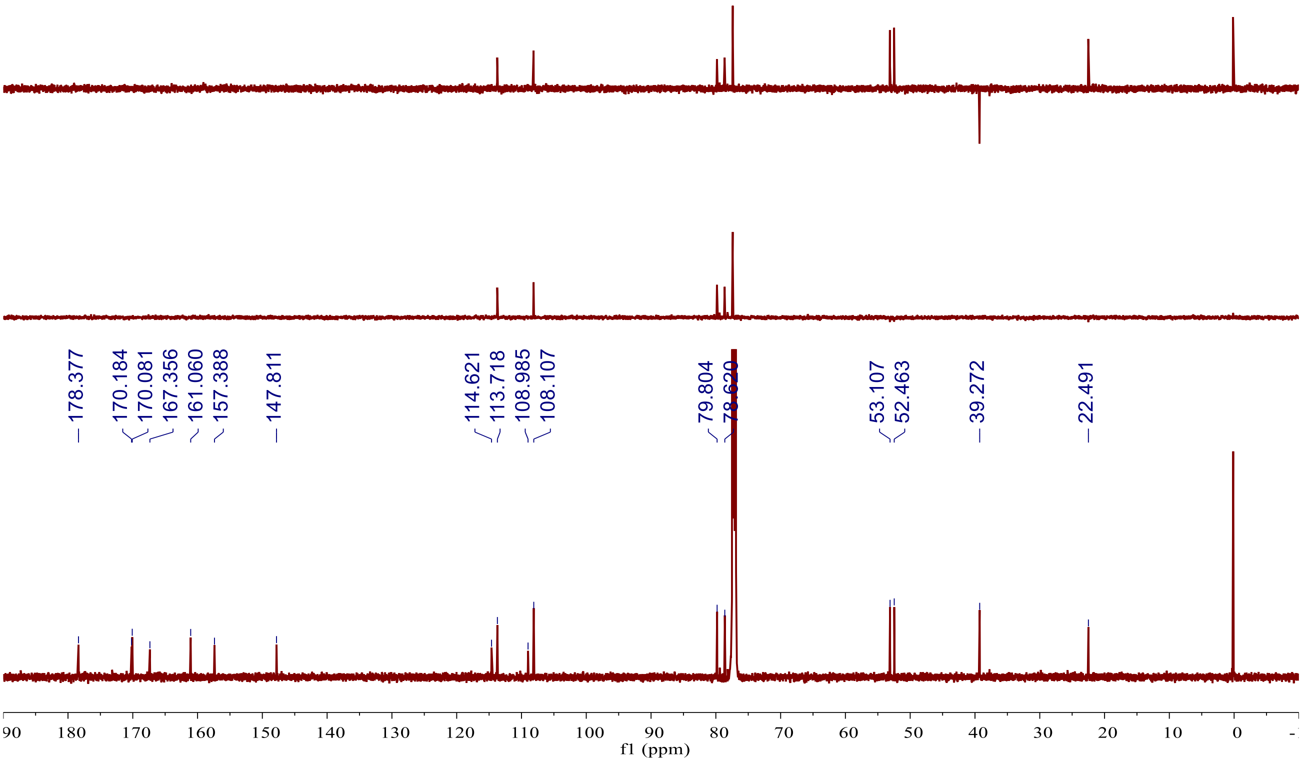

HSQC spectrum

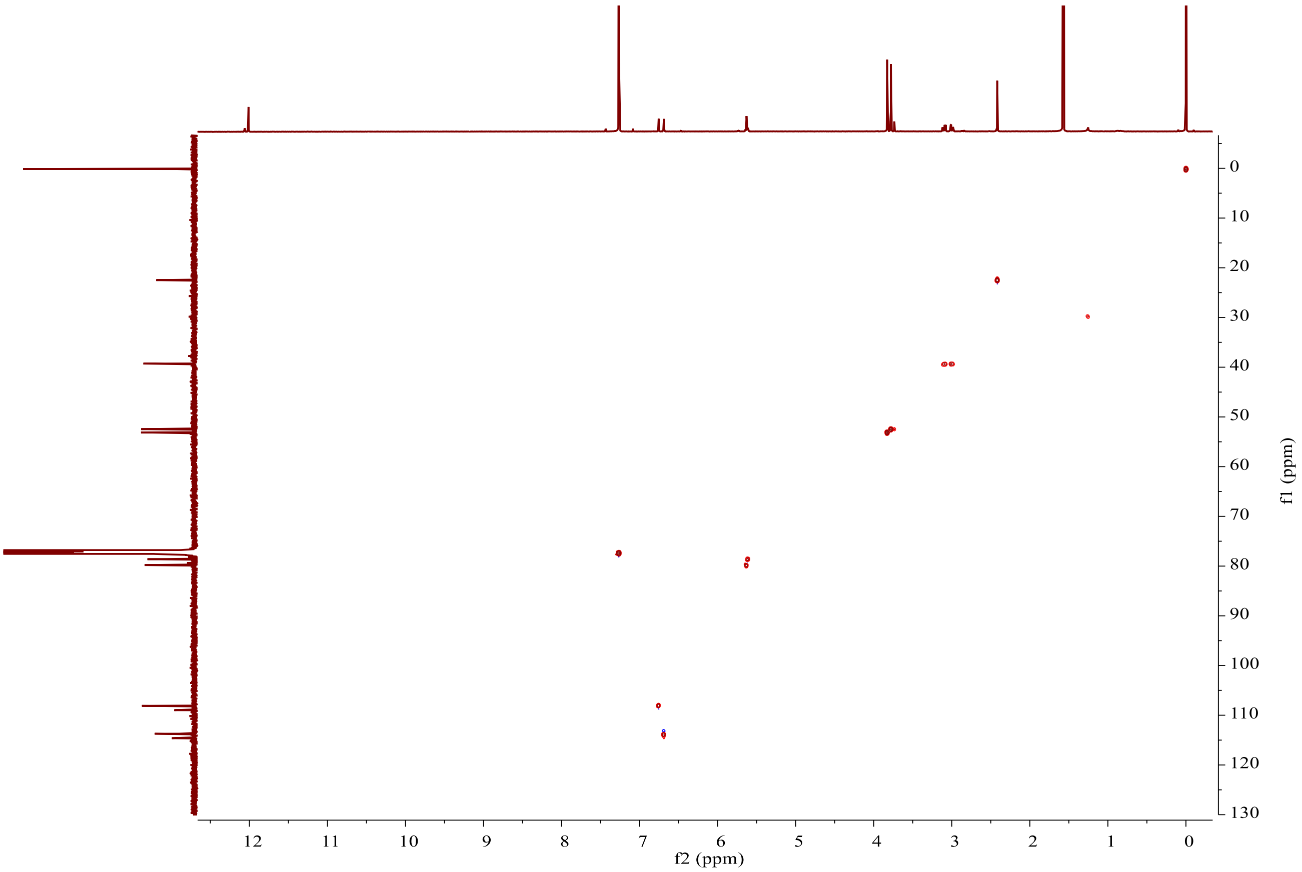

HMBC spectrum

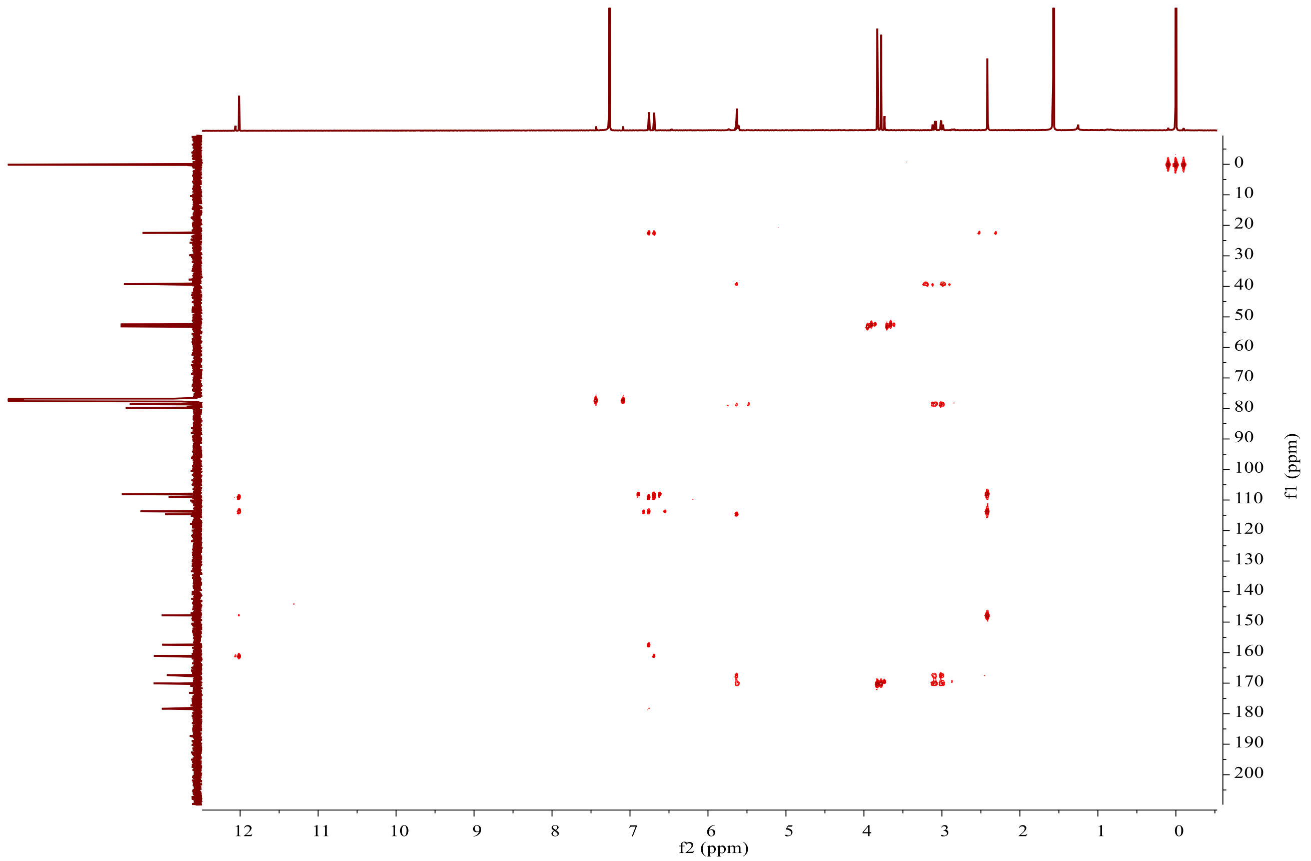

^1H-^1H COSY spectrum

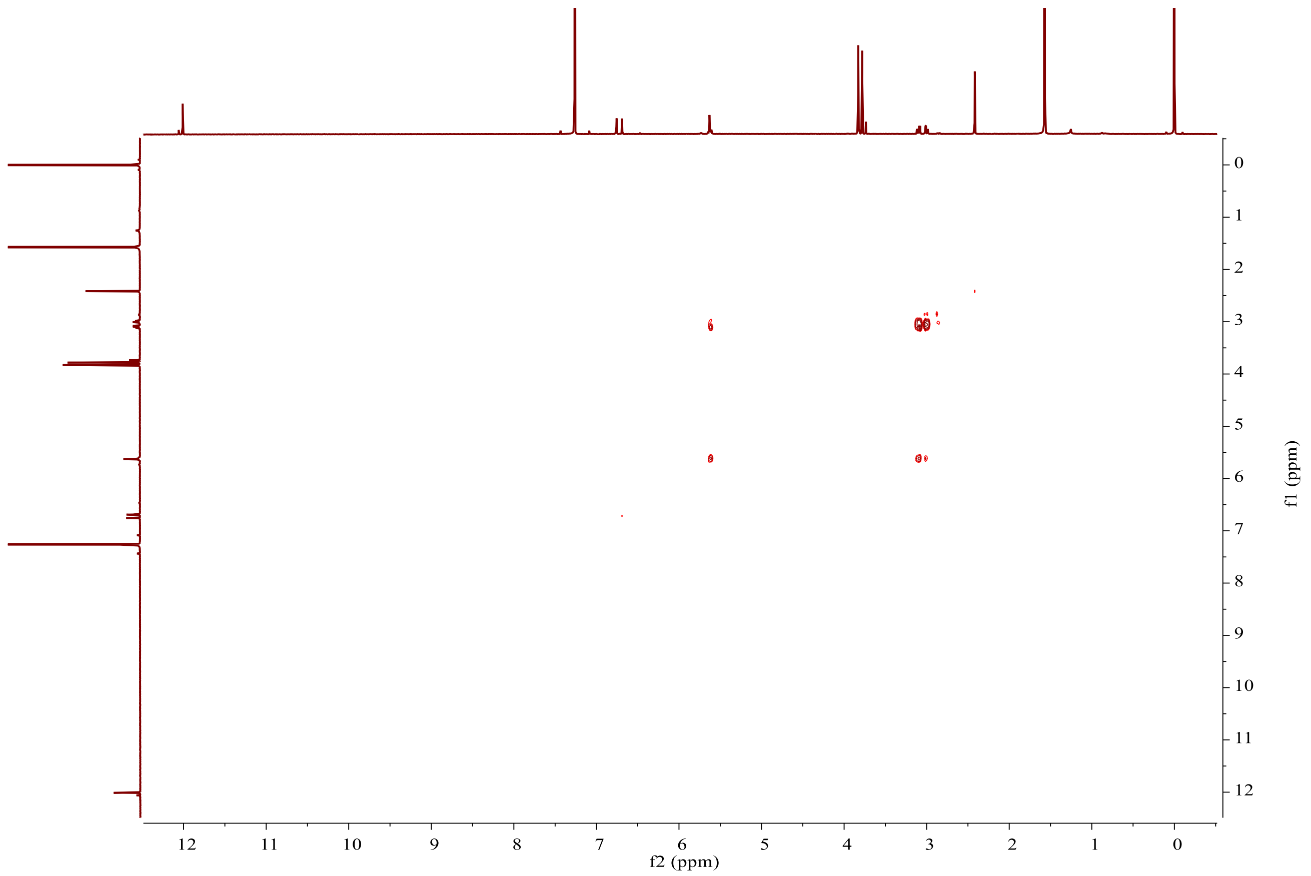

ROESY spectrum

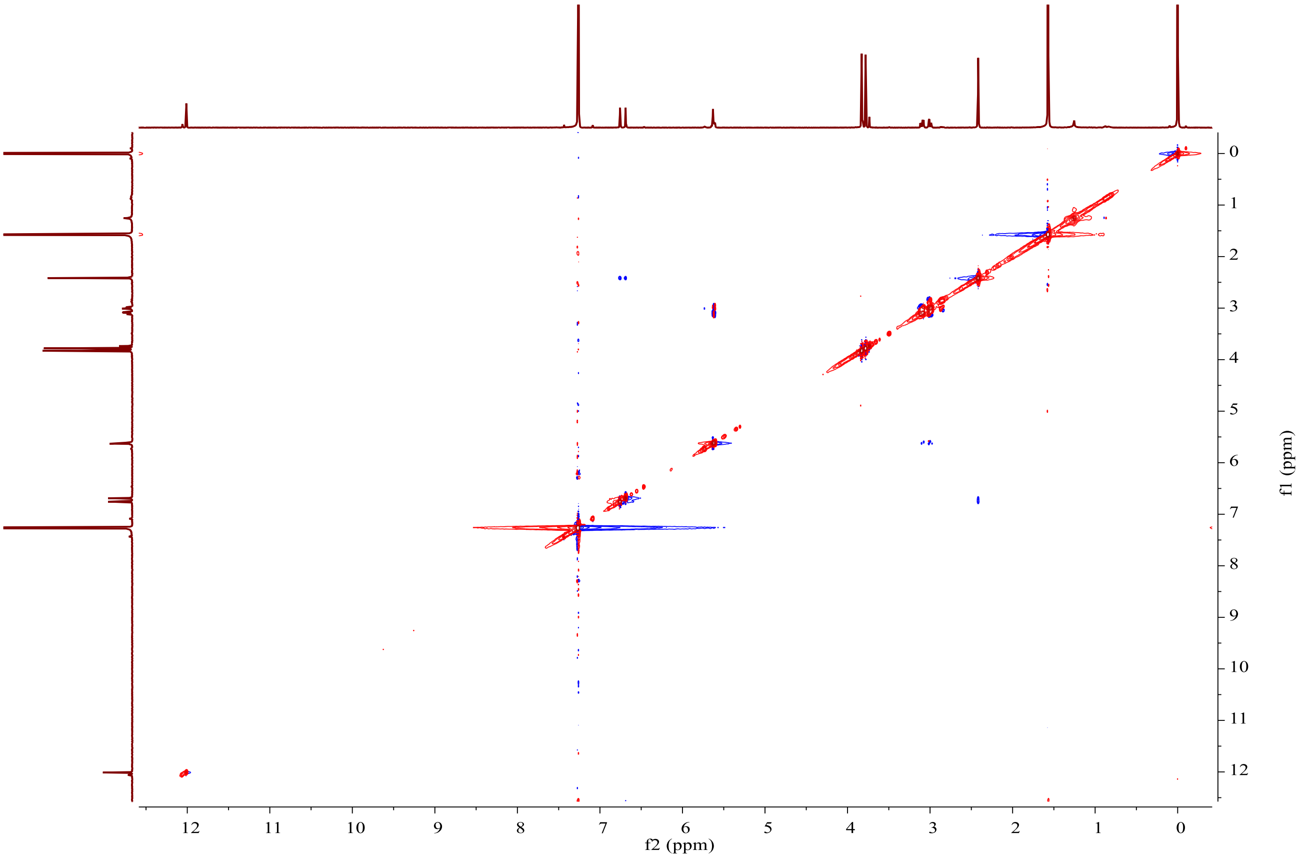

HRESIMS

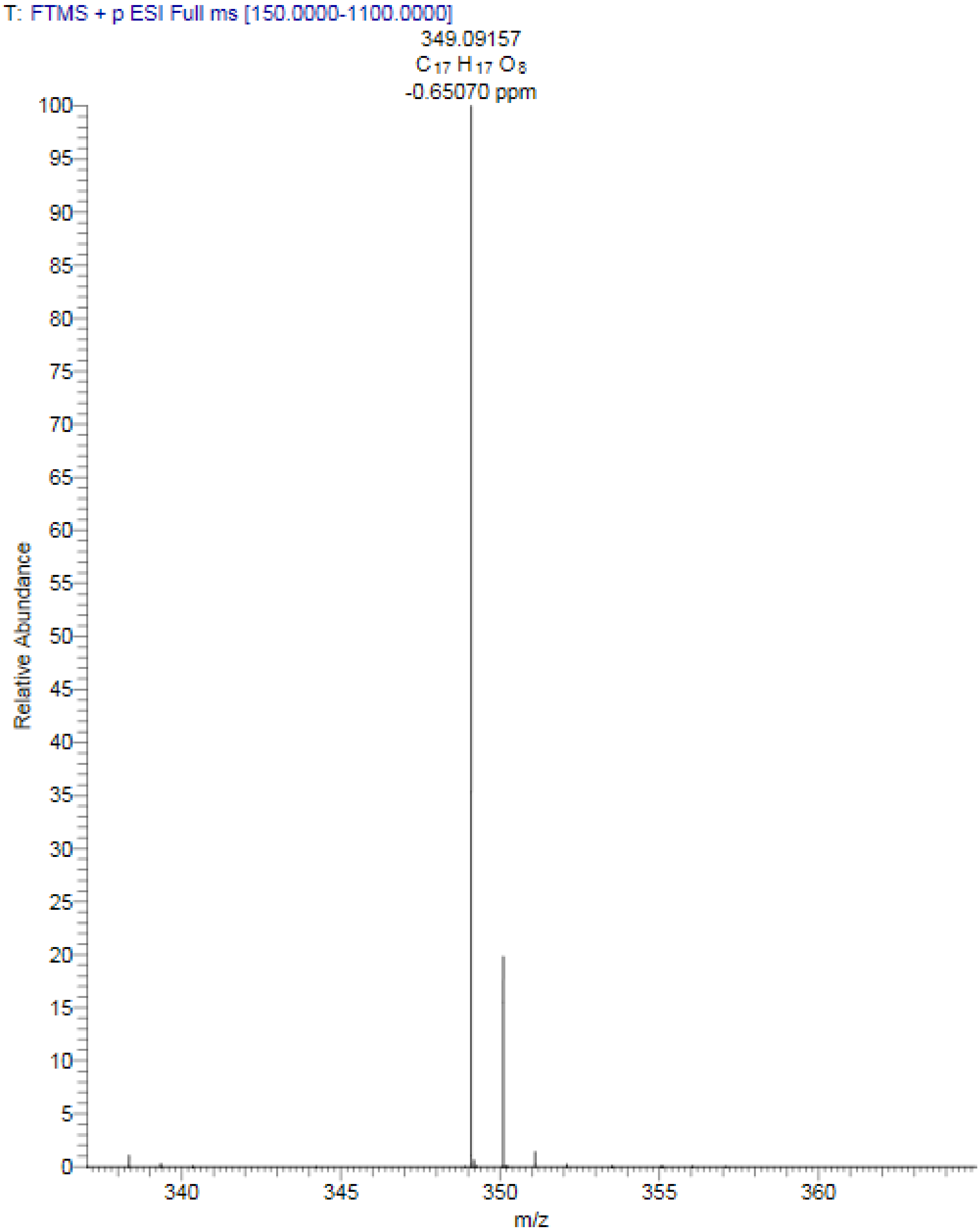
T: FTMS + p ESI Full ms [150.0000-1100.0000]
349.09157
C 17 H 17 O 8
-0.65070 ppm
Relative Abundance
m/z
100
95
90
85
80
75
70
65
60
55
50
45
40
35
30
25
20
15
10
5
0
340
345
350
355
360

CD spectra

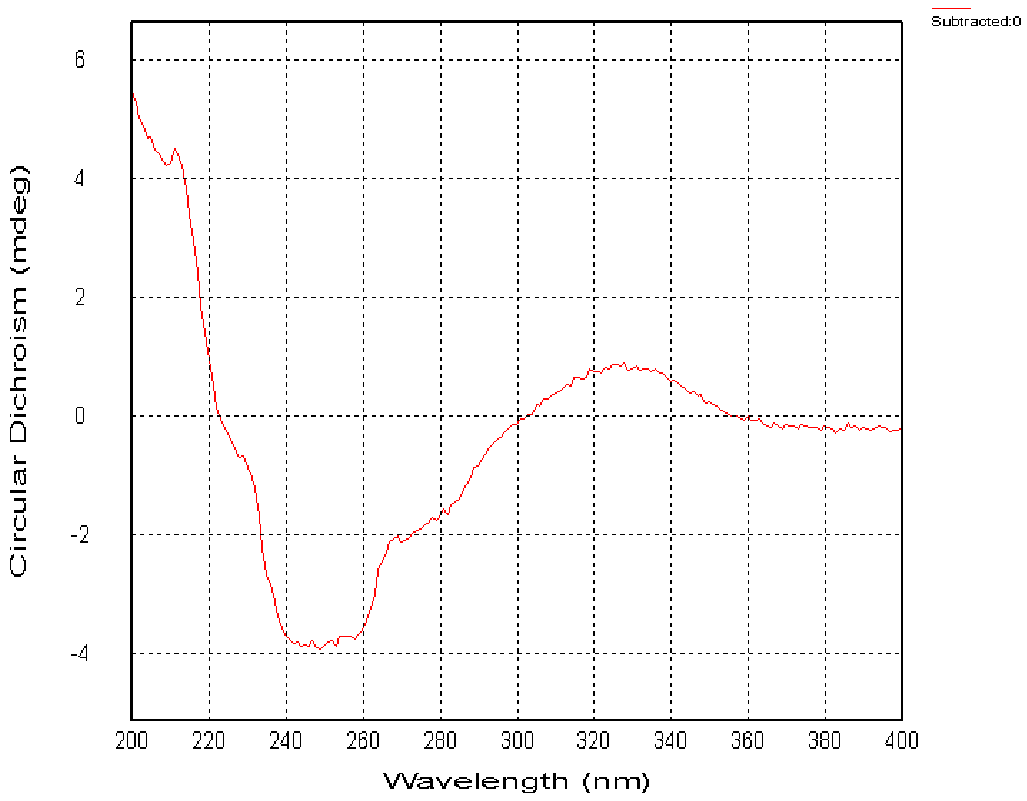

Subtracted:0
Circular Dichroism (mdeg)
6
4
2
0
-2
-4
200
220
240
260
280
300
320
340
360
380
400
Wavelength (nm)

S1.12 NMR, HRESIMS and CD spectra of bipolarithone C (12)

^{1}H NMR spectrum

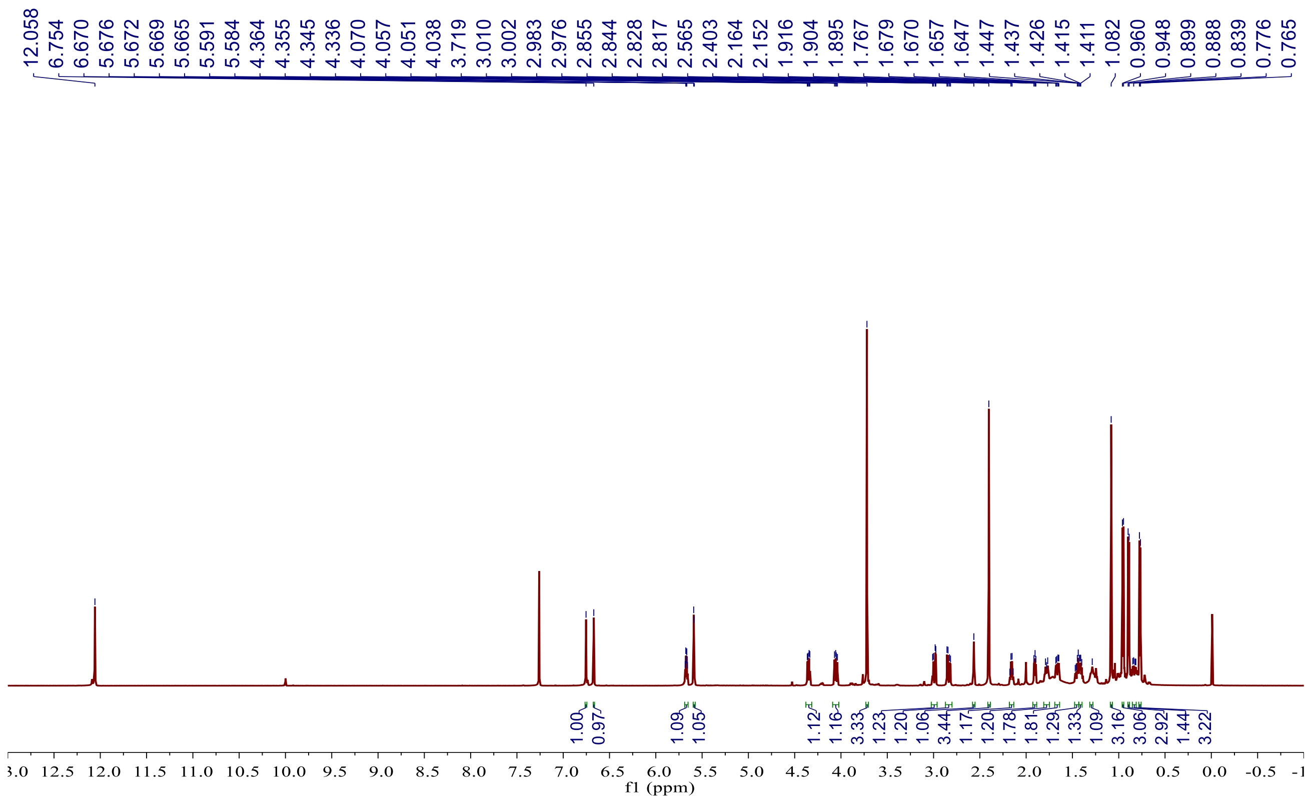

^{13}C NMR and DEPT spectra

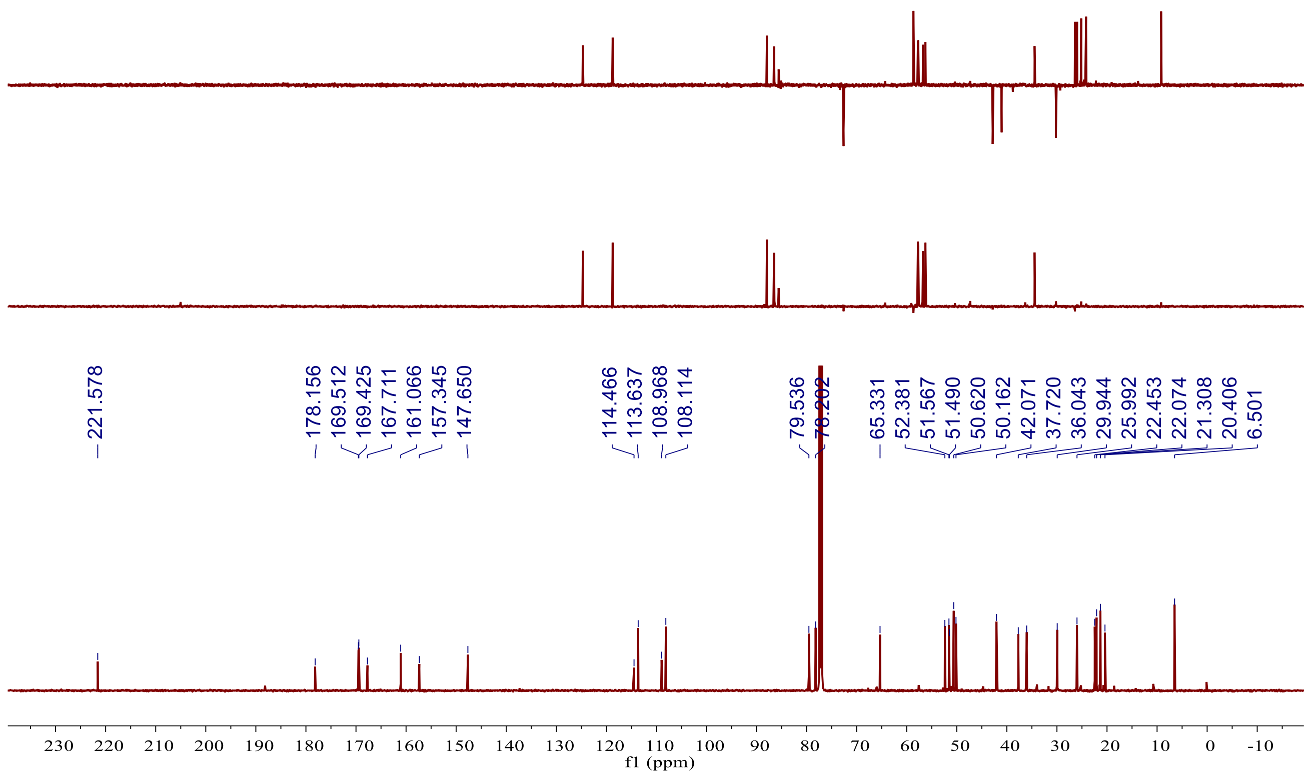

HSQC spectrum

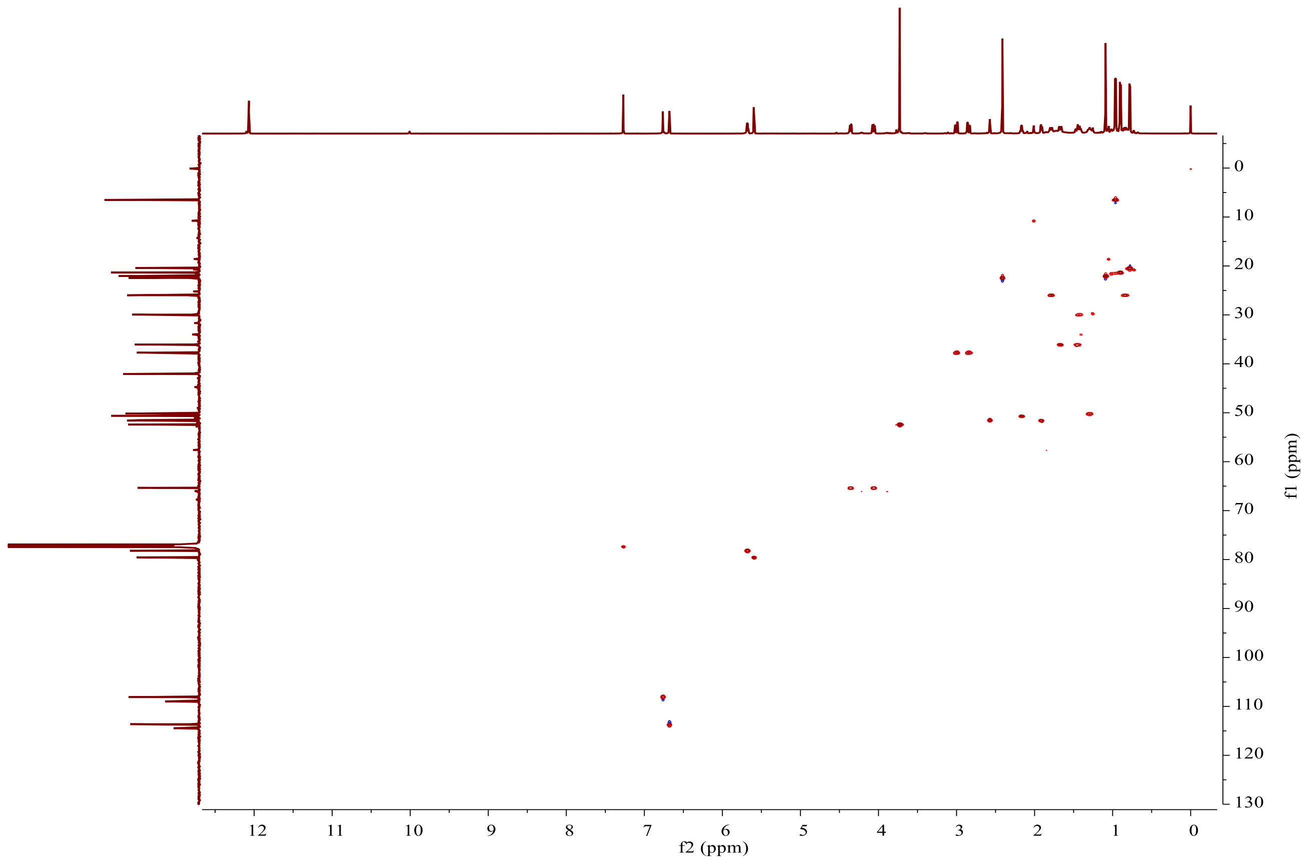

HMBC spectrum

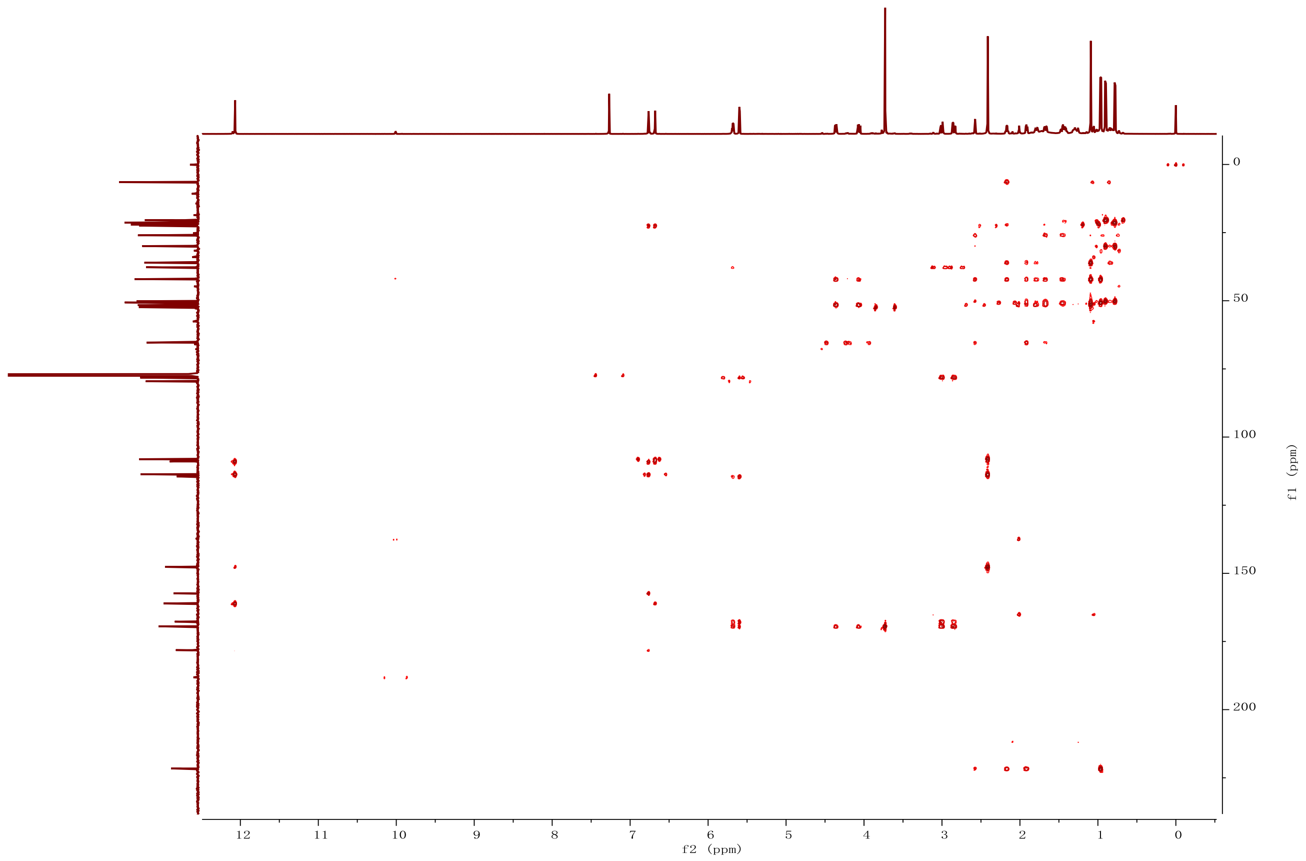

^{1}H-^{1}H COSY spectrum

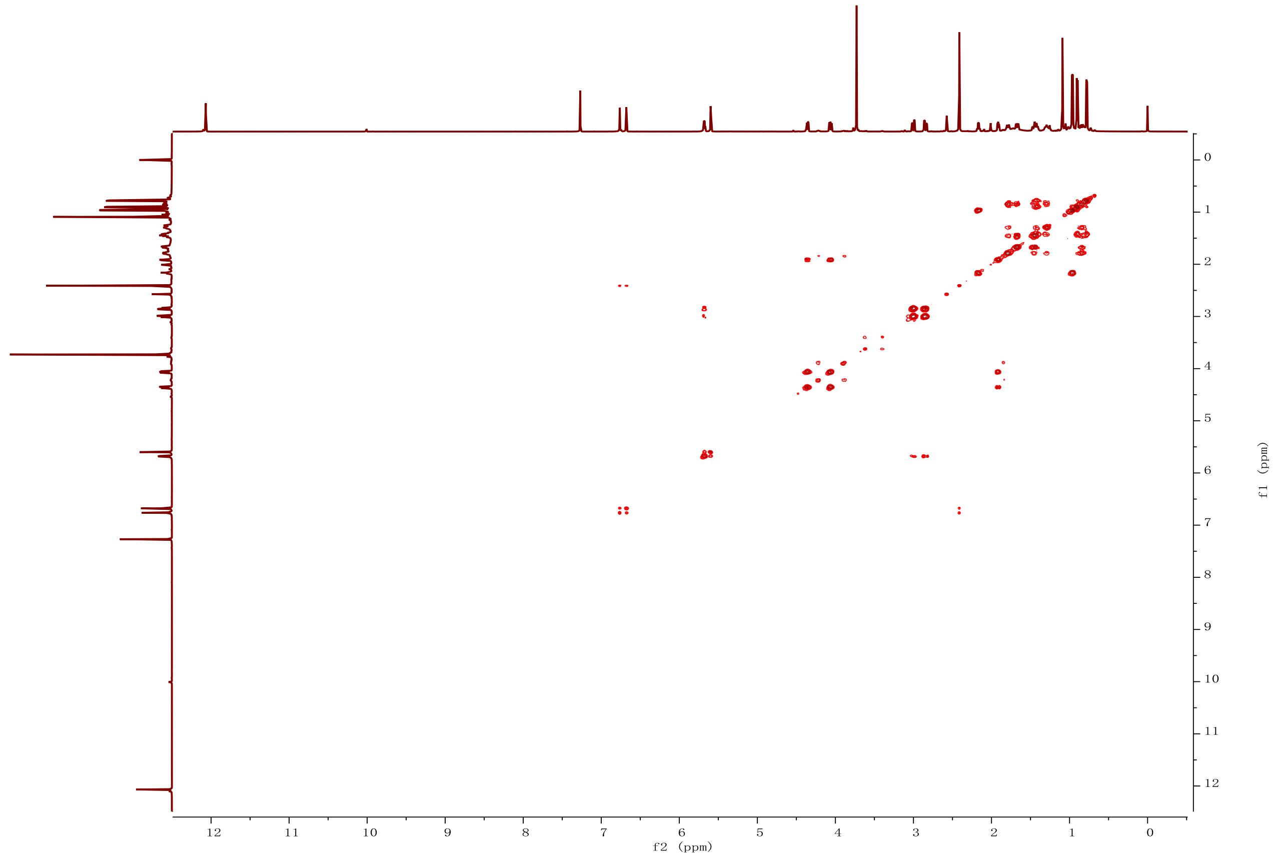

ROESY spectrum

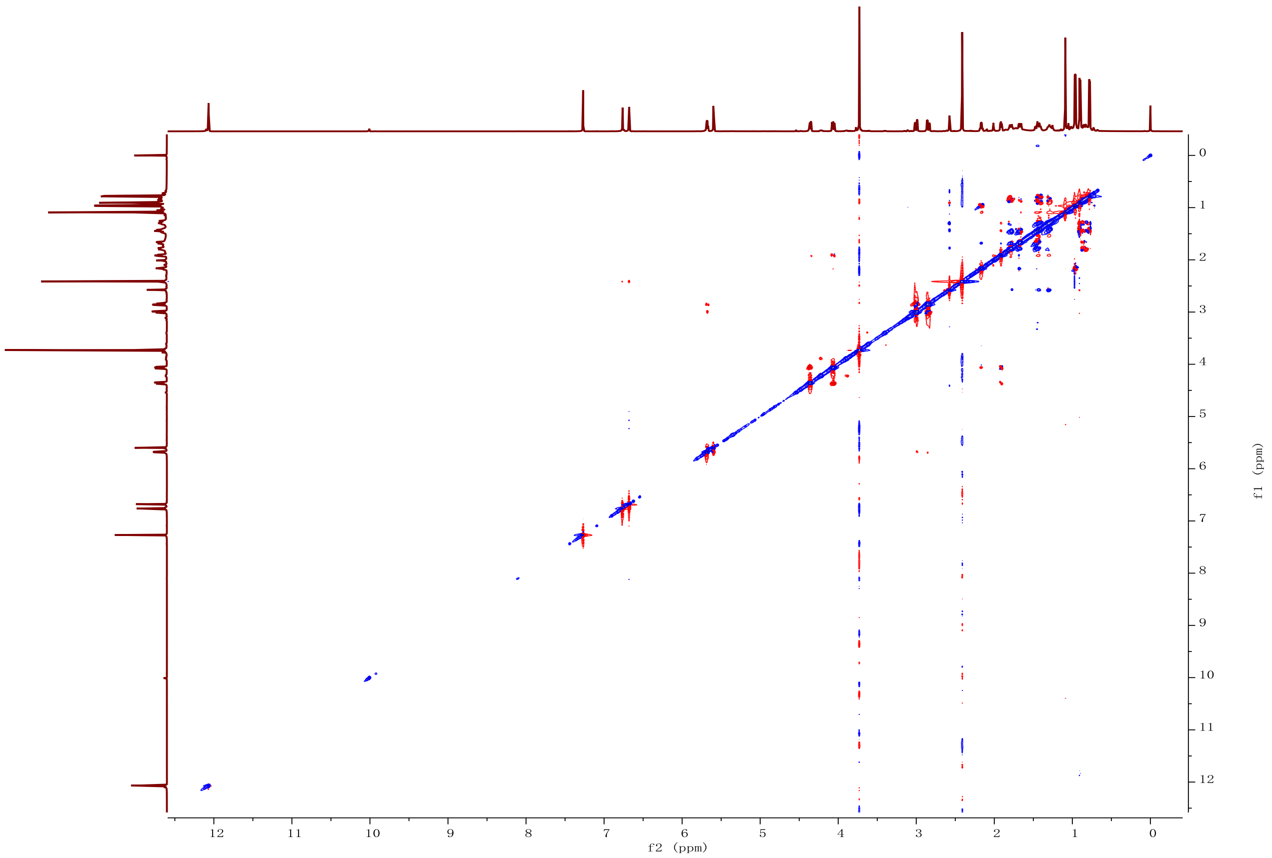

HRESIMS

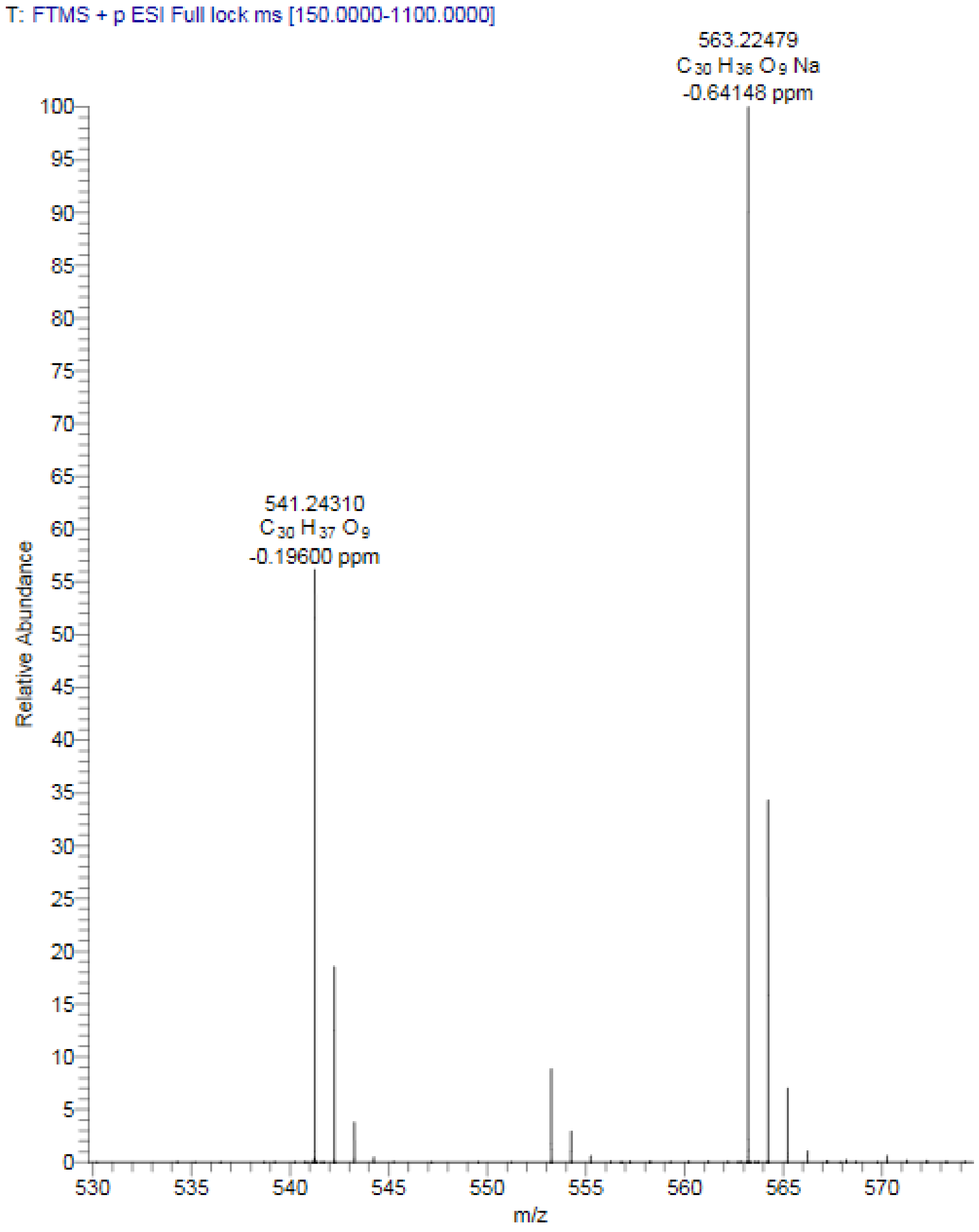
T: FTMS + p ESI Full lock ms [150.0000-1100.0000]
563.22479
C 30 H 36 O 9 Na
-0.64148 ppm
541.24310
C 30 H 37 O 9
-0.19600 ppm
Relative Abundance
m/z

CD spectra

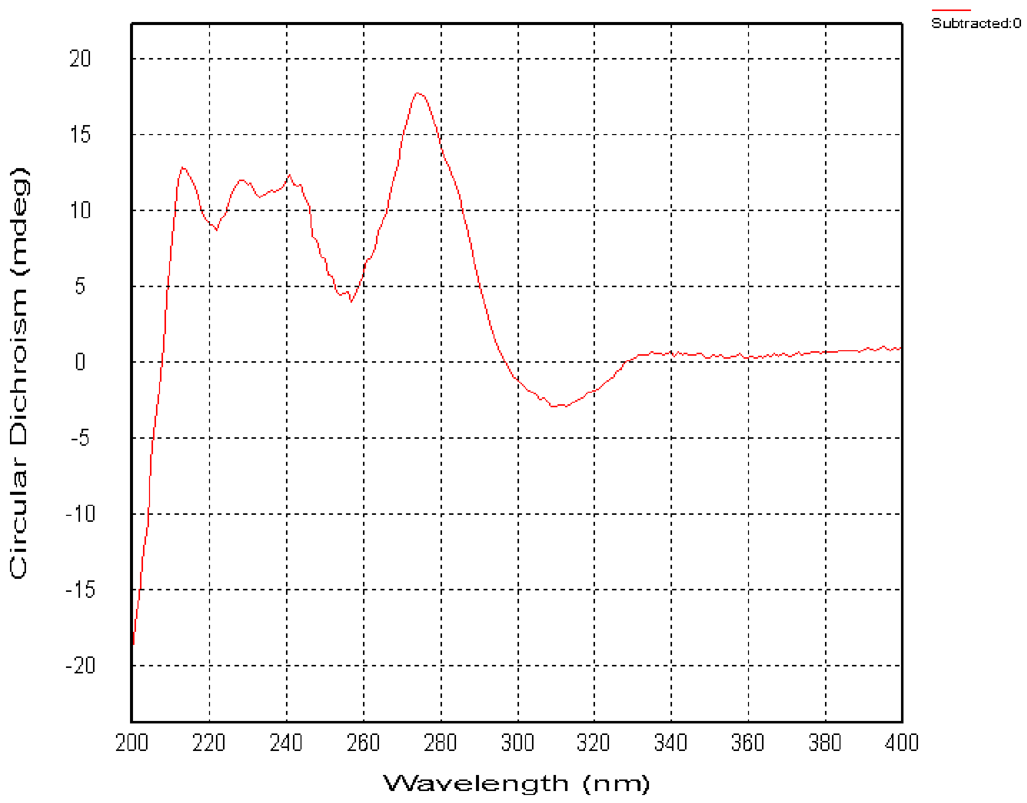

Subtracted:0
20
15
10
5
0
-5
-10
-15
-20
Circular Dichroism (mdeg)
200
220
240
260
280
300
320
340
360
380
400
Wavelength (nm)

S1.13 NMR, HRESIMS and CD spectra of bipolarithone D (13)

^{1}H NMR spectrum

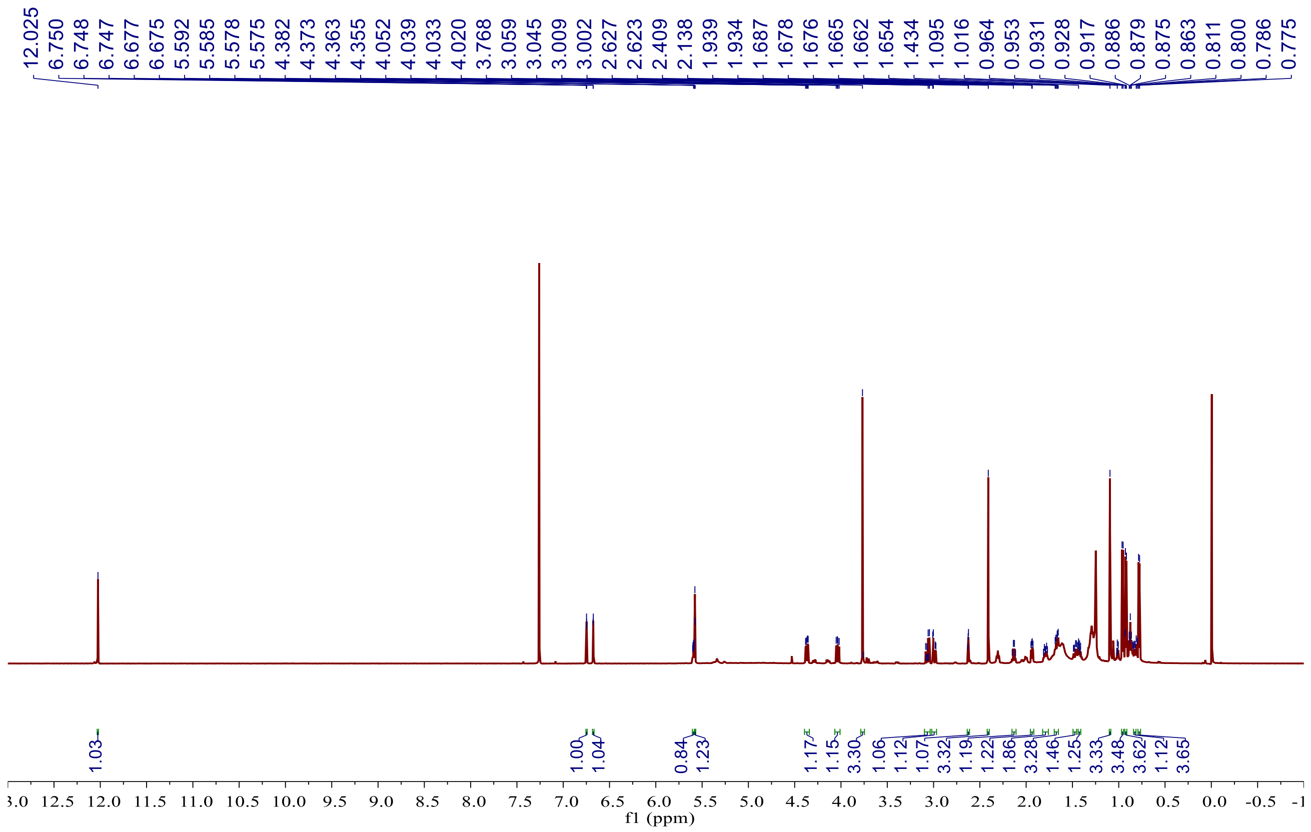

^{13}C NMR and DEPT spectra

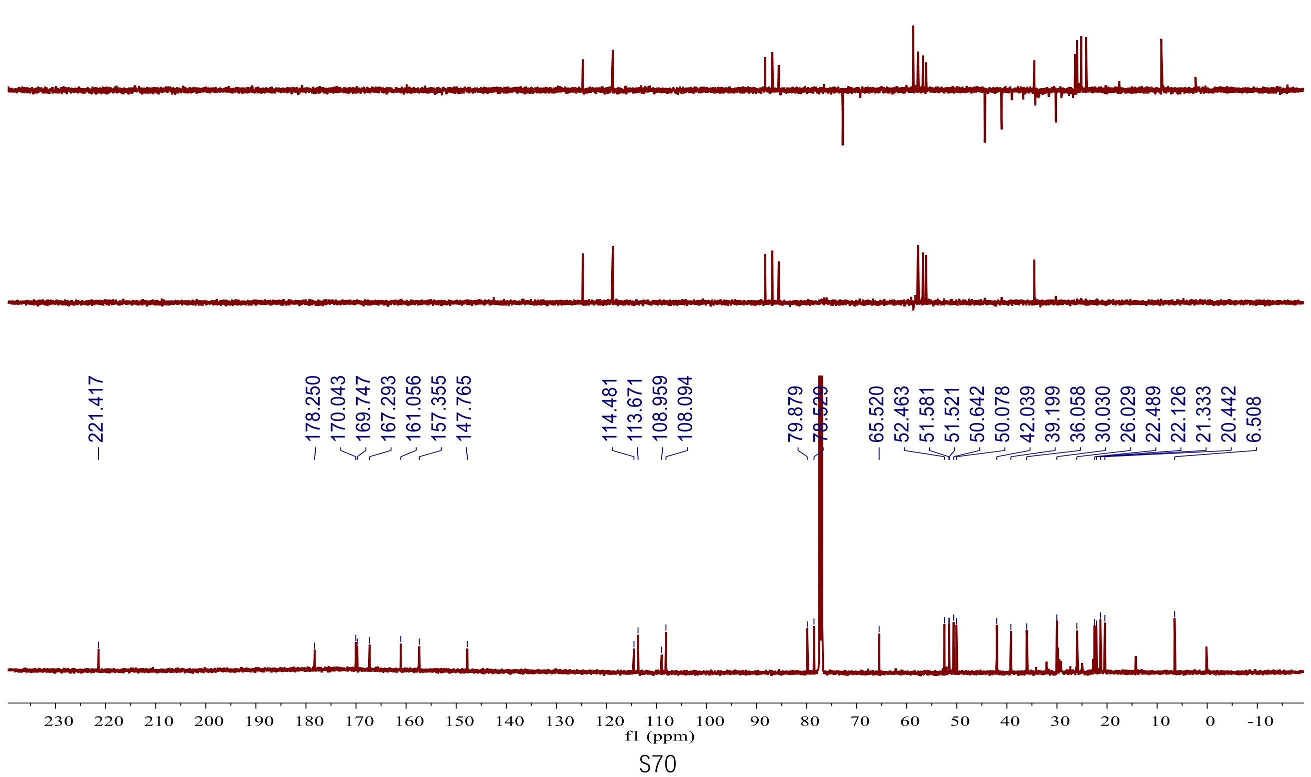

HSQC spectrum

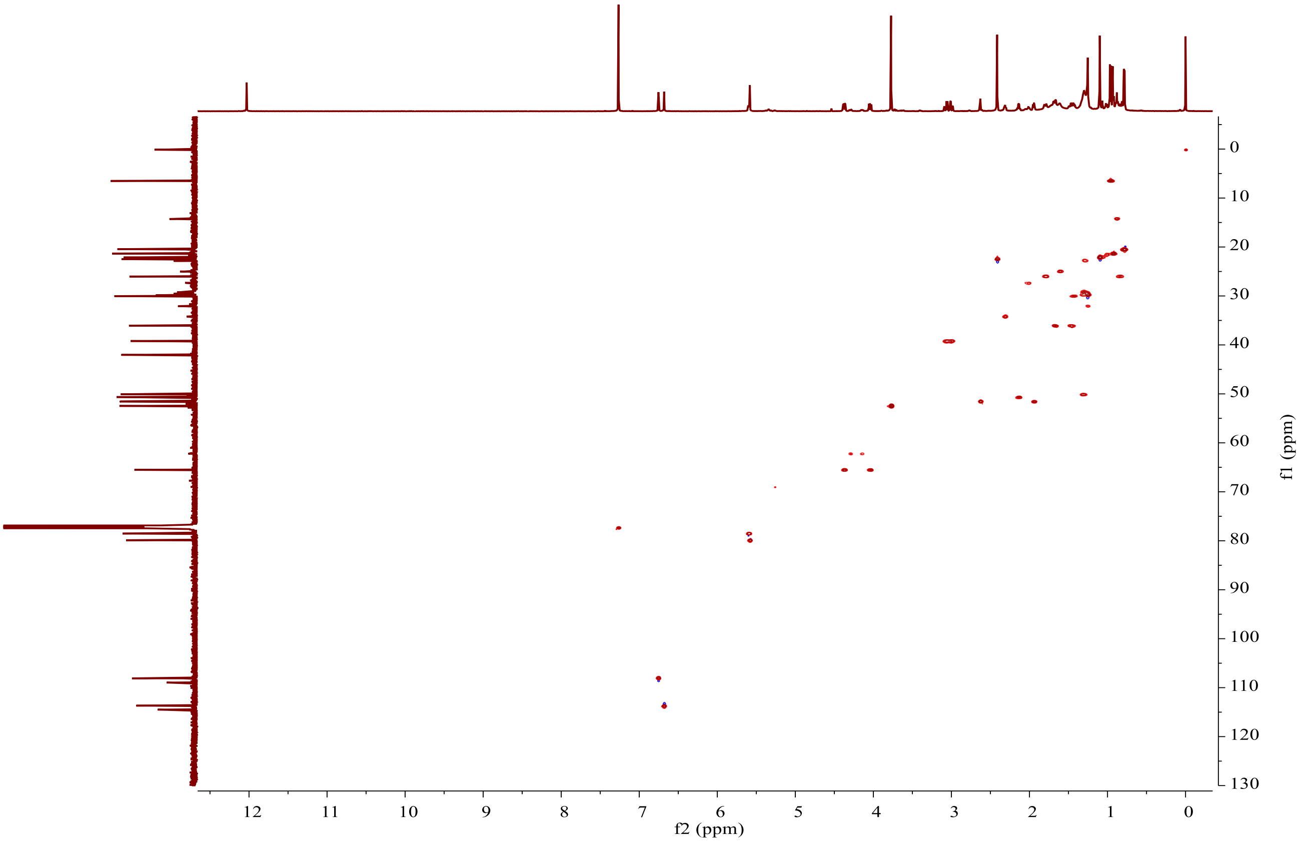

HMBC spectrum

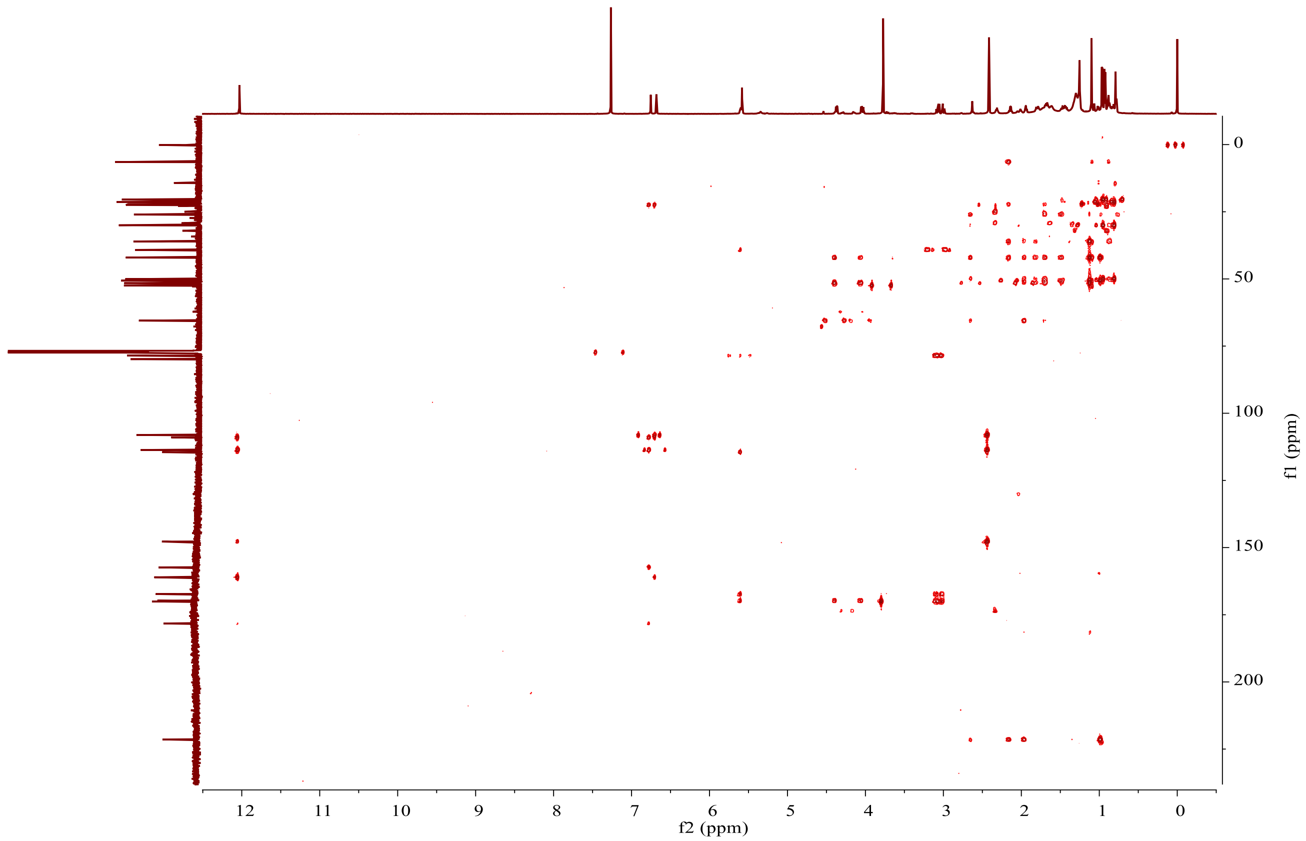

^{1}H-^{1}H COSY spectrum

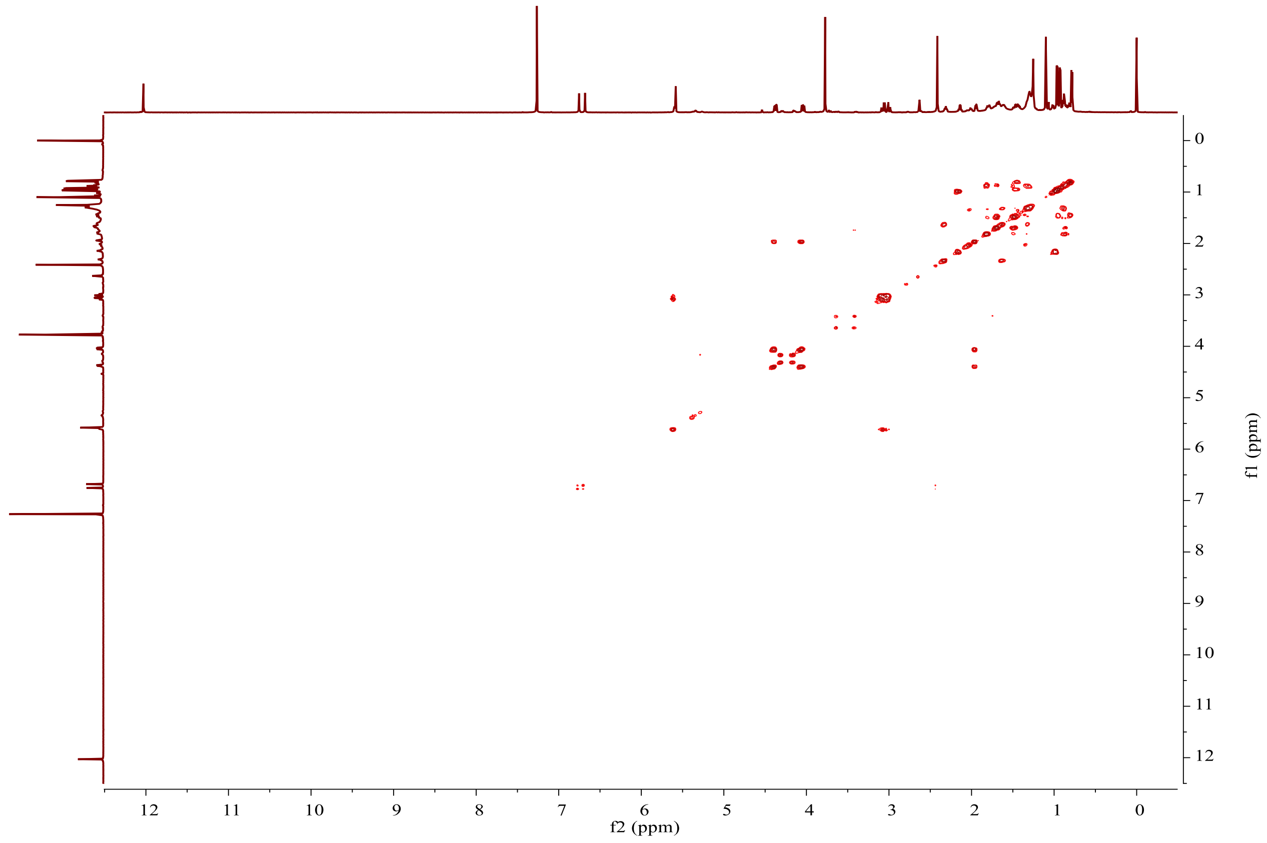

ROESY spectrum

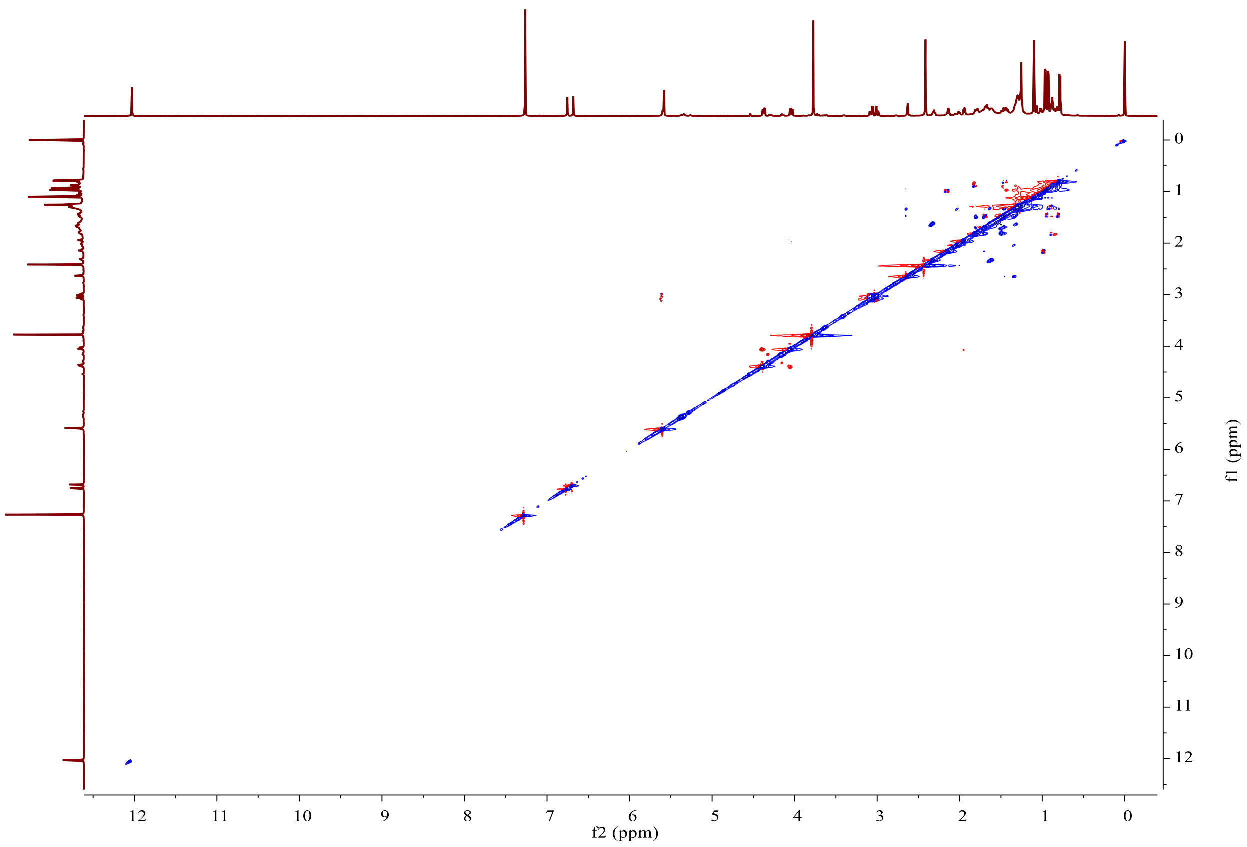

HRESIMS

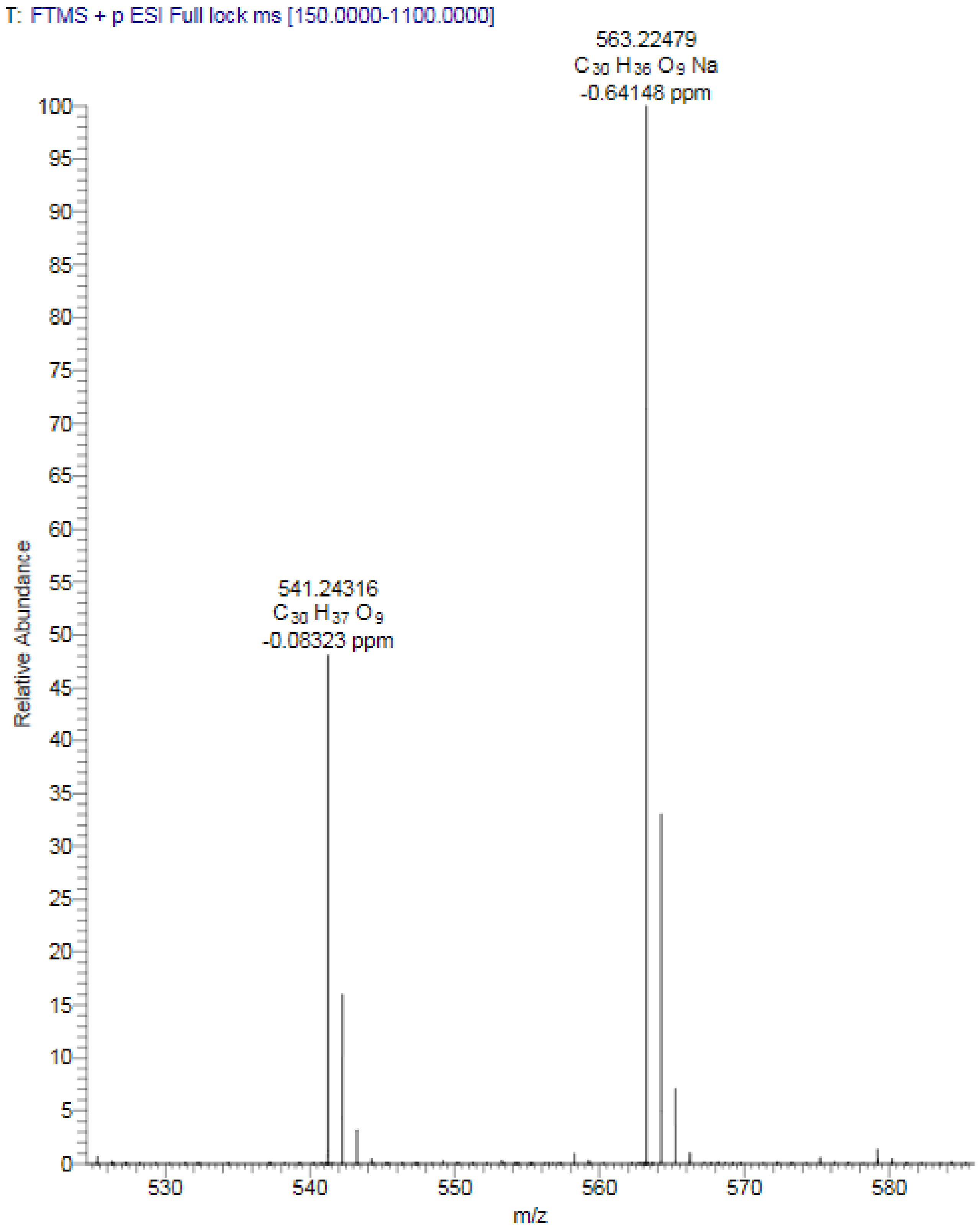

T: FTMS + p ESI Full lock ms [150.0000-1100.0000]
563.22479
C 30 H 36 O 9 Na
-0.64148 ppm
541.24316
C 30 H 37 O 9
-0.08323 ppm
Relative Abundance
m/z

CD spectra

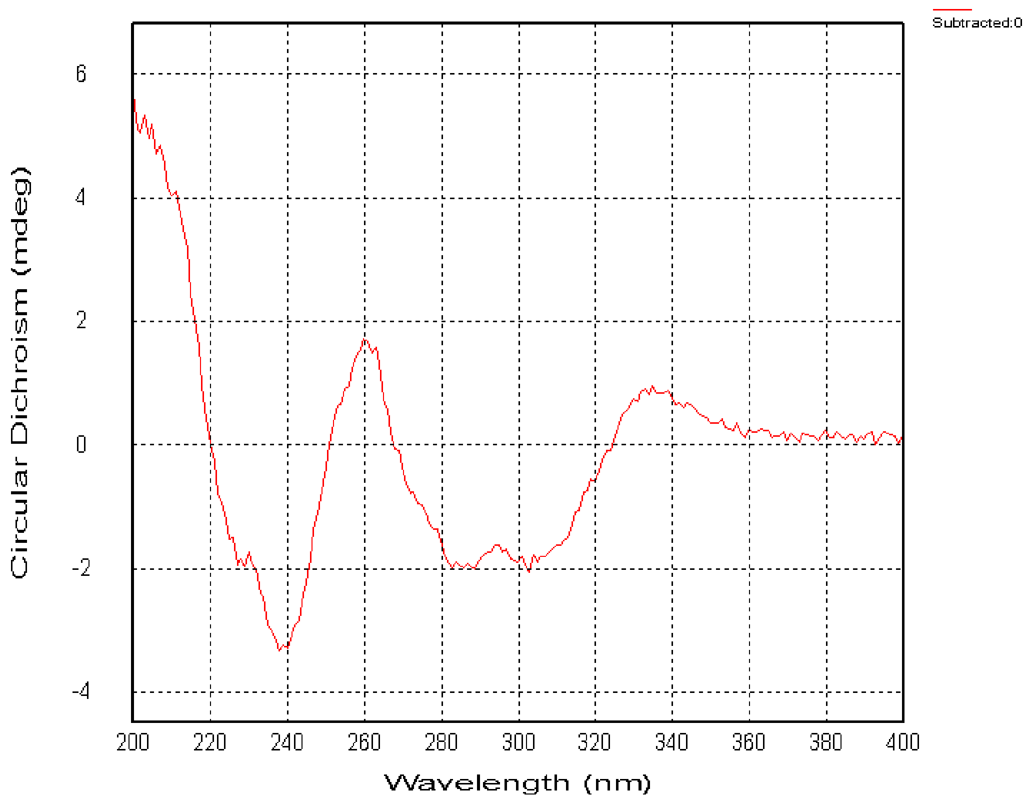
Subtracted:0
Circular Dichroism (mdeg)
6
4
2
0
-2
-4
200
220
240
260
280
300
320
340
360
380
400
Wavelength (nm)

Sections S2. Computational details

S2.1. Computational details for bipolarisorokin C (3) (ECD)

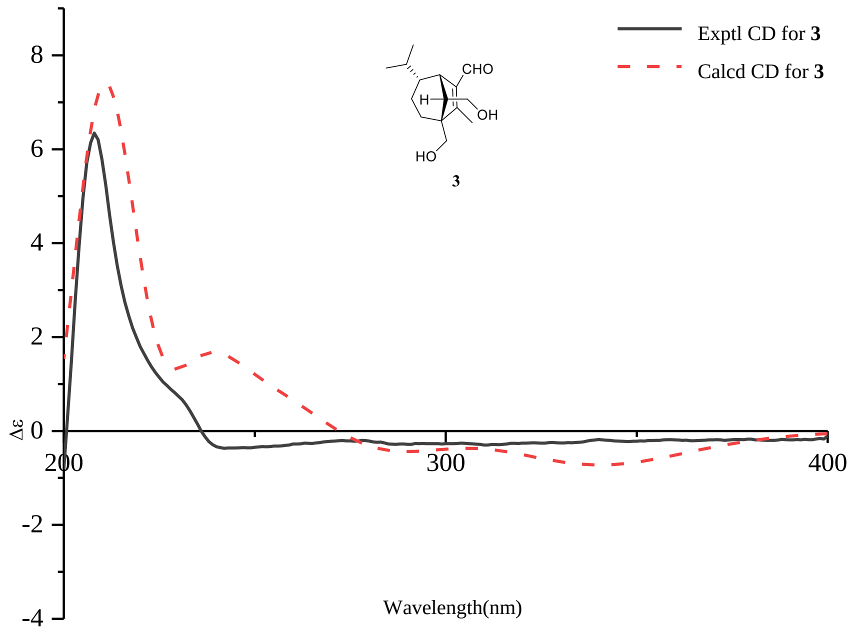

Figure S1. Calculated ECD spectra for **3** at the B3LYP/6-311G(d) level in methanol with IEFPCM model (σ = 0.35 eV, UV shift 7 nm). Experimental CD spectra of **3** (black line) in MeOH.

Table S1. Energy analysis for conformers of **3A~3F** at B3LYP/6-31G(d) level in the gas phase

Species	*E′*=*E*+*ZPE*	*E*	*H*	*G*	*ΔG*	*ΔE*(kcal/mol)	*PE*%
**3A**	-811.401389	-811.382734	-811.38179	-811.445976	0.002106	1.321535	6.80%
**3B**	-811.403156	-811.384823	-811.383879	-811.447234	0.000848	0.532128	25.79%
**3C**	-811.394306	-811.375171	-811.374227	-811.439445	0.008637	5.4198	0.01%
**3D**	-811.400708	-811.382174	-811.38123	-811.445153	0.002929	1.837975	2.84%
**3E**	-811.403984	-811.385629	-811.384685	-811.448082	0	0	63.34%
**3F**	-811.400129	-811.381683	-811.380739	-811.444362	0.00372	2.334335	1.23%

E, *E′*, *H*, *G*: total energy, total energy with zero point energy (*ZPE*), enthalpy, and Gibbs free energy

Figure S2. Main conformers of **3** in ECD

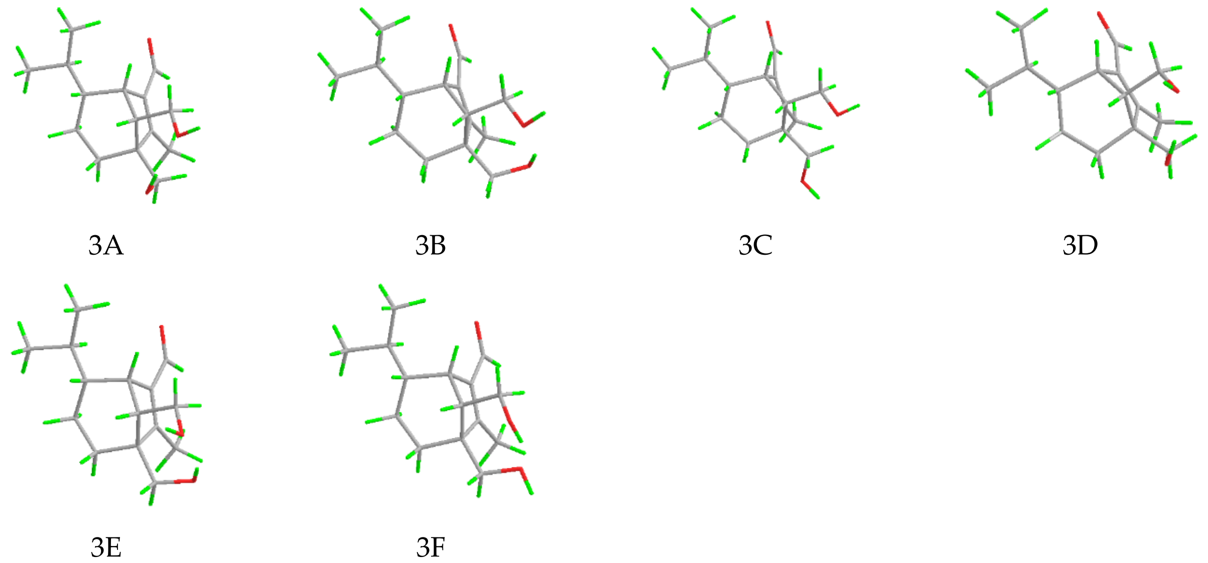

S2.2. Computational details for bipolarisorokin G (7) (ECD)

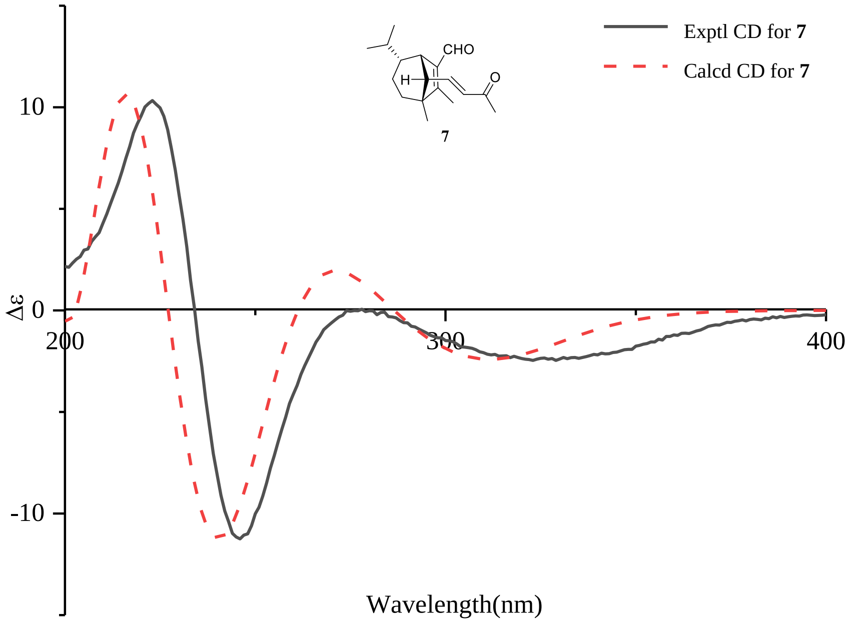

Figure S3. Calculated ECD spectra for **7** at the B3LYP/6-311G(d) level in methanol with IEFPCM model (σ = 0.30 eV, UV shift -**21** nm). Experimental CD spectra of **7** (black line) in MeOH.

Table S2. Energy analysis for conformers of **7A~7K** at B3LYP/6-31G(d) level in the gas phase

Species	*E′*=*E*+*ZPE*	*E*	*H*	*G*	*ΔG*	*ΔE*(kcal/mol)	*PE*%
**7A**	-851.689303	-851.667934	-851.66699	-851.738756	0.000753	0.472515	23.63%
**7B**	-851.689337	-851.668042	-851.667098	-851.738542	0.000967	0.606802	18.83%
**7C**	-851.689897	-851.668478	-851.667534	-851.739509	0	0	52.48%
**7D**	-851.687229	-851.66598	-851.665036	-851.736267	0.003242	2.034386	1.69%
**7E**	-851.685214	-851.663817	-851.662873	-851.735376	0.004133	2.593497	0.66%
**7F**	-851.684986	-851.663557	-851.662612	-851.734821	0.004688	2.941765	0.36%
**7G**	-851.685209	-851.663911	-851.662967	-851.734125	0.005384	3.378511	0.17%
**7H**	-851.686185	-851.665011	-851.664067	-851.73516	0.004349	2.729039	0.52%
**7I**	-851.685802	-851.664542	-851.663598	-851.73505	0.004459	2.798065	0.47%
**7J**	-851.685847	-851.664456	-851.663512	-851.735092	0.004417	2.771709	0.49%
**7K**	-851.685504	-851.664035	-851.663091	-851.735425	0.004084	2.562749	0.69%

E, *E'*, *H*, *G*: total energy, total energy with zero point energy (*ZPE*), enthalpy, and Gibbs free energy

Figure S4. Main conformers of **7** in ECD

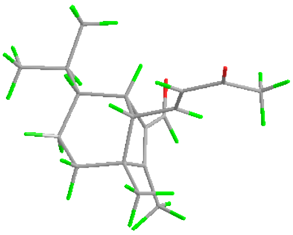

7A

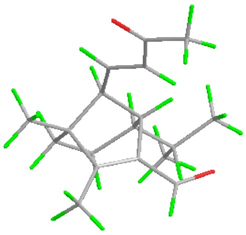

7B

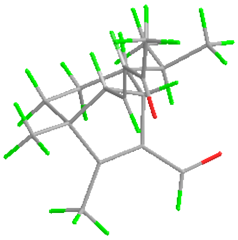

7C

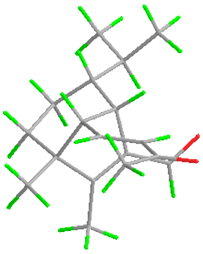

7D

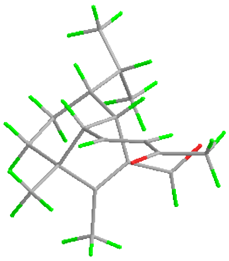

7E

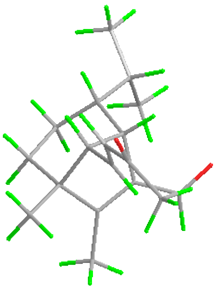

7F

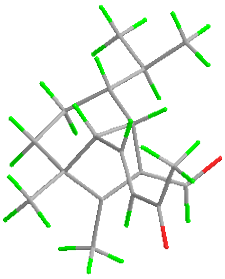

7G

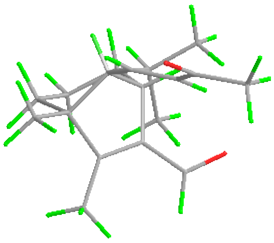

7H

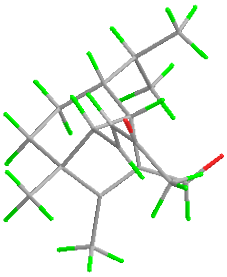

7I

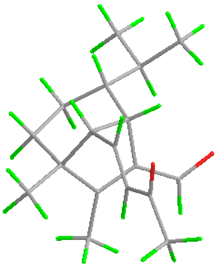

7J

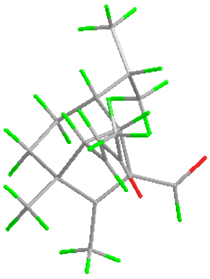

7K

S2.3. Computational details for bipolarithone A (10) (ECD)

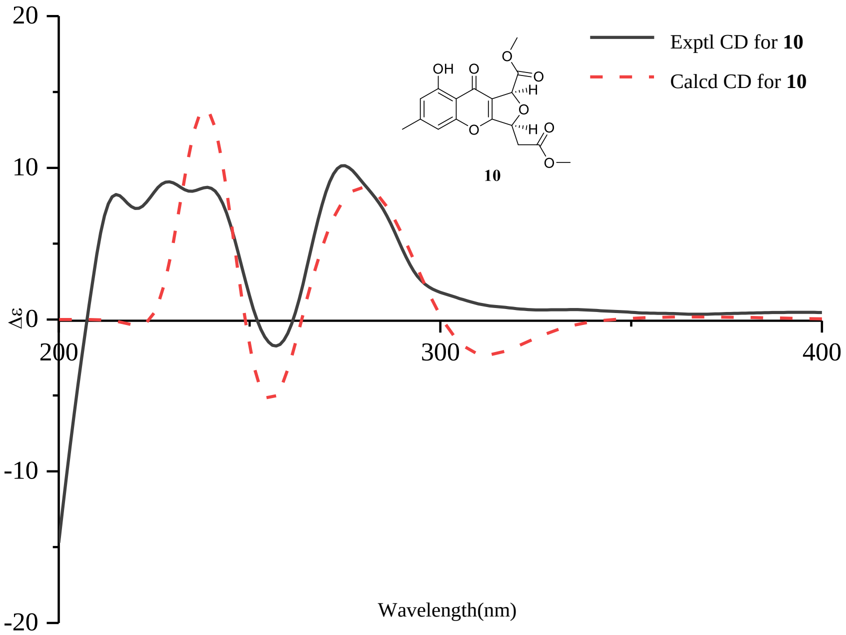

Figure S5. Calculated ECD spectra for **10** at the B3LYP/6-311G(d) level in methanol with IEFPCM model (σ = 0.32 eV, UV shift 29 nm). Experimental CD spectra of **10** (black line) in MeOH.

Table S3. Energy analysis for conformers of **10A~10J** at B3LYP/6-31G(d) level in the gas phase

Species	*E′*=*E*+*ZPE*	*E*	*H*	*G*	*ΔG*	*ΔE*(kcal/mol)	*PE*%
**10A**	-1259.018573	-1258.995248	-1258.994304	-1259.075493	0	0	37.35%
**10B**	-1259.018243	-1258.994946	-1258.994001	-1259.074519	0.000974	0.611194253	13.30%
**10C**	-1259.017827	-1258.994525	-1258.99358	-1259.073885	0.001608	1.009035276	6.79%
**10D**	-1259.016576	-1258.993274	-1258.99233	-1259.072975	0.002518	1.580068921	2.59%
**10E**	-1259.019921	-1258.996898	-1258.995954	-1259.074685	0.000808	0.507027676	15.86%
**10F**	-1259.019649	-1258.99674	-1258.995795	-1259.073383	0.00211	1.324045045	3.99%
**10G**	-1259.019922	-1258.996899	-1258.995955	-1259.074692	0.000801	0.50263511	15.98%
**10H**	-1259.016416	-1258.993081	-1258.992137	-1259.072682	0.002811	1.763929205	1.90%
**10I**	-1259.016685	-1258.993481	-1258.992537	-1259.07197	0.003523	2.210715969	0.89%
**10J**	-1259.016394	-1258.993049	-1258.992105	-1259.072351	0.003142	1.971634849	1.34%

E, *E′*, *H*, *G*: total energy, total energy with zero point energy (*ZPE*), enthalpy, and Gibbs free energy

Figure S6. Main conformers of **10** in ECD

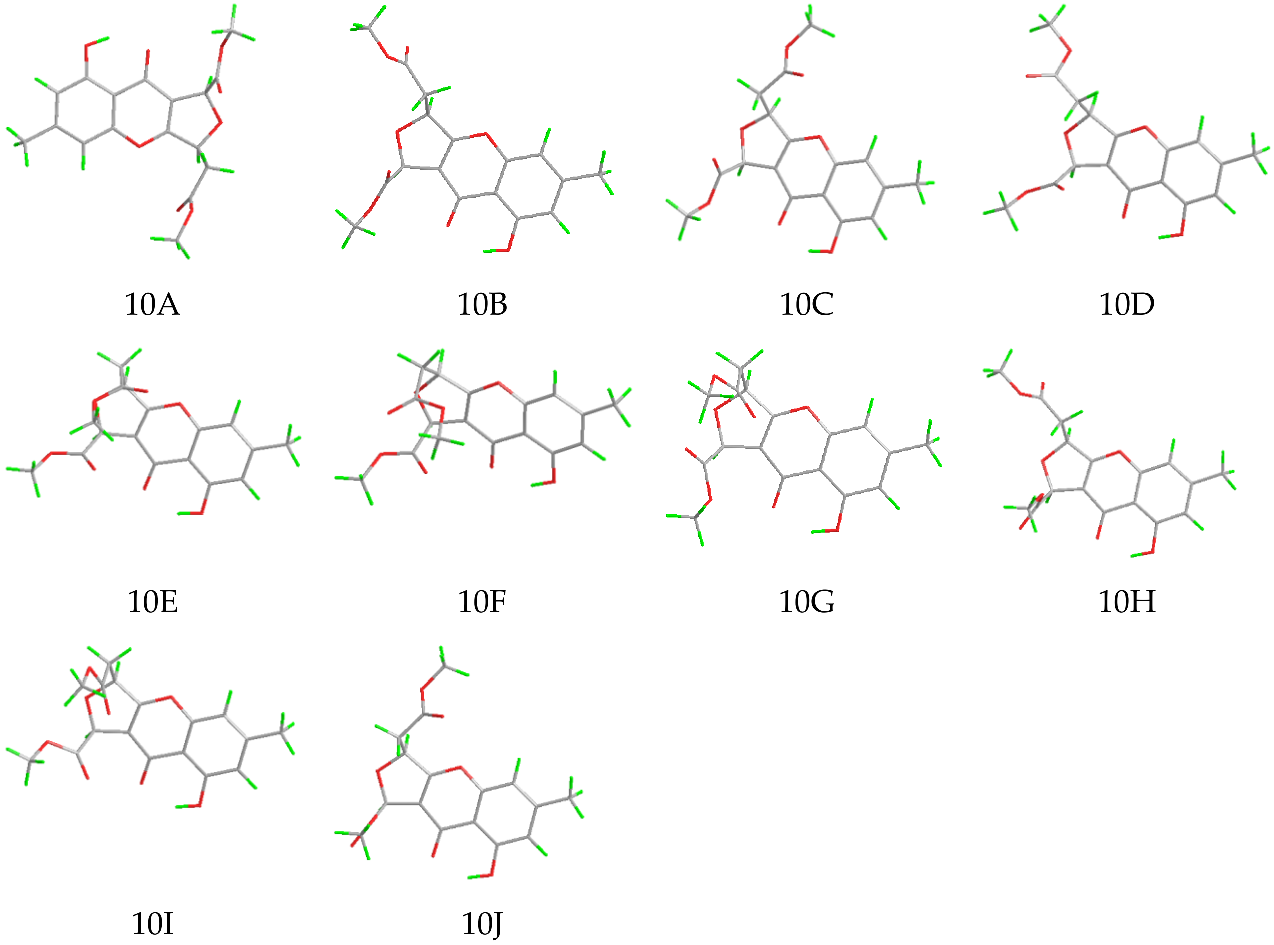

S2.4. Computational details for bipolarithone B (11) (ECD)

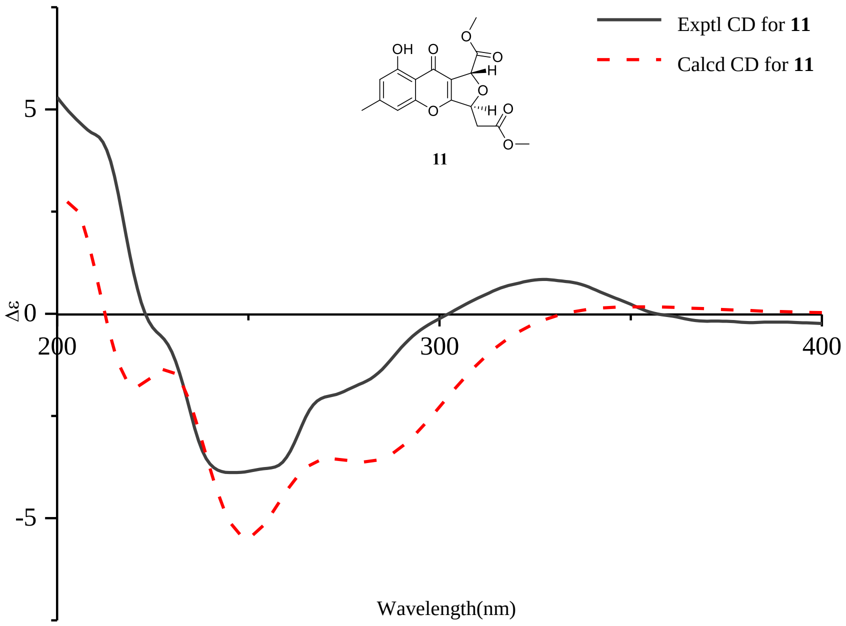

Figure S7. Calculated ECD spectra for **11** at the B3LYP/6-311G(d) level in methanol with IEFPCM model (σ = 0.38 eV, UV shift 2 nm). Experimental CD spectra of **11** (black line) in MeOH.

Table S4. Energy analysis for conformers of **11A~11K** at B3LYP/6-31G(d) level in the gas phase

Species	E'=E+ZPE	E	H	G	ΔG	ΔE(kcal/mol)	PE%
**11A**	-1259.0163	-1258.99289	-1258.991942	-1259.073522	0.000625	0.392193	16.46%
**11B**	-1259.0163	-1258.99288	-1258.991935	-1259.073547	0.0006	0.376506	16.90%
**11C**	-1259.0156	-1258.99216	-1258.991211	-1259.072575	0.001572	0.986445	6.03%
**11D**	-1259.0161	-1258.99272	-1258.991774	-1259.072775	0.001372	0.860943	7.46%
**11E**	-1259.0179	-1258.99463	-1258.993688	-1259.074147	0	0	31.92%
**11F**	-1259.0151	-1258.99163	-1258.990684	-1259.071946	0.002201	1.381148	3.10%
**11G**	-1259.0157	-1258.99224	-1258.991292	-1259.072508	0.001639	1.028488	5.62%
**11H**	-1259.0151	-1258.99173	-1258.990784	-1259.071808	0.002339	1.467745	2.68%
**11I**	-1259.0151	-1258.99172	-1258.990774	-1259.071517	0.00263	1.65035	1.97%
**11J**	-1259.0169	-1258.99356	-1258.992613	-1259.073068	0.001079	0.677083	10.17%
**11K**	-1259.0141	-1258.99062	-1258.989679	-1259.070653	0.003494	2.192518	0.79%

E, E', H, G: total energy, total energy with zero point energy (ZPE), enthalpy, and Gibbs free energy

Figure S8. Main conformers of **11** in ECD

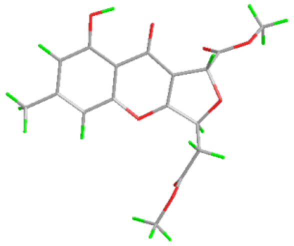

11A

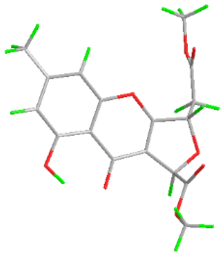

11B

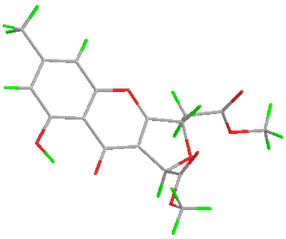

11C

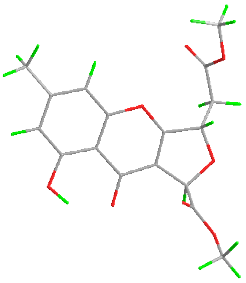

11D

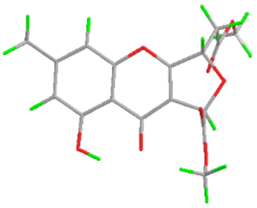

11E

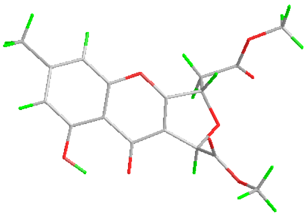

11F

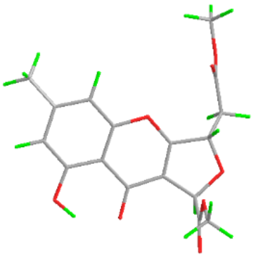

11G

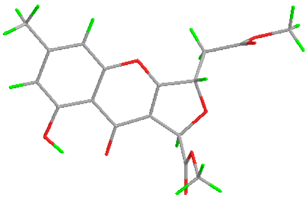

11H

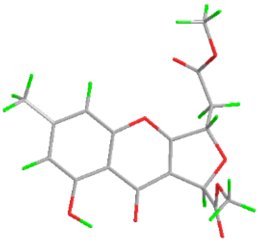

11I

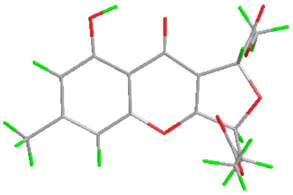

11J

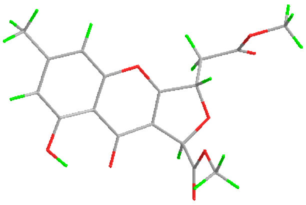

11K

S2.5. Computational details for bipolarithone C (12) (ECD)

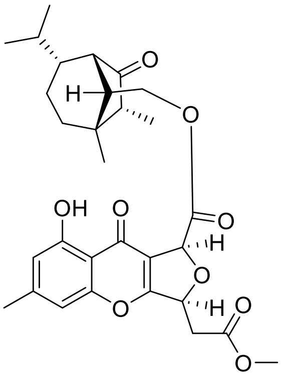

12a

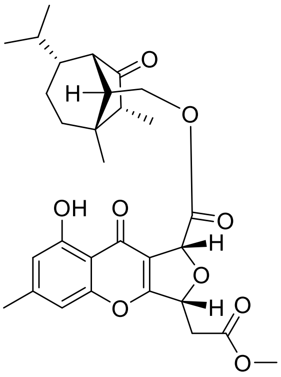

12b

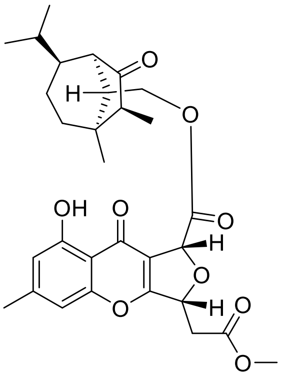

12c

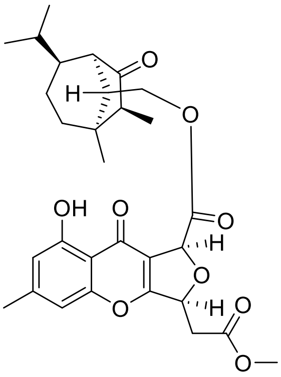

12d

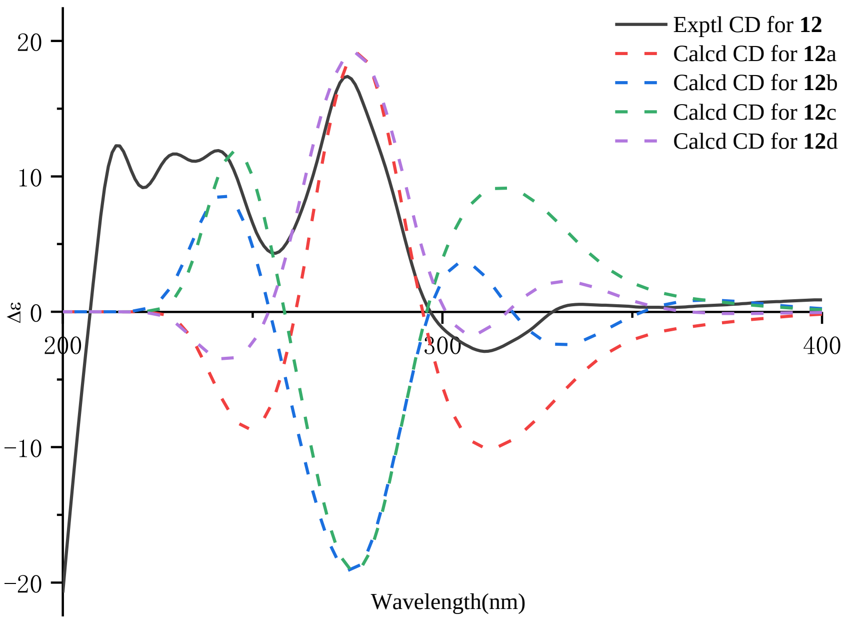

Figure S9. Calculated ECD spectra for **12a–12d** at the B3LYP/6-311G(d) level in methanol with IEFPCM model (σ = 0.30 eV, UV shift 26 nm). Experimental CD spectra of **12** (black line) in MeOH.

Table S5. Energy analysis for conformers of **12aA~12aK** at B3LYP/6-31G(d) level in the gas phase

Species	*E′=E+ZPE*	*E*	*H*	*G*	*ΔG*	*ΔE*(kcal/mol)	*PE*%
**12aA**	-1841.477567	-1841.440453	-1841.439509	-1841.547406	0.000796	0.499498	24.42%
**12aB**	-1841.480266	-1841.443482	-1841.442538	-1841.548202	0	0	56.78%
**12aC**	-1841.475594	-1841.438502	-1841.437558	-1841.545011	0.003191	2.002383	1.93%
**12aD**	-1841.469323	-1841.431763	-1841.430818	-1841.542825	0.005377	3.374119	0.19%
**12aE**	-1841.476806	-1841.439623	-1841.438679	-1841.546971	0.001231	0.772464	15.40%
**12aF**	-1841.473561	-1841.436386	-1841.435442	-1841.544096	0.004106	2.576554	0.73%
**12aG**	-1841.471611	-1841.43455	-1841.433605	-1841.541743	0.006459	4.053084	0.06%
**12aH**	-1841.468618	-1841.431083	-1841.430139	-1841.541659	0.006543	4.105795	0.06%
**12aI**	-1841.47161	-1841.434552	-1841.433608	-1841.541673	0.006529	4.09701	0.06%
**12aJ**	-1841.471135	-1841.434062	-1841.433118	-1841.541871	0.006331	3.972763	0.07%
**12aK**	-1841.474176	-1841.437091	-1841.436147	-1841.54328	0.004922	3.088602	0.31%

E, *E*′, *H*, *G*: total energy, total energy with zero point energy (*ZPE*), enthalpy, and Gibbs free energy

Figure S10. Main conformers of **12a** in ECD

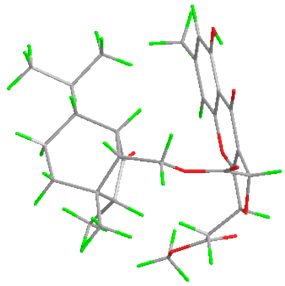

12aA

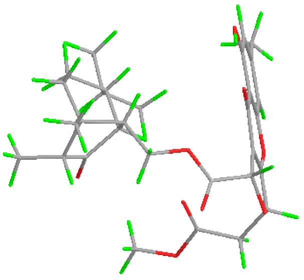

12aB

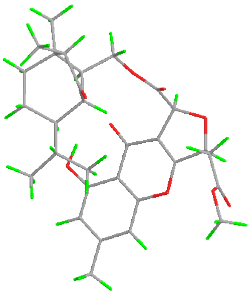

12aC

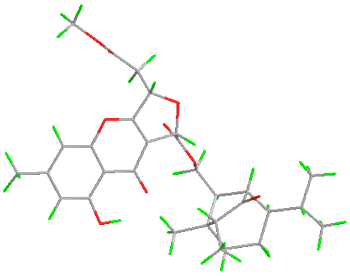

12aD

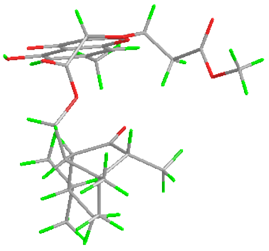

12aE

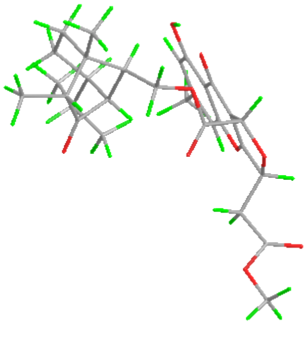

12aF

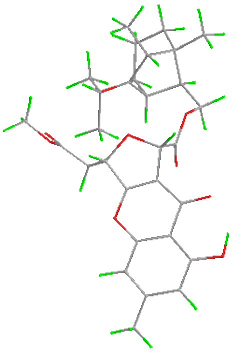

12aG

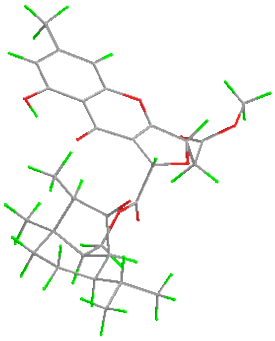

12aH

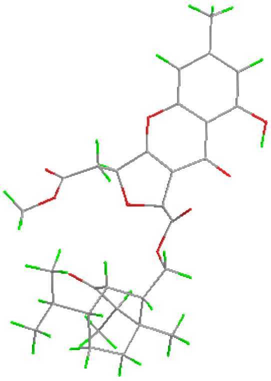

12aI

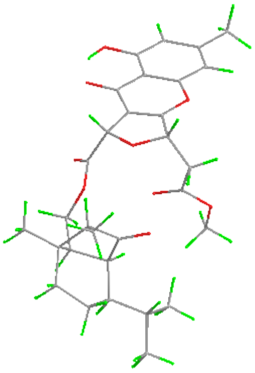

12aJ

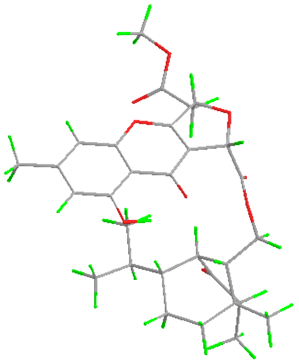

12aK

Table S6. Energy analysis for conformers of **12bA~12bF** at B3LYP/6-31G(d) level in the gas phase

Species	*E′*=*E*+*ZPE*	*E*	*H*	*G*	*ΔG*	*ΔE*(kcal/mol)	*PE*%
**12bA**	-1841.479288	-1841.442477	-1841.441533	-1841.547737	0.002644	1.659135	4.06%
**12bB**	-1841.480583	-1841.443646	-1841.442702	-1841.549573	0.000808	0.507028	28.40%
**12bC**	-1841.479168	-1841.441893	-1841.440948	-1841.550381	0	0	66.88%
**12bD**	-1841.470431	-1841.433084	-1841.432139	-1841.542772	0.007609	4.77472	0.02%
**12bE**	-1841.470006	-1841.432677	-1841.431732	-1841.542051	0.00833	5.227154	0.01%
**12bF**	-1841.475978	-1841.439043	-1841.438098	-1841.545978	0.004403	2.762924	0.63%

E, *E′*, *H*, *G*: total energy, total energy with zero point energy (*ZPE*), enthalpy, and Gibbs free energy

Figure S11. Main conformers of **12b** in ECD

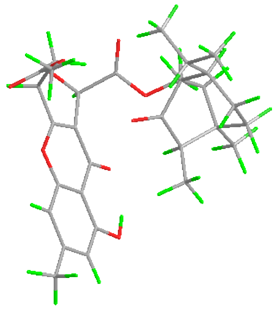

12bA

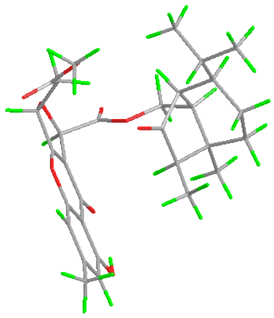

12bB

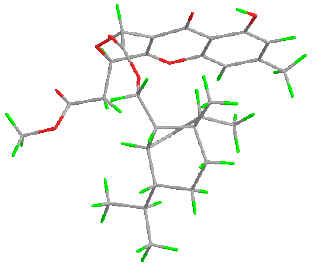

12bC

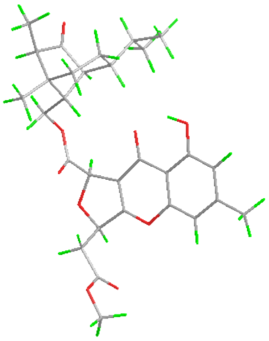

12bD

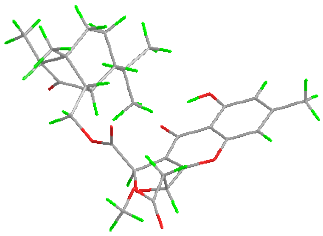

12bE

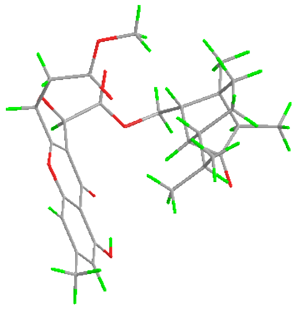

12bF

Table S7. Energy analysis for conformers of **12cA~12cM** at B3LYP/6-31G(d) level in the gas phase

Species	*E′=E+ZPE*	*E*	*H*	*G*	*ΔG*	*ΔE*(kcal/mol)	*PE%*
**12cA**	-1841.477567	-1841.440453	-1841.439509	-1841.547406	0.000798	0.500752581	19.02%
**12cB**	-1841.480266	-1841.443481	-1841.442537	-1841.548204	0	0	44.31%
**12cC**	-1841.475594	-1841.438502	-1841.437558	-1841.54501	0.003194	2.004265343	1.50%
**12cD**	-1841.469323	-1841.431763	-1841.430818	-1841.542825	0.005379	3.3753736	0.15%
**12cE**	-1841.476806	-1841.439623	-1841.438679	-1841.546969	0.001235	0.774974232	11.97%
**12cF**	-1841.474586	-1841.437285	-1841.436341	-1841.54594	0.002264	1.420681508	4.02%
**12cG**	-1841.473561	-1841.436386	-1841.435442	-1841.544093	0.004111	2.579691554	0.57%
**12cH**	-1841.469825	-1841.43259	-1841.431645	-1841.5412	0.007004	4.395076538	0.03%
**12cI**	-1841.468618	-1841.431083	-1841.430139	-1841.541659	0.006545	4.107049677	0.04%
**12cJ**	-1841.477451	-1841.440311	-1841.439367	-1841.547357	0.000847	0.531500547	18.06%
**12cK**	-1841.471134	-1841.434062	-1841.433118	-1841.541873	0.006331	3.972762645	0.05%
**12cL**	-1841.474177	-1841.437092	-1841.436147	-1841.543282	0.004922	3.088601759	0.24%
**12cM**	-1841.467596	-1841.430163	-1841.429219	-1841.541311	0.006893	4.325422983	0.03%

E, *E′*, *H*, *G*: total energy, total energy with zero point energy (*ZPE*), enthalpy, and Gibbs free energy

Figure S12. Main conformers of **12c** in ECD

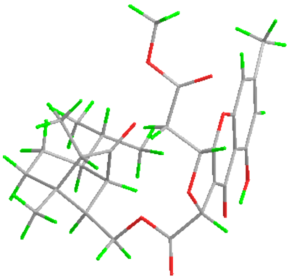

12cA

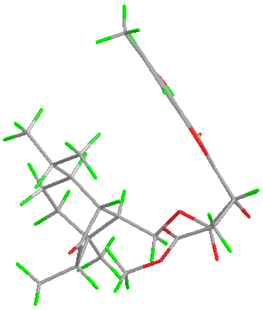

12cB

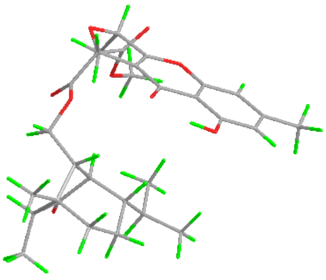

12cC

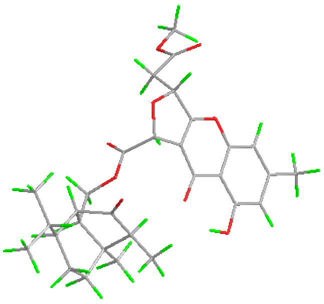

12cD

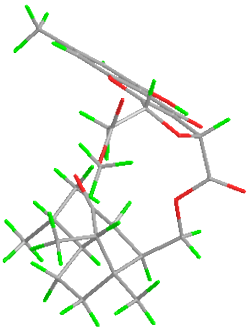

12cE

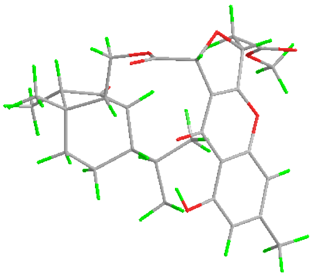

12cF

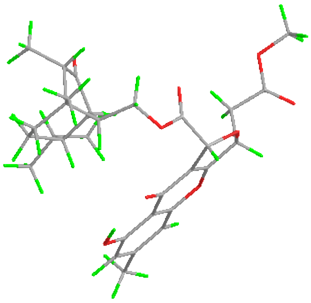

12cG

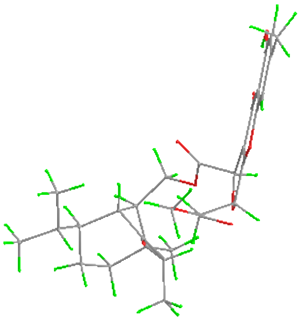

12cH

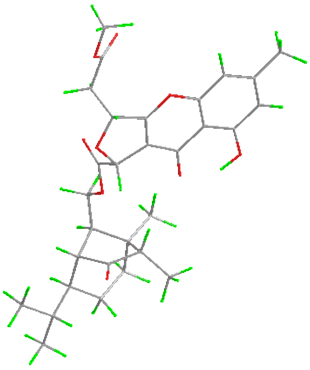

12cI

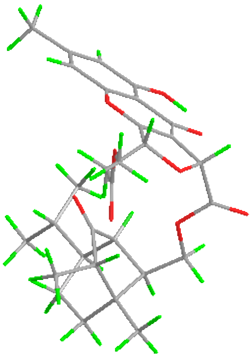

12cJ

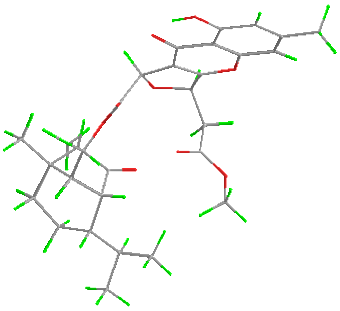

12cK

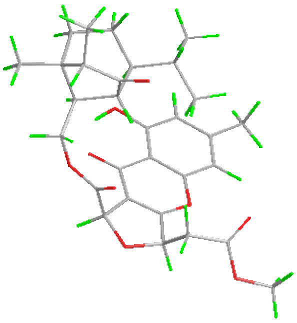

12cL

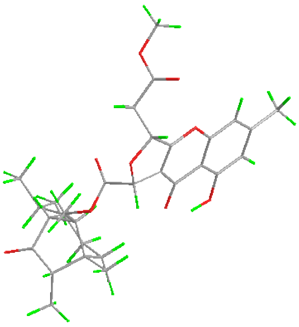

12cM

Table S8. Energy analysis for conformers of **12dA~12dF** at B3LYP/6-31G(d) level in the gas phase

Species	*E′*=*E*+*ZPE*	*E*	*H*	*G*	*ΔG*	*ΔE*(kcal/mol)	*PE*%
**12dA**	-1841.479288	-1841.442477	-1841.441533	-1841.547737	0.002647	1.661017646	3.03%
**12dB**	-1841.480583	-1841.443647	-1841.442703	-1841.54957	0.000814	0.510792733	21.12%
**12dC**	-1841.479169	-1841.441893	-1841.440949	-1841.550384	0	0	50.05%
**12dD**	-1841.475978	-1841.439043	-1841.438099	-1841.545975	0.004409	2.766689385	0.47%
**12dE**	-1841.480098	-1841.44304	-1841.442095	-1841.549741	0.000643	0.403488608	25.32%
**12dF**	-1841.470432	-1841.43309	-1841.432146	-1841.542697	0.007687	4.823665526	0.01%

E, *E′*, *H*, *G*: total energy, total energy with zero point energy (*ZPE*), enthalpy, and Gibbs free energy

Figure S13. Main conformers of **12d** in ECD

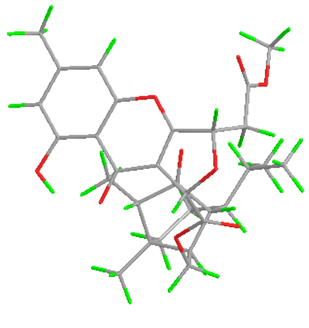

12dA

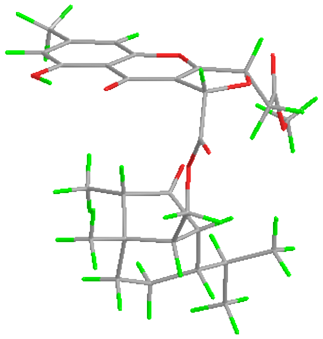

12dB

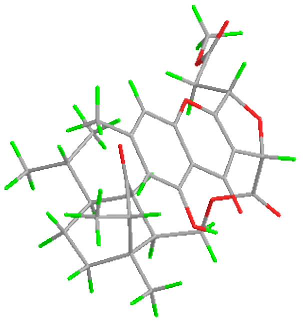

12dC

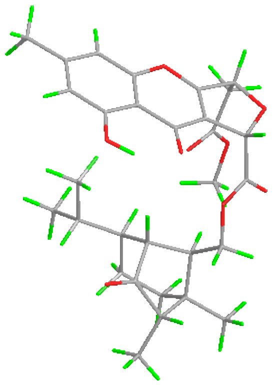

12dD

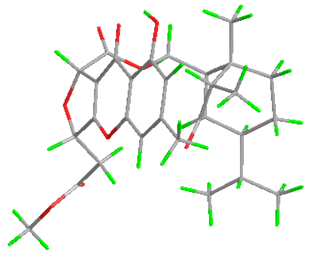

12dE

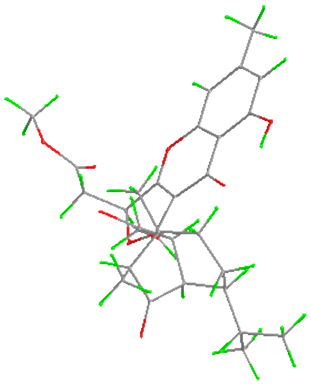

12dF

S2.6. Computational details for 12a and 12d (NMR)

All the optimized conformers were subjected to Gauge Independent Atomic Orbital (GIAO) calculations of their ^{13}C NMR chemical shifts using density functional theory (DFT) at the mPW1PW91/6-311+G (d, p) level with the PCM model in methanol. The calculated NMR data of these conformers were averaged according to the Boltzmann distribution theory and their relative Gibbs free energy. The ^{13}C NMR chemical shifts for TMS were also calculated by the same procedures and used as the reference. After calculation, the experimental and calculated data were evaluated by the improved probability DP4+ method.

Table S9. DP4+ analysis results of **12a** (Isomer 1) and **12d** (Isomer 2)

Functional		Solvent?		Basis Set		Type of Data	
mPW1PW91		PCM		6-311+G(d,p)		Shielding Tensors	
		DP4+	100.00%	0.00%	–	–	–
Nuclei	sp2?	xperimenta	Isomer 1	Isomer 2	Isomer 3	Isomer 4	Isomer 5
C	x	221.6	-47.3	-46.8			
C		50.6	131.1	128.2			
C		42.1	141.2	136.6			
C		36.1	148.6	144.6			
C		26.0	157.4	154.0			
C		50.2	135.4	129.2			
C		51.5	131.9	128.0			
C		22.1	163.7	160.1			
C		30.0	152.7	150.1			
C		20.4	166.5	160.5			
C		21.3	164.3	161.8			
C		6.5	178.6	174.9			
C		51.6	131.6	128.7			
C		65.3	120.8	116.1			
C	x	161.1	19.6	18.7			
C	x	113.6	68.7	67.1			
C	x	147.6	32.1	30.8			
C	x	108.1	74.7	72.3			
C	x	157.4	23.2	22.1			
C		78.2	103.4	101.2			
C		37.7	144.9	140.1			
C	x	169.5	9.2	10.9			
C		79.5	102.6	99.4			
C	x	114.5	67.3	64.9			
C	x	178.2	4.1	3.4			
C	x	109.0	73.2	71.7			
C	x	167.7	10.3	10.5			
C		22.5	162.9	158.5			
C	x	169.4	8.3	7.8			
C		52.4	131.8	129.7			

Functional	Solvent?		Basis Set		Type of Data	
mPW1PW91	PCM		6-311+G(d,p)		Shielding Tensors	
	Isomer 1	Isomer 2	Isomer 3	Isomer 4	Isomer 5	Isomer 6
sDP4+ (H data)	–	–	–	–	–	–
sDP4+ (C data)	39.00%	61.00%	–	–	–	–
sDP4+ (all data)	39.00%	61.00%	–	–	–	–
uDP4+ (H data)	–	–	–	–	–	–
uDP4+ (C data)	100.00%	0.00%	–	–	–	–
uDP4+ (all data)	100.00%	0.00%	–	–	–	–
DP4+ (H data)	–	–	–	–	–	–
DP4+ (C data)	100.00%	0.00%	–	–	–	–
DP4+ (all data)	100.00%	0.00%	–	–	–	–

S2.7. Computational details for bipolarithone D (13) (ECD)

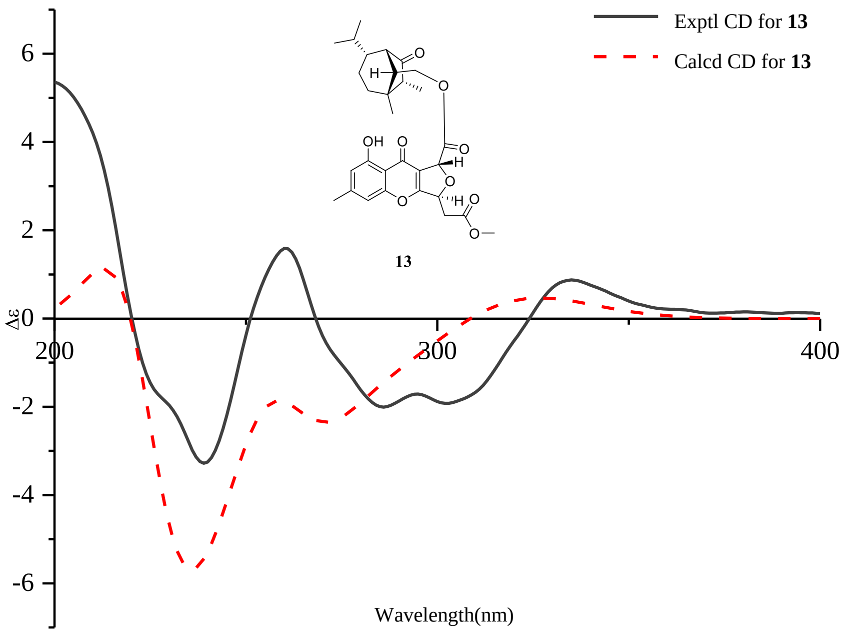

Figure S14. Calculated ECD spectra for **13** at the B3LYP/6-311G(d) level in methanol with IEFPCM model (σ = 0.25 eV, UV shift -7 nm). Experimental CD spectra of **13** (black line) in MeOH.

Table S10. Energy analysis for conformers of **13A~13I** at B3LYP/6-31G(d) level in the gas phase

Species	*E′=E+ZPE*	*E*	*H*	*G*	*ΔG*	*ΔE*(kcal/mol)	*PE%*
**13A**	-1841.468163	-1841.430722	-1841.429777	-1841.540899	0.008079	5.06964925	0.01%
**13B**	-1841.476079	-1841.438801	-1841.437856	-1841.547017	0.001961	1.230546129	9.76%
**13C**	-1841.472092	-1841.434953	-1841.434009	-1841.543804	0.005174	3.246734153	0.32%
**13D**	-1841.469919	-1841.43262	-1841.431676	-1841.542167	0.006811	4.273967204	0.06%
**13E**	-1841.476788	-1841.439463	-1841.438519	-1841.548978	0	0	78.03%
**13F**	-1841.473712	-1841.436572	-1841.435628	-1841.545092	0.003886	2.438501917	1.27%
**13G**	-1841.474043	-1841.436633	-1841.435689	-1841.546529	0.002449	1.536770766	5.82%
**13H**	-1841.472688	-1841.435529	-1841.434585	-1841.544133	0.004845	3.040283527	0.46%
**13I**	-1841.475408	-1841.438401	-1841.437457	-1841.546233	0.002745	1.722513578	4.25%

E, *E′*, *H*, *G*: total energy, total energy with zero point energy (*ZPE*), enthalpy, and Gibbs free energy

Figure S15. Main conformers of **13** in ECD

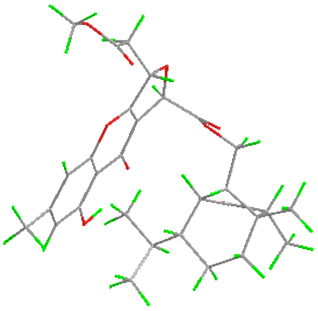

13A

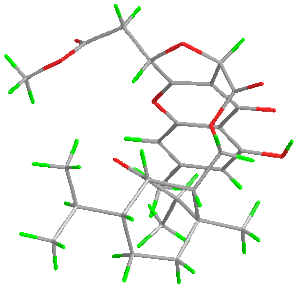

13b

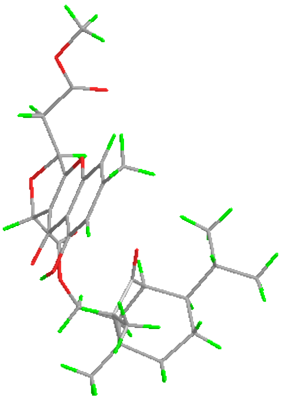

13C

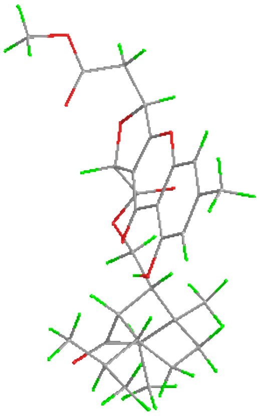

13D

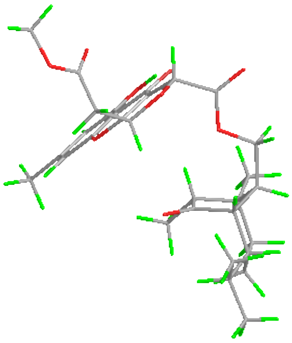

13E

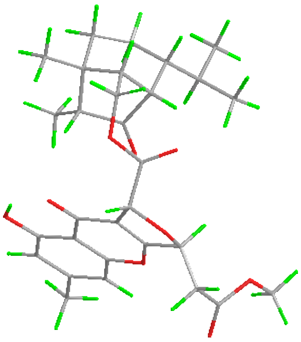

13F

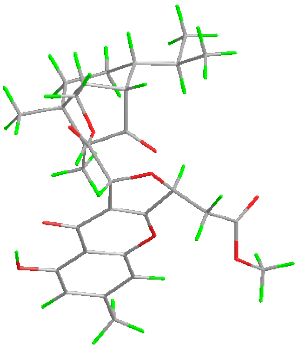

13G

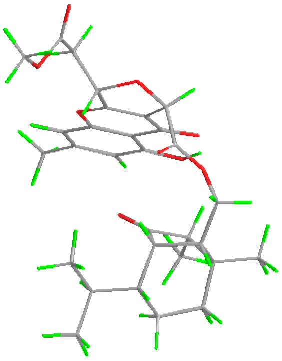

13H

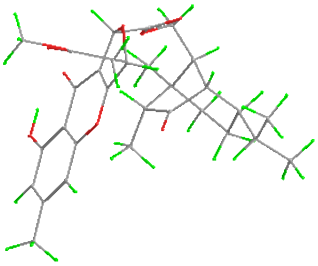

13I
